# Supplementary material for: From sunrise to sunset: Exploring landscape preference through global reactions to ephemeral events captured in georeferenced social media
Source: PLoS One. 2023 Feb 22;18(2):e0280423. doi: 10.1371/journal.pone.0280423 (PMC9946259; doi:10.1371/journal.pone.0280423)
Supplement: S1 File — (HTML) [file pone.0280423.s001.html]

01\_grid\_agg


# Sunset Sunrise Worldwide: Preparations ¶

*Alexander Dunkel, TU Dresden, Institute of Cartography; Maximilian Hartmann, Universität Zürich (UZH), Geocomputation*

---

•••

Out[1]:

Last updated: Jan-16-2023, Carto-Lab Docker Version 0.9.0

# Introduction¶

Based on data from Instagram and Flickr, we'll explore global reactions to sunsets and sunrises in these notebooks.

This is the first notebook in a series of nine notebooks:

1. the grid aggregation notebook (01\_gridagg.ipynb) is used to aggregate data from HLL sets at GeoHash 5 to a 100x100km grid
2. the visualization notebook (02\_visualization.ipynb) is used to create interactive maps, with additional information shown on hover
3. the chimaps notebook (03\_chimaps.ipynb) shows how to compute the chi square test per bin and event (sunset/sunrise).
4. the results notebook (04\_combine.ipynb) shows how to combine results from sunset/sunrise into a single interactive map.
5. Notebooks 5 to 9 are used for creating additional graphics and statistics.

- Projections: crs.Mollweide() (epsg code ESRI:54009)

Use **Shift+Enter** to walk through the Notebook

**Install dependencies**

- Either use the Carto-Lab Docker Container
- or use the following steps to create an env in conda for running this notebook. Suggested using `miniconda` in WSL.

**Manual installation of dependencies**

```
conda create -n sunset_env -c conda-forge
conda activate sunset_env
conda config --env --set channel_priority strict
conda config --show channel_priority # verify
# jupyter dependencies
conda install -c conda-forge 'ipywidgets=7.5.*' jupyterlab jupyterlab-git \
    jupytext jupyter_contrib_nbextensions jupyter_nbextensions_configurator
# visualization dependencies
conda install -c conda-forge geopandas jupyterlab "geoviews-core=1.9.5" \
    descartes mapclassify jupyter_contrib_nbextensions xarray \
    colorcet memory_profiler seaborn
# privacy aware dependencies
conda install -c conda-forge python-dotenv psycopg2
```

  
to upgrade later, use:

```
conda upgrade -n sunset_env --all -c conda-forge
```

  
Pinning geoviews to 1.9.5 should result in packages installed that are compatible with the code herein. For full compatibility,
install exact versions as shown below ("Plot used package versions for future use").
  
To start the jupyter lab server:

```
conda activate sunset_env
jupyter lab
```

# Preparations¶

## Load dependencies¶

In [2]:

```
import os
import csv
import sys
import colorcet
import psycopg2 # Postgres API
import geoviews as gv
import holoviews as hv
import mapclassify as mc
import geopandas as gp
import pandas as pd
import numpy as np
import matplotlib.pyplot as plt
import geoviews.feature as gf
from pathlib import Path
from collections import namedtuple
from pathlib import Path
from typing import List, Tuple, Dict, Optional
from pyproj import Transformer, CRS, Proj
from geoviews import opts
from shapely.geometry import shape, Point, Polygon
from shapely.ops import transform
from cartopy import crs
from matplotlib import colors
from IPython.display import clear_output, display, HTML
from bokeh.models import HoverTool, FuncTickFormatter, FixedTicker
# optionally, enable shapely.speedups 
# which makes some of the spatial 
# queries running faster
import shapely.speedups as speedups
from shapely import geometry
```

Load helper module from `../py/module/tools.py`. This also allows us to import code from other jupyter notebooks, synced to `*.py` with jupytext.

In [3]:

```
module_path = str(Path.cwd().parents[0] / "py")
if module_path not in sys.path:
    sys.path.append(module_path)
from modules import tools, preparations
```

Initialize Bokeh and shapely.speedups

In [4]:

```
preparations.init_imports()
```

## Parameters¶

Define initial parameters that affect processing of data and graphics in all notebooks.

In [5]:

```
GRID_SIZE_METERS = 100000
"""The size of grid cells in meters 
(spatial accuracy of worldwide measurement)"""
CHUNK_SIZE = 5000000
"""Process x number of hll records per chunk.
Increasing this number will consume more memory,
but reduce processing time because less SQL queries
are needed."""
EPSG_CODE = 54009
CRS_PROJ = f"esri:{EPSG_CODE}"
"""Target projection: Mollweide (epsg code).
note: Mollweide defined by _esri_
in epsg.io's database"""
CRS_WGS = "epsg:4326"
"""Input projection (Web Mercator)"""
OUTPUT = Path.cwd().parents[0] / "out"
"""Define path to output directory (figures etc.)""";
```

We want to modify intermediate file names if not using a 100 km grid:

In [6]:

```
km_size = GRID_SIZE_METERS/1000
if km_size == 100:
    km_size_str = ""
else:
    km_size_str = f"_{km_size:.0f}km"
```

**Create paths**

In [7]:

```
def create_paths(
    output: str = OUTPUT, subfolders: List[str] = ["html", "pickles", "csv", "figures", "svg", "pdf"]):
    """Create subfolder for results to be stored"""
    output.mkdir(exist_ok=True)
    for subfolder in subfolders:
        Path(OUTPUT / f'{subfolder}{km_size_str}').mkdir(exist_ok=True)
```

In [8]:

```
create_paths()
```

**Plot used package versions for future use:**

•••

| package | xarray | ipython | matplotlib | pyproj | bokeh | mapclassify | cloudpickle | numpy | holoviews | geoviews | Fiona | Shapely | pandas | Cartopy | geopandas |
| --- | --- | --- | --- | --- | --- | --- | --- | --- | --- | --- | --- | --- | --- | --- | --- |
| version | 2022.6.0 | 8.4.0 | 3.5.3 | 3.2.1 | 2.4.3 | 2.4.3 | 2.1.0 | 1.22.4 | 1.14.8 | 1.9.5 | 1.8.20 | 1.7.1 | 1.4.3 | 0.20.1 | 0.11.1 |

## Folder structure and paths to data¶

The notebooks are synced to markdown and python files, through jupytext, which in turn are managed in a git repository.

The data is exported from hlldb and stored as CSV files.

This notebook is based on the following folder structure:

```
.
├── 00_hll_data/
|   ├── flickr_sunrise_hll.csv
|   ├── flickr_sunset_hll.csv
|   ├── flickr_all_hll.csv
|   ├── instagram_sunrise_hll.csv
|   ├── instagram_sunset_hll.csv
|   └── instagram_random_hll.csv
|   
└── 01_analysis
    ├──notebooks/
    |    ├──01_grid_agg.ipynb
    |    ├──02_visualization.ipynb
    |    ├──03_chimaps.ipynb
    |    ├──04_combine.ipynb
    |    └──...
    ├──out/
    |    ├──csv/
    |    ├──figures/
    |    ├──html/
    |    ├──...
    |    └──pickles/
    ├──md/
    ├──py/
    ├──.git
    └── README.md
```

To make this notebook cross-compatible for Linux and Windows users, use pathlib to format (`/`) paths:

In [10]:

```
root = Path.cwd().parents[1] / "00_hll_data"
```

In [11]:

```
SUNRISE_FLICKR = root / "flickr_sunrise_hll.csv"
SUNSET_FLICKR = root / "flickr_sunset_hll.csv"
ALL_FLICKR = root / "flickr_all_hll.csv"
SUNRISE_INSTAGRAM = root / "instagram_sunrise_hll.csv"
SUNSET_INSTAGRAM = root / "instagram_sunset_hll.csv"
RANDOM_INSTAGRAM = root / "instagram_random_hll.csv"
```

## Dataset Preview¶

First, some statistics for these files:

•••

| name | SUNRISE\_FLICKR | SUNSET\_FLICKR | ALL\_FLICKR | SUNRISE\_INSTAGRAM | SUNSET\_INSTAGRAM | RANDOM\_INSTAGRAM |
| --- | --- | --- | --- | --- | --- | --- |
| size | 12.36 MB | 11.37 MB | 84.33 MB | 12.92 MB | 28.96 MB | 57.84 MB |
| records | 13,748 | 20,310 | 71,480 | 20,124 | 25,932 | 27,550 |

```
CPU times: user 154 ms, sys: 40.3 ms, total: 194 ms
Wall time: 196 ms
```

Note that HLL-Data is already pre-aggregated using a GeoHash of 5. Thus, the actual number of records is lower than the total number of posts reflected in these HLL sets.

Data structure of sunrise and sunset is the same. Dataset 'Flickr\_All\_world' differs. Example structure of `SUNRISE_INSTAGRAM` (first 3 records):

In [13]:

```
pd.set_option('display.max_colwidth', 150)
tools.record_preview_hll(SUNRISE_FLICKR)
```

|  | 0 |
| --- | --- |
| Record 0 |  |
| latitude | -90.0 |
| longitude | -180.0 |
| user\_hll | \x128b7f7b5142a290c53d3b |
| post\_hll | \x128b7f468c0387651db7dc |
| date\_hll | \x128b7f88b65af15f32cf2e |

|  | 0 |
| --- | --- |
| Record 1 |  |
| latitude | -90.0 |
| longitude | -180.0 |
| user\_hll | \x128b7f76a0d9cdb8ac1803 |
| post\_hll | \x128b7f3ed9f976a3c25807 |
| date\_hll | \x128b7f51b5232fbc0e5976 |

|  | 0 |
| --- | --- |
| Record 2 |  |
| latitude | -17.04058337861201 |
| longitude | -178.6495358217821 |
| user\_hll | \x128b7f456fff7d4cfaaef0 |
| post\_hll | \x128b7fb7e998904019d42bbb6758efd6cd34fcc5264e462e203f21fa8c14cb2eeaf20d107b13afe355d5ab1fde884ab84690f23d43637eddfda828.. |
| date\_hll | \x128b7fe918837433d54ba3fb85994c963d9755004bea8c286f36ed032a4bb91f374ade0b5f24beaf03ba3d4c38518aa2022c186152578f538c1ea5.. |

## Preparations for HLL calculation¶

Password and username for connecting to local hllworker are loaded from environment.

Define credentials as environment variables and load here

In [14]:

```
DB_USER = "postgres"
DB_PASS = os.getenv('POSTGRES_PASSWORD')
# set connection variables
DB_HOST = "lbsn-hlldb-sunset"
DB_PORT = "5432"
DB_NAME = "lbsn-hlldb"
```

Connect to empty Postgres database running HLL Extension:

In [15]:

```
DB_CONN = psycopg2.connect(
        host=DB_HOST,
        port=DB_PORT ,
        dbname=DB_NAME,
        user=DB_USER,
        password=DB_PASS
)
DB_CONN.set_session(
    readonly=True)
DB_CALC = tools.DbConn(
    DB_CONN)
CUR_HLL = DB_CONN.cursor()
```

Test connection:

In [16]:

```
CUR_HLL.execute("SELECT 1;")
print(CUR_HLL.statusmessage)
```

```
SELECT 1
```

## Create Grid¶

1. Define Mollweide crs string for pyproj/Proj4 and WGS1984 for Social Media imports

In [17]:

```
# define Transformer ahead of time
# with xy-order of coordinates
PROJ_TRANSFORMER = Transformer.from_crs(
    CRS_WGS, CRS_PROJ, always_xy=True)

# also define reverse projection
PROJ_TRANSFORMER_BACK = Transformer.from_crs(
    CRS_PROJ, CRS_WGS, always_xy=True)
```

1. create bounds from WGS1984 and project to Mollweide

In [18]:

```
XMIN = PROJ_TRANSFORMER.transform(
    -180, 0)[0]
XMAX = PROJ_TRANSFORMER.transform(
    180, 0)[0]
YMAX = PROJ_TRANSFORMER.transform(
    0, 90)[1]
YMIN = PROJ_TRANSFORMER.transform(
    0, -90)[1]
```

In [19]:

```
print(f'Projected bounds: {[XMIN, YMIN, XMAX, YMAX]}')
```

```
Projected bounds: [-18040095.696147293, -9020047.848073646, 18040095.696147293, 9020047.848073646]
```

1. Create 100x100 km (e.g.) Grid

In [20]:

```
def create_grid_df(
    grid_size: int = GRID_SIZE_METERS,
    xmin: float = XMIN, ymin: float = YMIN, 
    xmax: float = XMAX, ymax: float = YMAX,
    report: bool = None,
    return_rows_cols: bool = None):
    """Creates dataframe polygon grid based on width and length in Meters"""
    width = grid_size
    length = grid_size
    cols = list(range(int(np.floor(xmin)), int(np.ceil(xmax)), width))
    rows = list(range(int(np.floor(ymin)), int(np.ceil(ymax)), length))
    if report:
        print(len(cols))
        print(len(rows))
    rows.reverse()
    
    polygons = []
    for x in cols:
        for y in rows:
            # combine to tuple: (x,y, poly)
            # and append to list
            polygons.append(
                (x, y, 
                 Polygon([
                     (x,y), 
                     (x+width, y), 
                     (x+width, y-length), 
                     (x, y-length)])) )
    # create a pandas dataframe
    # from list of tuples
    grid = pd.DataFrame(polygons)
    # name columns
    col_labels=['xbin', 'ybin', 'bin_poly']
    grid.columns = col_labels
    # use x and y as index columns
    grid.set_index(['xbin', 'ybin'], inplace=True)
    if return_rows_cols:
        return grid, rows, cols
    return grid
```

In [21]:

```
grid, rows, cols = create_grid_df(
    grid_size=GRID_SIZE_METERS,
    report=True, return_rows_cols=True)
```

```
361
181
```

In [22]:

```
grid.head()
```

Out[22]:

|  |  | bin\_poly |
| --- | --- | --- |
| xbin | ybin |  |
| -18040096 | 8979952 | POLYGON ((-18040096 8979952, -17940096 8979952, -17940096 8879952, -18040096 8879952, -18040096 8979952)) |
|  | 8879952 | POLYGON ((-18040096 8879952, -17940096 8879952, -17940096 8779952, -18040096 8779952, -18040096 8879952)) |
|  | 8779952 | POLYGON ((-18040096 8779952, -17940096 8779952, -17940096 8679952, -18040096 8679952, -18040096 8779952)) |
|  | 8679952 | POLYGON ((-18040096 8679952, -17940096 8679952, -17940096 8579952, -18040096 8579952, -18040096 8679952)) |
|  | 8579952 | POLYGON ((-18040096 8579952, -17940096 8579952, -17940096 8479952, -18040096 8479952, -18040096 8579952)) |

Create a geodataframe from dataframe:

In [23]:

```
def grid_to_gdf(
    grid: pd.DataFrame, crs_proj: str = CRS_PROJ) -> gp.GeoDataFrame:
    """Convert grid pandas DataFrame to geopandas Geodataframe"""
    grid = gp.GeoDataFrame(
        grid.drop(
            columns=["bin_poly"]),
            geometry=grid.bin_poly)
    grid.crs = crs_proj
    return grid
```

In [24]:

```
grid = grid_to_gdf(grid)
```

Add columns for aggregation

In [25]:

```
def reset_metrics(
    grid: gp.GeoDataFrame,
    metrics: List[str] = [
        "postcount_est", "usercount_est", "userdays_est",
        "postcount_hll", "usercount_hll", "userdays_hll"],
    setzero: bool = None):
    """Remove columns from GeoDataFrame and optionally fill with 0"""
    for metric in metrics:
        try:
            grid.drop(metric, axis=1, inplace=True)
            grid.drop(f'{metric}_cat', axis=1, inplace=True)
        except KeyError:
            pass
        if setzero:
            grid.loc[:, metric] = 0
```

In [26]:

```
reset_metrics(grid)
display(grid)
```

|  |  | geometry |
| --- | --- | --- |
| xbin | ybin |  |
| -18040096 | 8979952 | POLYGON ((-18040096.000 8979952.000, -17940096.000 8979952.000, -17940096.000 8879952.000, -18040096.000 8879952.000, -18040096.000 8979952.000)) |
|  | 8879952 | POLYGON ((-18040096.000 8879952.000, -17940096.000 8879952.000, -17940096.000 8779952.000, -18040096.000 8779952.000, -18040096.000 8879952.000)) |
|  | 8779952 | POLYGON ((-18040096.000 8779952.000, -17940096.000 8779952.000, -17940096.000 8679952.000, -18040096.000 8679952.000, -18040096.000 8779952.000)) |
|  | 8679952 | POLYGON ((-18040096.000 8679952.000, -17940096.000 8679952.000, -17940096.000 8579952.000, -18040096.000 8579952.000, -18040096.000 8679952.000)) |
|  | 8579952 | POLYGON ((-18040096.000 8579952.000, -17940096.000 8579952.000, -17940096.000 8479952.000, -18040096.000 8479952.000, -18040096.000 8579952.000)) |
| ... | ... | ... |
| 17959904 | -8620048 | POLYGON ((17959904.000 -8620048.000, 18059904.000 -8620048.000, 18059904.000 -8720048.000, 17959904.000 -8720048.000, 17959904.000 -8620048.000)) |
|  | -8720048 | POLYGON ((17959904.000 -8720048.000, 18059904.000 -8720048.000, 18059904.000 -8820048.000, 17959904.000 -8820048.000, 17959904.000 -8720048.000)) |
|  | -8820048 | POLYGON ((17959904.000 -8820048.000, 18059904.000 -8820048.000, 18059904.000 -8920048.000, 17959904.000 -8920048.000, 17959904.000 -8820048.000)) |
|  | -8920048 | POLYGON ((17959904.000 -8920048.000, 18059904.000 -8920048.000, 18059904.000 -9020048.000, 17959904.000 -9020048.000, 17959904.000 -8920048.000)) |
|  | -9020048 | POLYGON ((17959904.000 -9020048.000, 18059904.000 -9020048.000, 18059904.000 -9120048.000, 17959904.000 -9120048.000, 17959904.000 -9020048.000)) |

65341 rows × 1 columns

**Read World geometries data**

In [27]:

```
%%time
world = gp.read_file(gp.datasets.get_path('naturalearth_lowres'), crs=CRS_WGS)
world = world.to_crs(CRS_PROJ)
```

```
CPU times: user 62.4 ms, sys: 0 ns, total: 62.4 ms
Wall time: 61.9 ms
```

### Preview Grid¶

In [28]:

```
base = grid.plot(figsize=(22,28), color='white', edgecolor='black', linewidth=0.1)
# combine with world geometry
plot = world.plot(ax=base)
```

# Prepare binary search¶

The aggregation speed is important here and we should not use polygon intersection. Since we're working with a regular grid and floating point numbers, a binary search is likely one of the fastest ways for our context. numpy.digitize provides a binary search, but it must be adapted to for the spatial context. A lat or lng value is assigned to the nearest bin matching. We get our lat and lng bins from our original Mollweide grid, which are regularly spaced at 100km interval. Note that we need to do two binary searches, for lat and for lng values.

## Create test points¶

In [29]:

```
testpoint = Point(8.546377, 47.392323)
testpoint2 = Point(13.726359, 51.028512)
testpoint3 = Point(13.71389, 46.52278) # Dreiländereck
gdf_testpoints = gp.GeoSeries([testpoint, testpoint2, testpoint3], crs=CRS_WGS)
# project geometries to Mollweide
gdf_testpoints_proj = gdf_testpoints.to_crs(CRS_PROJ)
display(gdf_testpoints_proj[0].x)
```

```
671646.5840955656
```

Preview map for testpoint

In [30]:

```
base = world.plot(figsize=(22,28), color='white', edgecolor='black', linewidth=0.1)
plot = gdf_testpoints_proj.plot(ax=base, markersize=10)
```

### Use np.digitize() to assign coordinates to the grid¶

> np.digitize is implemented in terms of np.searchsorted. This means that a binary search is used to bin the values, which scales much better for larger number of bins than the previous linear search. It also removes the requirement for the input array to be 1-dimensional.

Create 2 bins for each axis of existing Mollweide rows/cols grid:

In [31]:

```
ybins = np.array(rows)
xbins = np.array(cols)
```

Create 2 lists with a single entry (testpoint coordinate)

In [32]:

```
test_point_list_x = np.array([p.x for p in gdf_testpoints_proj])
test_point_list_y = np.array([p.y for p in gdf_testpoints_proj])
```

Find the nearest bin for x coordinate (returns the bin-index):

In [33]:

```
x_bin = np.digitize(test_point_list_x, xbins) - 1
display(x_bin)
```

```
array([187, 190, 191])
```

Check value of bin (the y coordinate) based on returned index:

In [34]:

```
testpoint_xbin_idx = xbins[[x_bin[x] for x in range(0, len(x_bin))]]
display(testpoint_xbin_idx)
```

```
array([ 659904,  959904, 1059904])
```

Repeat the same for y-testpoint:

In [35]:

```
y_bin = np.digitize(test_point_list_y, ybins) - 1
display(y_bin)
```

```
array([33, 29, 34])
```

In [36]:

```
testpoint_ybin_idx = ybins[[y_bin[x] for x in range(0, len(y_bin))]]
display(testpoint_ybin_idx)
```

```
array([5679952, 6079952, 5579952])
```

➡️ **759904** / **5579952** and **1059904** / **5979952** are indexes that we can use in our geodataframe index to return the matching grid-poly for each point

### Highlight Testpoint in Grid¶

Get grid-poly by index from testpoint

In [37]:

```
grid.loc[testpoint_xbin_idx[0], testpoint_ybin_idx[0]]
```

Out[37]:

```
geometry    POLYGON ((659904.000 5679952.000, 759904.000 5679952.000, 759904.000 5579952.000, 659904.000 5579952.000, 659904.000 5679952.000))
Name: (659904, 5679952), dtype: geometry
```

Convert shapely bin poly to Geoseries and plot

In [38]:

```
testpoint_grids = gp.GeoSeries(
    [grid.loc[testpoint_xbin_idx[x], testpoint_ybin_idx[x]].geometry for x in range(0,len(gdf_testpoints))])
testpoint_grids.plot()
```

Out[38]:

```
<AxesSubplot:>
```

### Preview map with testpoint and assigned bin¶

Set auto zoom with buffer:

In [39]:

```
minx, miny, maxx, maxy = testpoint_grids.total_bounds
buf = 1000000
```

In [40]:

```
# a figure with a 1x1 grid of Axes
fig, ax = plt.subplots(1, 1,figsize=(10,8))
ax.set_xlim(minx-buf, maxx+buf)
ax.set_ylim(miny-buf, maxy+buf)
base = world.plot(ax=ax, color='white', edgecolor='black', linewidth=0.1)
grid_base = testpoint_grids.plot(ax=base, facecolor='red', linewidth=0.1)
plot = gdf_testpoints_proj.plot(ax=grid_base, markersize=8, color='blue')
```

## Prepare functions¶

Now that it has been visually verified that the algorithm works, lets create functions for the main processing job.

In [41]:

```
def get_best_bins(
    search_values_x: np.array, search_values_y: np.array,
    xbins: np.array, ybins: np.array) -> Tuple[np.ndarray, np.ndarray]:
    """Will return best bin for a lat and lng input
    
    Note: prepare bins and values in correct matching projection
    
    Args:
        search_values_y: A list of projected latitude values
        search_values_x: A list of projected longitude values
        xbins: 1-d array of bins to snap lat/lng values
        ybins: 1-d array of bins to snap lat/lng values

    Returns:
        Tuple[int, int]: A list of tuples with 2 index positions for the best 
            matching bins for each lat/lng
    """
    xbins_idx = np.digitize(search_values_x, xbins, right=False)
    ybins_idx = np.digitize(search_values_y, ybins, right=False)
    return (xbins[xbins_idx-1], ybins[ybins_idx-1])
```

## Test with LBSN data¶

We're going to test the binning of coordinates on a small subset (Europe).

Prepare lat/lng tuple of lower left corner and upper right corner to crop sample map:

In [42]:

```
# Part of Italy and Sicily
bbox_italy = [
    7.8662109375, 36.84427318493909,
    19.31396484375, 43.89320031385282] 
bbox = bbox_italy
```

Calculate bounding box with 1000 km buffer. For that, project the bounding Box to Mollweide, apply the buffer, and project back to WGS1984:

In [43]:

```
# convert to Mollweide
minx, miny = PROJ_TRANSFORMER.transform(
    bbox_italy[0], bbox_italy[1])
maxx, maxy = PROJ_TRANSFORMER.transform(
    bbox_italy[2], bbox_italy[3])
# apply buffer and convetr back to WGS1984
min_buf = PROJ_TRANSFORMER_BACK.transform(minx-buf, miny-buf)
max_buf = PROJ_TRANSFORMER_BACK.transform(maxx+buf, maxy+buf)
bbox_italy_buf = [min_buf[0], min_buf[1], max_buf[0], max_buf[1]]
```

Select columns and types for improving speed

In [44]:

```
usecols = ['latitude', 'longitude', 'post_hll']
dtypes = {'latitude': float, 'longitude': float}
reset_metrics(grid)
```

### Load data¶

In [45]:

```
%%time
df = pd.read_csv(SUNRISE_FLICKR, usecols=usecols, dtype=dtypes, encoding='utf-8')
print(len(df))
```

```
13747
CPU times: user 66.3 ms, sys: 8.26 ms, total: 74.5 ms
Wall time: 74.2 ms
```

Filter on bounding box (Italy)

In [46]:

```
def filter_df_bbox(
    df: pd.DataFrame, bbox: Tuple[float, float, float, float],
    inplace: bool = True):
    """Filter dataframe with bbox on latitude and longitude column"""
    df.query(
        f'({bbox[0]} < longitude) & '
        f'(longitude <  {bbox[2]}) & '
        f'({bbox[1]} < latitude) & '
        f'(latitude < {bbox[3]})',
        inplace=True)
    # set index to asc integers
    if inplace:
        df.reset_index(inplace=True, drop=True)
        return
    return df.reset_index(inplace=False, drop=True)
```

Execute and count number of posts in the bounding box:

In [47]:

```
%%time
filter_df_bbox(df=df, bbox=bbox_italy_buf)
print(f"There're {len(df):,.0f} distinct lat-lng coordinates located within the bounding box.")
display(df.head())
```

```
There're 1,716 distinct lat-lng coordinates located within the bounding box.
```

|  | latitude | longitude | post\_hll |
| --- | --- | --- | --- |
| 0 | 47.460076 | -3.375952 | \x138b7f002101c103610404046104a20561066106c107a108840ae10b250e640f610fa1114211c1122114a316851701192219e11a031ae11b011b611be11c441ce11f011fe1202121... |
| 1 | 43.301242 | -3.223324 | \x138b7f00a201020121018102860301034103a104c1058205a206c106e107210741078407c50861088408a108c1094109e50a410a830bc20c620ca50ce10d630e810f82100310a211... |
| 2 | 42.845901 | -3.208339 | \x128b7f84712f04c3db0d119b53d25b37e97603a37836949509177ca81432cd0f5dacdab7a735d63848ff0bc5e15b18fb6abbc1cc0b68c570b2ec09ce83d4c3e13e1ad3d55a701078... |
| 3 | 42.391809 | -3.193704 | \x128b7f8af75c1f6a059ac18cef3cdd8250c18e8d054fcdb03d6dfc8e6af9b8d22ffd8d9190f09be294b73091e5385e28042f08a8aceebf92973a2ba9433b17d11fa963aaec4efbf6... |
| 4 | 41.487267 | -3.165442 | \x128b7f94bff98b0e5b0d3ff2dc20caf01b75a647bea732f50bcb50 |

```
CPU times: user 6.82 ms, sys: 3.75 ms, total: 10.6 ms
Wall time: 10.1 ms
```

### Project coordinates to Mollweide¶

In [48]:

```
def proj_df(df, proj_transformer: Transformer = PROJ_TRANSFORMER):
    """Project pandas dataframe latitude and longitude decimal degrees
    using predefined proj_transformer"""
    if 'longitude' not in df.columns:
        return
    xx, yy = proj_transformer.transform(
        df['longitude'].values, df['latitude'].values)
    # assign projected coordinates to
    # new columns x and y
    # the ':' means: replace all values in-place
    df.loc[:, "x"] = xx
    df.loc[:, "y"] = yy
    # Drop WGS coordinates
    df.drop(columns=['longitude', 'latitude'], inplace=True)
```

In [49]:

```
%%time
proj_df(df)
print(f'Projected {len(df.values)} coordinates')
display(df.head())
```

```
Projected 1716 coordinates
```

|  | post\_hll | x | y |
| --- | --- | --- | --- |
| 0 | \x138b7f002101c103610404046104a20561066106c107a108840ae10b250e640f610fa1114211c1122114a316851701192219e11a031ae11b011b611be11c441ce11f011fe1202121... | -265096.0 | 5604952.0 |
| 1 | \x138b7f00a201020121018102860301034103a104c1058205a206c106e107210741078407c50861088408a108c1094109e50a410a830bc20c620ca50ce10d630e810f82100310a211... | -265096.0 | 5154952.0 |
| 2 | \x128b7f84712f04c3db0d119b53d25b37e97603a37836949509177ca81432cd0f5dacdab7a735d63848ff0bc5e15b18fb6abbc1cc0b68c570b2ec09ce83d4c3e13e1ad3d55a701078... | -265096.0 | 5104952.0 |
| 3 | \x128b7f8af75c1f6a059ac18cef3cdd8250c18e8d054fcdb03d6dfc8e6af9b8d22ffd8d9190f09be294b73091e5385e28042f08a8aceebf92973a2ba9433b17d11fa963aaec4efbf6... | -265096.0 | 5054952.0 |
| 4 | \x128b7f94bff98b0e5b0d3ff2dc20caf01b75a647bea732f50bcb50 | -265096.0 | 4954952.0 |

```
CPU times: user 8.15 ms, sys: 15 µs, total: 8.17 ms
Wall time: 7.76 ms
```

### Perform the bin assignment¶

In [50]:

```
%%time
xbins_match, ybins_match = get_best_bins(
    search_values_x=df['x'].to_numpy(),
    search_values_y=df['y'].to_numpy(),
    xbins=xbins, ybins=ybins)
```

```
CPU times: user 357 µs, sys: 41 µs, total: 398 µs
Wall time: 363 µs
```

In [51]:

```
len(xbins_match)
```

Out[51]:

```
1716
```

In [52]:

```
xbins_match[:10]
```

Out[52]:

```
array([-340096, -340096, -340096, -340096, -340096, -340096, -340096,
       -340096, -340096, -340096])
```

In [53]:

```
ybins_match[:10]
```

Out[53]:

```
array([5679952, 5179952, 5179952, 5079952, 4979952, 4979952, 4879952,
       4879952, 4779952, 4779952])
```

## A: Estimated Post Count per grid¶

Attach target bins to original dataframe. The `:` means: modify all values in-place

In [54]:

```
df.loc[:, 'xbins_match'] = xbins_match
df.loc[:, 'ybins_match'] = ybins_match
# set new index column
df.set_index(['xbins_match', 'ybins_match'], inplace=True)
# drop x and y columns not needed anymore
df.drop(columns=['x', 'y'], inplace=True)
```

In [55]:

```
df.head()
```

Out[55]:

|  |  | post\_hll |
| --- | --- | --- |
| xbins\_match | ybins\_match |  |
| -340096 | 5679952 | \x138b7f002101c103610404046104a20561066106c107a108840ae10b250e640f610fa1114211c1122114a316851701192219e11a031ae11b011b611be11c441ce11f011fe1202121... |
|  | 5179952 | \x138b7f00a201020121018102860301034103a104c1058205a206c106e107210741078407c50861088408a108c1094109e50a410a830bc20c620ca50ce10d630e810f82100310a211... |
|  | 5179952 | \x128b7f84712f04c3db0d119b53d25b37e97603a37836949509177ca81432cd0f5dacdab7a735d63848ff0bc5e15b18fb6abbc1cc0b68c570b2ec09ce83d4c3e13e1ad3d55a701078... |
|  | 5079952 | \x128b7f8af75c1f6a059ac18cef3cdd8250c18e8d054fcdb03d6dfc8e6af9b8d22ffd8d9190f09be294b73091e5385e28042f08a8aceebf92973a2ba9433b17d11fa963aaec4efbf6... |
|  | 4979952 | \x128b7f94bff98b0e5b0d3ff2dc20caf01b75a647bea732f50bcb50 |

The next step is to union hll sets and (optionally) return the cardinality (the number of distinct elements). This can only be done by connecting to a postgres database with HLL extension installed. We're using our hlldb here, but it is equally possible to connect to an empty Postgres DB such as pg-hll-empty docker container.

In [56]:

```
def union_hll(
    hll_series: pd.Series, cardinality: bool = True,
    db_calc: tools.DbConn = DB_CALC) -> pd.Series:
    """HLL Union and (optional) cardinality estimation from series of hll sets
    based on group by composite index.

        Args:
        hll_series: Indexed series (bins) of hll sets. 
        cardinality: If True, returns cardinality (counts). Otherwise,
            the unioned hll set will be returned.
            
    The method will combine all groups of hll sets first,
        in a single SQL command. Union of hll hll-sets belonging 
        to the same group (bin) and (optionally) returning the cardinality 
        (the estimated count) per group will be done in postgres.
    
    By utilizing Postgres´ GROUP BY (instead of, e.g. doing 
        the group with numpy), it is possible to reduce the number
        of SQL calls to a single run, which saves overhead 
        (establishing the db connection, initializing the SQL query 
        etc.). Also note that ascending integers are used for groups,
        instead of their full original bin-ids, which also reduces
        transfer time.
    
    cardinality = True should be used when calculating counts in
        a single pass.
        
    cardinality = False should be used when incrementally union
        of hll sets is required, e.g. due to size of input data.
        In the last run, set to cardinality = True.
    """
    # group all hll-sets per index (bin-id)
    series_grouped = hll_series.groupby(
        hll_series.index).apply(list)
    # From grouped hll-sets,
    # construct a single SQL Value list;
    # if the following nested list comprehension
    # doesn't make sense to you, have a look at
    # spapas.github.io/2016/04/27/python-nested-list-comprehensions/
    # with a decription on how to 'unnest'
    # nested list comprehensions to regular for-loops
    hll_values_list = ",".join(
        [f"({ix}::int,'{hll_item}'::hll)" 
         for ix, hll_items
         in enumerate(series_grouped.values.tolist())
         for hll_item in hll_items])
    # Compilation of SQL query,
    # depending on whether to return the cardinality
    # of unioned hll or the unioned hll
    return_col = "hll_union"
    hll_calc_pre = ""
    hll_calc_tail = "AS hll_union"
    if cardinality:
        # add sql syntax for cardinality 
        # estimation
        # (get count distinct from hll)
        return_col = "hll_cardinality"
        hll_calc_pre = "hll_cardinality("
        hll_calc_tail = ")::int"
    db_query = f"""
        SELECT sq.{return_col} FROM (
            SELECT s.group_ix,
                   {hll_calc_pre}
                   hll_union_agg(s.hll_set)
                   {hll_calc_tail}
            FROM (
                VALUES {hll_values_list}
                ) s(group_ix, hll_set)
            GROUP BY group_ix
            ORDER BY group_ix ASC) sq
        """
    df = db_calc.query(db_query)
    # to merge values back to grouped dataframe,
    # first reset index to ascending integers
    # matching those of the returned df;
    # this will turn series_grouped into a DataFrame;
    # the previous index will still exist in column 'index'
    df_grouped = series_grouped.reset_index()
    # drop hll sets not needed anymore
    df_grouped.drop(columns=[hll_series.name], inplace=True)
    # append hll_cardinality counts 
    # using matching ascending integer indexes
    df_grouped.loc[df.index, return_col] = df[return_col]
    # set index back to original bin-ids
    df_grouped.set_index("index", inplace=True)
    # split tuple index to produce
    # the multiindex of the original dataframe
    # with xbin and ybin column names
    df_grouped.index = pd.MultiIndex.from_tuples(
        df_grouped.index, names=['xbin', 'ybin'])
    # return column as indexed pd.Series
    return df_grouped[return_col]
```

Optionally, split dataframe into chunks, so we're not the exceeding memory limit (e.g. use if memory < 16GB). A chunk size of 1 Million records is suitable for a computer with about 8 GB of memory and optional sparse HLL mode enabled. If sparse mode is disabled, decrease chunk\_size accordingly, to compensate for increased space.

In [57]:

```
%%time
chunked_df = [
    df[i:i+CHUNK_SIZE] for i in range(0, df.shape[0], CHUNK_SIZE)]
```

```
CPU times: user 157 µs, sys: 19 µs, total: 176 µs
Wall time: 180 µs
```

In [58]:

```
chunked_df[0].head()
```

Out[58]:

|  |  | post\_hll |
| --- | --- | --- |
| xbins\_match | ybins\_match |  |
| -340096 | 5679952 | \x138b7f002101c103610404046104a20561066106c107a108840ae10b250e640f610fa1114211c1122114a316851701192219e11a031ae11b011b611be11c441ce11f011fe1202121... |
|  | 5179952 | \x138b7f00a201020121018102860301034103a104c1058205a206c106e107210741078407c50861088408a108c1094109e50a410a830bc20c620ca50ce10d630e810f82100310a211... |
|  | 5179952 | \x128b7f84712f04c3db0d119b53d25b37e97603a37836949509177ca81432cd0f5dacdab7a735d63848ff0bc5e15b18fb6abbc1cc0b68c570b2ec09ce83d4c3e13e1ad3d55a701078... |
|  | 5079952 | \x128b7f8af75c1f6a059ac18cef3cdd8250c18e8d054fcdb03d6dfc8e6af9b8d22ffd8d9190f09be294b73091e5385e28042f08a8aceebf92973a2ba9433b17d11fa963aaec4efbf6... |
|  | 4979952 | \x128b7f94bff98b0e5b0d3ff2dc20caf01b75a647bea732f50bcb50 |

To test, process the first chunk:

In [59]:

```
%%time
cardinality_series = union_hll(chunked_df[0]["post_hll"])
```

```
CPU times: user 98 ms, sys: 12.2 ms, total: 110 ms
Wall time: 140 ms
```

In [60]:

```
cardinality_series.head()
```

Out[60]:

```
xbin     ybin   
-340096  4279952    26
         4479952    50
         4579952    58
         4779952     6
         4879952    52
Name: hll_cardinality, dtype: int64
```

Remove possibly existing result column in grid from previous run:

In [61]:

```
reset_metrics(grid, ["postcount_est"], setzero=True)
```

Append Series with calculated counts to grid (as new column) based on index match:

In [62]:

```
grid.loc[cardinality_series.index, 'postcount_est'] = cardinality_series
```

In [63]:

```
grid[grid["postcount_est"] > 0].head()
```

Out[63]:

|  |  | geometry | postcount\_est |
| --- | --- | --- | --- |
| xbin | ybin |  |  |
| -340096 | 5679952 | POLYGON ((-340096.000 5679952.000, -240096.000 5679952.000, -240096.000 5579952.000, -340096.000 5579952.000, -340096.000 5679952.000)) | 296 |
|  | 5179952 | POLYGON ((-340096.000 5179952.000, -240096.000 5179952.000, -240096.000 5079952.000, -340096.000 5079952.000, -340096.000 5179952.000)) | 634 |
|  | 5079952 | POLYGON ((-340096.000 5079952.000, -240096.000 5079952.000, -240096.000 4979952.000, -340096.000 4979952.000, -340096.000 5079952.000)) | 31 |
|  | 4979952 | POLYGON ((-340096.000 4979952.000, -240096.000 4979952.000, -240096.000 4879952.000, -340096.000 4879952.000, -340096.000 4979952.000)) | 7 |
|  | 4879952 | POLYGON ((-340096.000 4879952.000, -240096.000 4879952.000, -240096.000 4779952.000, -340096.000 4779952.000, -340096.000 4879952.000)) | 52 |

**Process all chunks:**

The caveat here is to incrementally union hll sets until all records have been processed. On the last loop, instruct the hll worker to return the cardinality instead of the unioned hll set.

First, define method to join cardinality to grid

In [64]:

```
# reference metric names and column names
COLUMN_METRIC_REF = {
        "postcount_est":"post_hll",
        "usercount_est":"user_hll",
        "userdays_est":"date_hll"}

def join_df_grid(
    df: pd.DataFrame, grid: gp.GeoDataFrame,
    metric: str = "postcount_est",
    cardinality: bool = True,
    column_metric_ref: Dict[str, str] = COLUMN_METRIC_REF):
    """Union HLL Sets and estimate postcount per 
    grid bin from lat/lng coordinates
    
        Args:
        df: A pandas dataframe with latitude and 
            longitude columns in WGS1984
        grid: A geopandas geodataframe with indexes 
            x and y (projected coordinates) and grid polys
        metric: target column for estimate aggregate.
            Default: postcount_est.
        cardinality: will compute cardinality of unioned
            hll sets. Otherwise, unioned hll sets will be 
            returned for incremental updates.
    """
    # optionally, bin assigment of projected coordinates,
    # make sure to not bin twice:
    # x/y columns are removed after binning
    if 'x' in df.columns:
        bin_coordinates(df, xbins, ybins)
        # set index column
        df.set_index(
            ['xbins_match', 'ybins_match'], inplace=True)
    # union hll sets and 
    # optional estimate count distincts (cardinality)
    column = column_metric_ref.get(metric)
    # get series with grouped hll sets
    hll_series = df[column]
    # union of hll sets:
    # to allow incremental union of already merged data
    # and new data, concatenate series from grid and new df
    # only if column with previous hll sets already exists
    if metric in grid.columns:
        # remove nan values from grid and
        # rename series to match names
        hll_series = pd.concat(
            [hll_series, grid[metric].dropna()]
            ).rename(column)
    cardinality_series = union_hll(
        hll_series, cardinality=cardinality)
    # add unioned hll sets/computed cardinality to grid
    grid.loc[
        cardinality_series.index, metric] = cardinality_series
    if cardinality:
        # set all remaining grid cells
        # with no data to zero and
        # downcast column type from float to int
        grid[metric] = grid[metric].fillna(0).astype(int)
```

Define method to process chunks:

In [65]:

```
def join_chunkeddf_grid(
    chunked_df: List[pd.DataFrame], grid: gp.GeoDataFrame,
    metric: str = "postcount_est", chunk_size: int = CHUNK_SIZE,
    keep_hll: Optional[bool] = None,
    column_metric_ref: Dict[str, str] = COLUMN_METRIC_REF):
    """Incremental union of HLL Sets and estimate postcount per 
    grid bin from chunked list of dataframe records. Results will
    be stored in grid.
    
    Args:
    chunked_df: A list of (chunked) dataframes with latitude and 
        longitude columns in WGS1984
    grid: A geopandas geodataframe with indexes 
        x and y (projected coordinates) and grid polys
    metric: target column for estimate aggregate.
        Default: postcount_est.
    keep_hll: If True, will not remove HLL sketches after
        final cardinality estimation. Will not reset metrics.
    """
    reset_metrics(grid, [metric])
    if keep_hll:
        # if existing hll sets present,
        # rename column so it can be updates
        metric_hll = metric.replace("_est", "_hll")
        if metric_hll in grid.columns:
            grid.rename(
                columns={
                    metric_hll: metric.replace(
                        "_hll", "_est")},
                    inplace=True)
    for ix, chunk_df in enumerate(chunked_df):
        # compute cardinality only on last iteration
        cardinality = False
        if ix == len(chunked_df)-1:
            cardinality = True
        column = column_metric_ref.get(metric)
        # get series with grouped hll sets
        hll_series = chunk_df[column]
        if metric in grid.columns:
            # merge existing hll sets with new ones
            # into one series (with duplicate indexes);
            # remove nan values from grid and
            # rename series to match names
            hll_series = pd.concat(
                [hll_series, grid[metric].dropna()]
                ).rename(column)
        cardinality_series = union_hll(
            hll_series, cardinality=cardinality)
        if keep_hll:
            # only if final hll sketches need to
            # be kept (e.g. for benchmarking):
            # do another union, without cardinality
            # estimation, and store results
            # in column "metric"_hll
            hll_sketch_series = union_hll(
                hll_series, cardinality=False)
            grid.loc[
                hll_sketch_series.index,
                f'{metric.replace("_est","_hll")}'] = hll_sketch_series
        # add unioned hll sets/computed cardinality to grid
        grid.loc[
            cardinality_series.index, metric] = cardinality_series
        if cardinality:
            # set all remaining grid cells
            # with no data to zero and
            # downcast column type from float to int
            grid[metric] = grid[metric].fillna(0).astype(int)
        clear_output(wait=True)
        print(f'Mapped ~{(ix+1)*chunk_size} coordinates to bins')
```

Execute:

In [66]:

```
join_chunkeddf_grid(chunked_df, grid, chunk_size=CHUNK_SIZE)
```

```
Mapped ~5000000 coordinates to bins
```

All distinct counts are now attached to the bins of the grid:

In [67]:

```
grid[grid["postcount_est"]>10].head()
```

Out[67]:

|  |  | geometry | postcount\_est |
| --- | --- | --- | --- |
| xbin | ybin |  |  |
| -340096 | 5679952 | POLYGON ((-340096.000 5679952.000, -240096.000 5679952.000, -240096.000 5579952.000, -340096.000 5579952.000, -340096.000 5679952.000)) | 296 |
|  | 5179952 | POLYGON ((-340096.000 5179952.000, -240096.000 5179952.000, -240096.000 5079952.000, -340096.000 5079952.000, -340096.000 5179952.000)) | 634 |
|  | 5079952 | POLYGON ((-340096.000 5079952.000, -240096.000 5079952.000, -240096.000 4979952.000, -340096.000 4979952.000, -340096.000 5079952.000)) | 31 |
|  | 4879952 | POLYGON ((-340096.000 4879952.000, -240096.000 4879952.000, -240096.000 4779952.000, -340096.000 4779952.000, -340096.000 4879952.000)) | 52 |
|  | 4579952 | POLYGON ((-340096.000 4579952.000, -240096.000 4579952.000, -240096.000 4479952.000, -340096.000 4479952.000, -340096.000 4579952.000)) | 58 |

### Preview post count map¶

Use headtail\_breaks classification scheme because it is specifically suited to map long tailed data, see Jiang 2013

- Jiang, B. (August 01, 2013). Head/Tail Breaks: A New Classification Scheme for Data with a Heavy-Tailed Distribution. The Professional Geographer, 65, 3, 482-494.

In [68]:

```
# global legend font size setting
plt.rc('legend', **{'fontsize': 16})
```

In [69]:

```
def leg_format(leg):
    "Format matplotlib legend entries"
    for lbl in leg.get_texts():
        label_text = lbl.get_text()
        lower = label_text.split(",")[0].lstrip("[(")
        upper = label_text.split(",")[1].rstrip(")]")
        new_text = f'{float(lower):,.0f} - {float(upper):,.0f}'
        lbl.set_text(new_text)
        
def title_mod(title, km_size: Optional[float] = km_size):
    """Update title/output name if grid size is not 100km"""
    if not km_size or km_size == 100:
        return title
    return f'{title} ({km_size:.0f} km grid)'

def save_plot(
    grid: gp.GeoDataFrame, title: str, column: str, save_fig: str = None,
    output: Path = OUTPUT):
    """Plot GeoDataFrame with matplotlib backend, optionaly export as png"""
    fig, ax = plt.subplots(1, 1,figsize=(10,12))
    ax.set_xlim(minx-buf, maxx+buf)
    ax.set_ylim(miny-buf, maxy+buf)
    title = title_mod(title)
    ax.set_title(title, fontsize=20)
    base = grid.plot(
        ax=ax, column=column, cmap='OrRd', scheme='headtail_breaks', 
        legend=True, legend_kwds={'loc': 'lower right'})
    # combine with world geometry
    plot = world.plot(
        ax=base, color='none', edgecolor='black', linewidth=0.1)
    leg = ax.get_legend()
    leg_format(leg)
    if not save_fig:
        return
    fig.savefig(output / f"figures{km_size_str}" / save_fig, dpi=300, format='PNG',
                bbox_inches='tight', pad_inches=1)
```

In [70]:

```
save_plot(
    grid=grid, title='Estimated Post Count',
    column='postcount_est', save_fig=f'postcount_sample_est.png')
```

**Note:** the data anomaly seen in Germany is located in the grid cell for Berlin. It is related to a single user who uploaded 53961 photos with sunset and sunrise. This anomaly will disappear once aggregation is based on user\_guid count.

## B: Estimated User Count per grid¶

When using HLL, aggregation of user\_guids or user\_days takes the same amount of time (unlike when working with original data, where memory consumption increases significantly). We'll only need to update the columns that are loaded from the database:

In [71]:

```
usecols = ['latitude', 'longitude', 'user_hll']
```

Adjust method for stream-reading from CSV in chunks:

In [72]:

```
iter_csv = pd.read_csv(
    SUNSET_FLICKR, usecols=usecols, iterator=True,
    dtype=dtypes, encoding='utf-8', chunksize=CHUNK_SIZE)
```

In [73]:

```
def proj_report(df, cnt, inplace: bool = False):
    """Project df with progress report"""
    proj_df(df)
    clear_output(wait=True)
    print(f'Projected {cnt:,.0f} coordinates')
    if inplace:
        return
    return df
```

In [74]:

```
%%time
# filter
chunked_df = [
    filter_df_bbox( 
        df=chunk_df, bbox=bbox_italy_buf, inplace=False)
    for chunk_df in iter_csv]

# project
projected_cnt = 0
for chunk_df in chunked_df:
    projected_cnt += len(chunk_df)
    proj_report(
        chunk_df, projected_cnt, inplace=True)

display(chunked_df[0].head())
```

```
Projected 2,119 coordinates
```

|  | user\_hll | x | y |
| --- | --- | --- | --- |
| 0 | \x138b4000810181026503a40423048207a607e10a030ac20ce40d010fc11063108111011162130114a21541166516a1172117e11b811ba31bc11c041c811d6220a220c7216121e222... | -265096.0 | 5604952.0 |
| 1 | \x138b4011a4bd82 | -265096.0 | 5554952.0 |
| 2 | \x138b407407 | -265096.0 | 5304952.0 |
| 3 | \x138b40a1c1 | -265096.0 | 5254952.0 |
| 4 | \x138b40fb45 | -265096.0 | 5204952.0 |

```
CPU times: user 71.1 ms, sys: 0 ns, total: 71.1 ms
Wall time: 70.4 ms
```

### Perform the bin assignment and estimate distinct users¶

In [75]:

```
def bin_coordinates(
        df: pd.DataFrame, xbins:
        np.ndarray, ybins: np.ndarray) -> pd.DataFrame:
    """Bin coordinates using binary search and append to df as new index"""
    xbins_match, ybins_match = get_best_bins(
        search_values_x=df['x'].to_numpy(),
        search_values_y=df['y'].to_numpy(),
        xbins=xbins, ybins=ybins)
    # append target bins to original dataframe
    # use .loc to avoid chained indexing
    df.loc[:, 'xbins_match'] = xbins_match
    df.loc[:, 'ybins_match'] = ybins_match
    # drop x and y columns not needed anymore
    df.drop(columns=['x', 'y'], inplace=True)
```

In [76]:

```
def bin_chunked_coordinates(
    chunked_df: List[pd.DataFrame]):
    """Bin coordinates of chunked dataframe"""
    binned_cnt = 0
    for ix, df in enumerate(chunked_df):
        bin_coordinates(df, xbins, ybins)
        df.set_index(['xbins_match', 'ybins_match'], inplace=True)
        clear_output(wait=True)
        binned_cnt += len(df)
        print(f"Binned {binned_cnt:,.0f} coordinates..")
```

In [77]:

```
%%time
bin_chunked_coordinates(chunked_df)
display(chunked_df[0].head())
```

```
Binned 2,119 coordinates..
```

|  |  | user\_hll |
| --- | --- | --- |
| xbins\_match | ybins\_match |  |
| -340096 | 5679952 | \x138b4000810181026503a40423048207a607e10a030ac20ce40d010fc11063108111011162130114a21541166516a1172117e11b811ba31bc11c041c811d6220a220c7216121e222... |
|  | 5579952 | \x138b4011a4bd82 |
|  | 5379952 | \x138b407407 |
|  | 5279952 | \x138b40a1c1 |
|  | 5279952 | \x138b40fb45 |

```
CPU times: user 8.84 ms, sys: 0 ns, total: 8.84 ms
Wall time: 8.23 ms
```

Union HLL Sets per grid-id and calculate cardinality (estimated distinct user count):

In [78]:

```
join_chunkeddf_grid(
    chunked_df=chunked_df, grid=grid, metric="usercount_est")
```

```
Mapped ~5000000 coordinates to bins
```

In [79]:

```
grid[grid["usercount_est"]> 0].head()
```

Out[79]:

|  |  | geometry | postcount\_est | usercount\_est |
| --- | --- | --- | --- | --- |
| xbin | ybin |  |  |  |
| -340096 | 5679952 | POLYGON ((-340096.000 5679952.000, -240096.000 5679952.000, -240096.000 5579952.000, -340096.000 5579952.000, -340096.000 5679952.000)) | 296 | 290 |
|  | 5579952 | POLYGON ((-340096.000 5579952.000, -240096.000 5579952.000, -240096.000 5479952.000, -340096.000 5479952.000, -340096.000 5579952.000)) | 0 | 2 |
|  | 5379952 | POLYGON ((-340096.000 5379952.000, -240096.000 5379952.000, -240096.000 5279952.000, -340096.000 5279952.000, -340096.000 5379952.000)) | 0 | 1 |
|  | 5279952 | POLYGON ((-340096.000 5279952.000, -240096.000 5279952.000, -240096.000 5179952.000, -340096.000 5179952.000, -340096.000 5279952.000)) | 0 | 2 |
|  | 5179952 | POLYGON ((-340096.000 5179952.000, -240096.000 5179952.000, -240096.000 5079952.000, -340096.000 5079952.000, -340096.000 5179952.000)) | 634 | 520 |

Look at this. There're many polygons were thounsands of photos have been created by only few users. Lets see how this affects our test map..

### Preview user count map¶

In [80]:

```
save_plot(
    grid=grid, title='Estimated User Count',
    column='usercount_est', save_fig='usercount_sample_est.png')
```

## C: Estimated User Days¶

Usually, due to the Count Distinct Problem increasing computation times will apply for more complex distinct queries. This is not the case when using HLL. Any count distinct (postcount, usercount etc.) requires the same amount of time. A useful metric introduced by Wood et al. (2013) is User Days, which lies inbetween Post Count and User Count because Users may be counted more than once if they visited the location on consecutive days. User Days particularly allows capturing the difference between local and tourist behaviour patterns. The rationale here is that locals visit few places very often. In contrast, tourists visit many places only once.

The sequence of commands for userdays is exactly the same as for postcount and usercount above.

In [81]:

```
usecols = ['latitude', 'longitude', 'date_hll']
```

In [82]:

```
def read_project_chunked(filename: str,
    usecols: List[str], chunk_size: int = CHUNK_SIZE,
    bbox: Tuple[float, float, float, float] = None) -> List[pd.DataFrame]:
    """Read data from csv, optionally clip to bbox and projet"""
    iter_csv = pd.read_csv(
        filename, usecols=usecols, iterator=True,
        dtype=dtypes, encoding='utf-8', chunksize=chunk_size)
    if bbox:
        chunked_df = [filter_df_bbox( 
            df=chunk_df, bbox=bbox, inplace=False)
        for chunk_df in iter_csv]
    else:
        chunked_df = [chunk_df for chunk_df in iter_csv]
    # project
    projected_cnt = 0
    for chunk_df in chunked_df:
        projected_cnt += len(chunk_df)
        proj_report(
            chunk_df, projected_cnt, inplace=True)
    return chunked_df
```

Run:

In [83]:

```
%%time
chunked_df = read_project_chunked(
    filename=SUNRISE_FLICKR,
    usecols=usecols,
    bbox=bbox_italy_buf)
display(chunked_df[0].head())
```

```
Projected 1,716 coordinates
```

|  | date\_hll | x | y |
| --- | --- | --- | --- |
| 0 | \x138b7f0001008100c10181020102c203c4042204c107420823096109c40aa10b030c010cc10da10ec510e111021141118412811423148215211602176117c219641a011a611ac21c... | -265096.0 | 5604952.0 |
| 1 | \x138b7f006100a1012201a101e1020202a2038203c404030421044404e205810602066207250742088108a508c109e40a420a650aa20b010be10c010c820cc20ce50d430d810e410e... | -265096.0 | 5154952.0 |
| 2 | \x128b7f87395d0bf77a98139433f92a09030800afb394eff42a4ac5b6586780db4caa87c3e8fdf4182f7016e05b347ce903311ae8e60e69e784399de936f5737b8351e5074c8ec884... | -265096.0 | 5104952.0 |
| 3 | \x128b7f8db3093bc0ed745a928779dfdf2009e8955a4c1125c6920f9e67830dcec650afa7c9044472810eceb5f57170b0035e88b90ac9b325921f87c4f14b37c6fb2cacc6b215a798... | -265096.0 | 5054952.0 |
| 4 | \x128b7fc5197a970c606ea1279e1f9e9890370932bc0618692118c9 | -265096.0 | 4954952.0 |

```
CPU times: user 79.3 ms, sys: 3.94 ms, total: 83.2 ms
Wall time: 82.4 ms
```

In [84]:

```
%%time
bin_chunked_coordinates(chunked_df)
```

```
Binned 1,716 coordinates..
CPU times: user 5.02 ms, sys: 72 µs, total: 5.1 ms
Wall time: 4.64 ms
```

In [85]:

```
join_chunkeddf_grid(
    chunked_df=chunked_df, grid=grid, metric="userdays_est")
```

```
Mapped ~5000000 coordinates to bins
```

In [86]:

```
grid[grid["userdays_est"]> 0].head()
```

Out[86]:

|  |  | geometry | postcount\_est | usercount\_est | userdays\_est |
| --- | --- | --- | --- | --- | --- |
| xbin | ybin |  |  |  |  |
| -340096 | 5679952 | POLYGON ((-340096.000 5679952.000, -240096.000 5679952.000, -240096.000 5579952.000, -340096.000 5579952.000, -340096.000 5679952.000)) | 296 | 290 | 290 |
|  | 5179952 | POLYGON ((-340096.000 5179952.000, -240096.000 5179952.000, -240096.000 5079952.000, -340096.000 5079952.000, -340096.000 5179952.000)) | 634 | 520 | 620 |
|  | 5079952 | POLYGON ((-340096.000 5079952.000, -240096.000 5079952.000, -240096.000 4979952.000, -340096.000 4979952.000, -340096.000 5079952.000)) | 31 | 36 | 31 |
|  | 4979952 | POLYGON ((-340096.000 4979952.000, -240096.000 4979952.000, -240096.000 4879952.000, -340096.000 4879952.000, -340096.000 4979952.000)) | 7 | 48 | 7 |
|  | 4879952 | POLYGON ((-340096.000 4879952.000, -240096.000 4879952.000, -240096.000 4779952.000, -340096.000 4779952.000, -340096.000 4879952.000)) | 52 | 108 | 51 |

Note below that our very active Berlin user doesn't affect the map as strong as with postcount. Of all three metrics, userdays per grid perhaps provides the most balanced capture of behaviour patterns.

In [87]:

```
save_plot(
    grid=grid, title='Estimated User Days',
    column='userdays_est', save_fig='userdays_sample_est.png')
```

There're other approaches for further reducing noise. For example, to reduce the impact of automatic capturing devices (such as webcams uploading x pictures per day), a possibility is to count distinct **userlocations**. For userlocations metric, a user would be counted multiple times per grid bin only for pictures with different lat/lng. Or the number of distinct **userlocationdays** (etc.). These metrics easy to implement using hll, but would be quite difficult to compute using raw data.

# Prepare methods¶

Lets summarize the above code in a few methods:

**Plotting preparation**

The below methods contain combined code from above, plus final plot style improvements.

In [88]:

```
def format_legend(
    leg, bounds: List[str], inverse: bool = None,
    metric: str = "postcount_est"):
    """Formats legend (numbers rounded, colors etc.)"""
    leg.set_bbox_to_anchor((0., 0.3, 0.2, 0.3))
    # get all the legend labels
    legend_labels = leg.get_texts()
    plt.setp(legend_labels, fontsize='12')
    lcolor = 'black'
    if inverse:
        frame = leg.get_frame()
        frame.set_facecolor('black')
        frame.set_edgecolor('grey')
        lcolor = "white"
    plt.setp(legend_labels, color = lcolor)
    if metric == "postcount_est":
        leg.set_title("Estimated Post Count")
    elif metric == "usercount_est":
        leg.set_title("Estimated User Count")
    else:
        leg.set_title("Estimated User Days")
    plt.setp(leg.get_title(), fontsize='12')
    leg.get_title().set_color(lcolor)
    # replace the numerical legend labels
    for bound, legend_label in zip(bounds, legend_labels):
        legend_label.set_text(bound)
```

Label plotting

In [89]:

```
def _rnd_f(f: float, dec: int = None) -> str:
    if dec is None:
        # return f'{f}'
        dec = 0
    return f'{f:,.{dec}f}'

def format_bound(
    upper_bound: float = None, lower_bound: float = None, 
    decimals: Optional[int] = None) -> str:
    """Format legend text for class bounds"""
    if upper_bound is None:
        return _rnd_f(lower_bound, decimals)
    if lower_bound is None:
        return _rnd_f(upper_bound, decimals)
    return f'{_rnd_f(lower_bound, decimals)} - {_rnd_f(upper_bound, decimals)}'

def min_decimals(num1: float, num2: float) -> int:
    """Return number of minimum required decimals"""
    if _rnd_f(num1) != _rnd_f(num2):
        return 0
    for i in range(1, 5):
        if _rnd_f(num1, i) != _rnd_f(num2, i):
            return i
    return 5
        
def get_label_bounds(
    scheme_classes: np.ndarray, metric_series: pd.Series,
    flat: bool = None) -> List[str]:
    """Get all upper bounds in the scheme_classes category"""
    upper_bounds = scheme_classes
    # get and format all bounds
    bounds = []
    for idx, upper_bound in enumerate(upper_bounds):
        if idx == 0:
            lower_bound = metric_series.min()
            decimals = None
        else:
            decimals = min_decimals(
                upper_bounds[idx-1], upper_bounds[idx])
            lower_bound = upper_bounds[idx-1]
        if flat:
            bound = format_bound(
                lower_bound=lower_bound,
                decimals=decimals)
        else:
            bound = format_bound(
                upper_bound, lower_bound,
                decimals=decimals)
        bounds.append(bound)
    if flat:
        upper_bound = format_bound(
            upper_bound=upper_bounds[-1],
            decimals=decimals)
        bounds.append(upper_bound)
    return bounds
```

Classify & plotting

In [90]:

```
def label_nodata(
    grid: gp.GeoDataFrame, inverse: bool = None,
    metric: str = "postcount_est"):
    """Add white to a colormap to represent missing value
    
    Adapted from:
        https://stackoverflow.com/a/58160985/4556479
        
    See available colormaps:
        http://holoviews.org/user_guide/Colormaps.html
    """
    # set 0 to NaN
    grid_nan = grid[metric].replace(0, np.nan)
    # get headtail_breaks
    # excluding NaN values
    headtail_breaks = mc.HeadTailBreaks(
        grid_nan.dropna())
    grid[f'{metric}_cat'] = headtail_breaks.find_bin(
        grid_nan).astype('str')
    # set label for NaN values
    grid.loc[grid_nan.isnull(), f'{metric}_cat'] = 'No Data'
    bounds = get_label_bounds(
        headtail_breaks.bins, grid_nan.dropna().values)
    cmap_name = 'OrRd'
    nodata_color = 'white'
    if inverse:
        nodata_color = 'black'
        cmap_name = 'cet_fire'
    cmap = plt.cm.get_cmap(cmap_name, headtail_breaks.k)
    # get hex values
    cmap_list = [colors.rgb2hex(cmap(i)) for i in range(cmap.N)]
    # lighten or darken up first/last color a bit 
    # to offset from black or white background
    if inverse:
        firstcolor = '#3E0100'
        cmap_list[0] = firstcolor
    else:
        lastcolor = '#440402'
        cmap_list.append(lastcolor)
        cmap_list.pop(0)
    # append nodata color
    cmap_list.append(nodata_color)
    cmap_with_nodata = colors.ListedColormap(cmap_list)
    return cmap_with_nodata, bounds

def plot_figure(
    grid: gp.GeoDataFrame, title: str, inverse: bool = None,
    metric: str = "postcount_est", store_fig: str = None,
    output: Path = OUTPUT):
    """Combine layers and plot"""
    # for plotting, there're some minor changes applied
    # to the dataframe (replace NaN values),
    # make a shallow copy here to prevent changes
    # to modify the original grid
    grid_plot = grid.copy()
    # create new plot figure object with one axis
    fig, ax = plt.subplots(1, 1, figsize=(22,28))
    ax.set_title(title, fontsize=16)
    print("Classifying bins..")
    cmap_with_nodata, bounds = label_nodata(
        grid=grid_plot, inverse=inverse, metric=metric)
    base = grid_plot.plot(
        ax=ax,
        column=f'{metric}_cat', cmap=cmap_with_nodata, legend=True)
    print("Formatting legend..")
    leg = ax.get_legend()
    format_legend(leg, bounds, inverse, metric)
    # combine with world geometry
    edgecolor = 'black'
    ax.set_facecolor('white')
    if inverse:
        edgecolor = 'white'
        ax.set_facecolor('black')
    plot = world.plot(
        ax=base, color='none', edgecolor=edgecolor, linewidth=0.1)
    if store_fig:
        print("Storing figure as png..")
        filename = f'{store_fig}.png'
        if inverse:
            filename = f'{store_fig}_inverse.png'
        fig.savefig(
            output / f"figures{km_size_str}" / filename, dpi=300, format='PNG',
            bbox_inches='tight', pad_inches=1)
```

Add specific method to filter Null Island from dataframe

In [91]:

```
def filter_nullisland_df(
    df: Optional[pd.DataFrame] = None, df_list: Optional[List[pd.DataFrame]] = None,
    col_x: str = "longitude", col_y: str = "latitude"):
    """Remove records from df inplace where both x and y coordinate are 0"""
    if df is not None:
        df_list = [df]
    if not df_list:
        raise ValueError("Please provide either df or df_list")
    for df in df_list:
        if col_x in df.columns:
            df.query(
                f'({col_x} == 0 and {col_y} == 0) == False',
                inplace=True)
```

High level plotting function

In [92]:

```
def load_plot(
    data: Path, grid: gp.GeoDataFrame, title: Optional[str] = "", inverse: Optional[bool] = None,
    filter_null_island: bool = None, 
    metric: str = "postcount_est", filename: str = None,
    chunk_size: int = CHUNK_SIZE, keep_hll: Optional[bool] = None,
    store_benchmark_data: Optional[bool] = None,
    data_list: List[Path] = None, plot: Optional[bool] = True,
    column_metric_ref: Dict[str, str] = COLUMN_METRIC_REF,
    reset_benchmark_data: Optional[bool] = None):
    """Load data, bin coordinates, estimate distinct counts (cardinality) and plot map
    
        Args:
        data: Path to read input CSV
        grid: A geopandas geodataframe with indexes x and y 
            (projected coordinates) and grid polys
        title: Title of the plot
        inverse: If True, inverse colors (black instead of white map)
        metric: target column for aggregate. Default: postcount_est.
        filename: Provide a name to store figure as PNG. Will append 
            '_inverse.png' if inverse=True.
        chunk_size: chunk processing into x records per chunk
        keep_hll: If True, hll sets will not be removed 
            after final estimation of cardinality
        store_benchmark_data: If True, hll data will be stored to separate
            output pickle file, ending with _hll.pkl
        data_list: Optionally provide a list of data paths that will be combined.
        plot: Plot & store figure (Default = True).
        reset_benchmark_data: Optionally remove/reset hll data from previous run.
    """
    if store_benchmark_data is None:
        benchmark_data = False
    if reset_benchmark_data is None:
        reset_benchmark_data = True
    if store_benchmark_data:
        keep_hll = True
    if inverse is None:
        inverse = False
    if filter_null_island is None:
        filter_null_island = True
    if reset_benchmark_data:
        hll_col = metric.replace("_est", "_hll")
        grid.drop(columns=[hll_col], inplace=True, errors='ignore')
    usecols = ['latitude', 'longitude']
    column = column_metric_ref.get(metric)
    usecols.append(column)
    # get data from csv
    chunked_df = read_project_chunked(
        filename=data,
        usecols=usecols)
     # optionally filter null island
    if filter_null_island:
        filter_nullisland_df(df_list=chunked_df)
    # bin coordinates
    bin_chunked_coordinates(chunked_df)
    # reset metric column
    reset_metrics(grid, [metric], setzero=False)
    print("Getting cardinality per bin..")
    # union hll sets per chunk and 
    # calculate distinct counts on last iteration
    join_chunkeddf_grid(
        chunked_df=chunked_df, grid=grid,
        metric=metric, chunk_size=chunk_size,
        keep_hll=keep_hll)
    # store intermediate data
    if store_benchmark_data:
        print("Storing aggregate data as pickle..")
        grid.to_pickle(OUTPUT / f"pickles{km_size_str}" / f"{filename}.pkl")
        print("Storing benchmark hll data as pickle..")
        grid[[metric.replace("_est", "_hll"), "geometry"]].to_pickle(
            OUTPUT / f"pickles{km_size_str}" / f"{filename}_hll.pkl")
        print("Storing benchmark hll data as csv..")
        tools.export_benchmark(
            grid=grid, filename=filename,
            metric=metric, km_size_str=km_size_str, output=OUTPUT,
            column_metric_ref=column_metric_ref,
            proj_transformer_back=PROJ_TRANSFORMER_BACK)
    if plot:
        print("Plotting figure..")
        plot_figure(grid, title, inverse, metric, filename)
```

# Plotting worldmaps: Post Count¶

Plot worldmap for each datasource

In [93]:

```
reset_metrics(grid)
```

In [94]:

```
%%time
load_plot(
    SUNSET_FLICKR, grid, title=f'Estimated "Sunset" Post Count (Flickr) per {km_size:.0f} km grid, 2007-2018',
    filename="flickr_postcount_sunset_est", store_benchmark_data=True)
```

```
Mapped ~5000000 coordinates to bins
Storing aggregate data as pickle..
Storing benchmark hll data as pickle..
Storing benchmark hll data as csv..
Plotting figure..
Classifying bins..
Formatting legend..
Storing figure as png..
CPU times: user 16.3 s, sys: 468 ms, total: 16.7 s
Wall time: 16.9 s
```

In [95]:

```
%%time
reset_metrics(grid)
load_plot(
    SUNRISE_FLICKR, grid, title=f'Estimated "Sunrise" Post Count (Flickr) per {km_size:.0f} km grid, 2007-2018',
    filename="flickr_postcount_sunrise_est", store_benchmark_data=True)
```

```
Mapped ~5000000 coordinates to bins
Storing aggregate data as pickle..
Storing benchmark hll data as pickle..
Storing benchmark hll data as csv..
Plotting figure..
Classifying bins..
Formatting legend..
Storing figure as png..
CPU times: user 14.5 s, sys: 379 ms, total: 14.9 s
Wall time: 14.8 s
```

In [96]:

```
%%time
reset_metrics(grid)
load_plot(
    SUNSET_INSTAGRAM, grid, title=f'Estimated "Sunset" Post Count (Instagram) per {km_size:.0f} km grid, Aug-Dec 2017',
    filename="instagram_postcount_sunset_est", store_benchmark_data=True)
```

```
Mapped ~5000000 coordinates to bins
Storing aggregate data as pickle..
Storing benchmark hll data as pickle..
Storing benchmark hll data as csv..
Plotting figure..
Classifying bins..
Formatting legend..
Storing figure as png..
CPU times: user 15.2 s, sys: 423 ms, total: 15.7 s
Wall time: 16.1 s
```

In [97]:

```
%%time
reset_metrics(grid)
load_plot(
    SUNRISE_INSTAGRAM, grid, title=f'Estimated "Sunrise" Post Count (Instagram) per {km_size:.0f} km grid, Aug-Dec 2017',
    filename="instagram_postcount_sunrise_est", store_benchmark_data=True)
```

```
Mapped ~5000000 coordinates to bins
Storing aggregate data as pickle..
Storing benchmark hll data as pickle..
Storing benchmark hll data as csv..
Plotting figure..
Classifying bins..
Formatting legend..
Storing figure as png..
CPU times: user 14.8 s, sys: 436 ms, total: 15.2 s
Wall time: 15.3 s
```

In [98]:

```
%%time
reset_metrics(grid)
load_plot(
    ALL_FLICKR, grid, title=f'Estimated Post Count (Flickr total) per {km_size:.0f} km grid, 2007-2018',
    filename="flickr_postcount_all_est", store_benchmark_data=True)
```

```
Mapped ~5000000 coordinates to bins
Storing aggregate data as pickle..
Storing benchmark hll data as pickle..
Storing benchmark hll data as csv..
Plotting figure..
Classifying bins..
Formatting legend..
Storing figure as png..
CPU times: user 18.3 s, sys: 700 ms, total: 19 s
Wall time: 21.1 s
```

In [99]:

```
%%time
reset_metrics(grid)
load_plot(
    RANDOM_INSTAGRAM, grid, title=f'Estimated Post Count (Instagram Random 20M) per {km_size:.0f} km grid',
    filename="instagram_postcount_random_est", store_benchmark_data=True)
```

```
Mapped ~5000000 coordinates to bins
Storing aggregate data as pickle..
Storing benchmark hll data as pickle..
Storing benchmark hll data as csv..
Plotting figure..
Classifying bins..
Formatting legend..
Storing figure as png..
CPU times: user 15.9 s, sys: 506 ms, total: 16.4 s
Wall time: 16.9 s
```

# Plotting worldmaps: User Count¶

In addition to the cardinality, for user count and user days, the hll sets itself are stored (`store_benchmark_data=True`)

In [100]:

```
%%time
reset_metrics(grid)
load_plot(
    SUNSET_FLICKR, grid, title=f'Estimated "Sunset" User Count (Flickr) per {km_size:.0f} km grid, 2007-2018',
    filename="flickr_usercount_sunset_est", metric="usercount_est", store_benchmark_data=True)
```

```
Mapped ~5000000 coordinates to bins
Storing aggregate data as pickle..
Storing benchmark hll data as pickle..
Storing benchmark hll data as csv..
Plotting figure..
Classifying bins..
Formatting legend..
Storing figure as png..
CPU times: user 14.9 s, sys: 348 ms, total: 15.3 s
Wall time: 15.3 s
```

In [101]:

```
%%time
reset_metrics(grid)
load_plot(
    SUNRISE_FLICKR, grid, title=f'Estimated "Sunrise" User Count (Flickr) per {km_size:.0f} km grid, 2007-2018',
    filename="flickr_usercount_sunrise_est", metric="usercount_est", store_benchmark_data=True)
```

```
Mapped ~5000000 coordinates to bins
Storing aggregate data as pickle..
Storing benchmark hll data as pickle..
Storing benchmark hll data as csv..
Plotting figure..
Classifying bins..
Formatting legend..
Storing figure as png..
CPU times: user 14.2 s, sys: 359 ms, total: 14.6 s
Wall time: 14.4 s
```

In [102]:

```
%%time
reset_metrics(grid)
load_plot(
    SUNSET_INSTAGRAM, grid, title=f'Estimated "Sunset" User Count (Instagram) per {km_size:.0f} km grid, Aug-Dec 2017',
    filename="instagram_usercount_sunset_est", metric="usercount_est", store_benchmark_data=True)
```

```
Mapped ~5000000 coordinates to bins
Storing aggregate data as pickle..
Storing benchmark hll data as pickle..
Storing benchmark hll data as csv..
Plotting figure..
Classifying bins..
Formatting legend..
Storing figure as png..
CPU times: user 15.2 s, sys: 388 ms, total: 15.6 s
Wall time: 16 s
```

In [103]:

```
%%time
reset_metrics(grid)
load_plot(
    SUNRISE_INSTAGRAM, grid, title=f'Estimated "Sunrise" User Count (Instagram) per {km_size:.0f} km grid, Aug-Dec 2017',
    filename="instagram_usercount_sunrise_est", metric="usercount_est", store_benchmark_data=True)
```

```
Mapped ~5000000 coordinates to bins
Storing aggregate data as pickle..
Storing benchmark hll data as pickle..
Storing benchmark hll data as csv..
Plotting figure..
Classifying bins..
Formatting legend..
Storing figure as png..
CPU times: user 14.6 s, sys: 350 ms, total: 14.9 s
Wall time: 15.1 s
```

In [104]:

```
%%time
reset_metrics(grid)
load_plot(
    ALL_FLICKR, grid, f'Flickr all User Counts (estimated) per {km_size:.0f} km grid, 2007-2018',
    filename="flickr_usercount_all_est", metric="usercount_est", store_benchmark_data=True)
```

```
Mapped ~5000000 coordinates to bins
Storing aggregate data as pickle..
Storing benchmark hll data as pickle..
Storing benchmark hll data as csv..
Plotting figure..
Classifying bins..
Formatting legend..
Storing figure as png..
CPU times: user 18.7 s, sys: 465 ms, total: 19.1 s
Wall time: 20.6 s
```

In [105]:

```
%%time
reset_metrics(grid)
load_plot(
    RANDOM_INSTAGRAM, grid, title=f'Estimated User Count (Instagram Random 20M) per {km_size:.0f} km grid',
    filename="instagram_usercount_random_est", metric="usercount_est", store_benchmark_data=True)
```

```
Mapped ~5000000 coordinates to bins
Storing aggregate data as pickle..
Storing benchmark hll data as pickle..
Storing benchmark hll data as csv..
Plotting figure..
Classifying bins..
Formatting legend..
Storing figure as png..
CPU times: user 16.3 s, sys: 508 ms, total: 16.8 s
Wall time: 17.2 s
```

# Plotting worldmaps: User Days¶

For usercount, we'll also store the hll sets, to be able to later continue to aggregate data (country summaries).

In [106]:

```
%%time
reset_metrics(grid)
load_plot(
    SUNSET_FLICKR, grid, title=f'Estimated "Sunset" User Days (Flickr) per {km_size:.0f} km grid, 2007-2018',
    filename="flickr_userdays_sunset_est", metric="userdays_est", store_benchmark_data=True)
```

```
Mapped ~5000000 coordinates to bins
Storing aggregate data as pickle..
Storing benchmark hll data as pickle..
Storing benchmark hll data as csv..
Plotting figure..
Classifying bins..
Formatting legend..
Storing figure as png..
CPU times: user 15.7 s, sys: 355 ms, total: 16 s
Wall time: 16.2 s
```

In [107]:

```
%%time
reset_metrics(grid)
load_plot(
    SUNRISE_FLICKR, grid, title=f'Estimated "Sunrise" User Days (Flickr) per {km_size:.0f} km grid, 2007-2018',
    filename="flickr_userdays_sunrise_est", metric="userdays_est", store_benchmark_data=True)
```

```
Mapped ~5000000 coordinates to bins
Storing aggregate data as pickle..
Storing benchmark hll data as pickle..
Storing benchmark hll data as csv..
Plotting figure..
Classifying bins..
Formatting legend..
Storing figure as png..
CPU times: user 14.8 s, sys: 372 ms, total: 15.2 s
Wall time: 15 s
```

In [108]:

```
%%time
reset_metrics(grid)
load_plot(
    SUNSET_INSTAGRAM, grid, title=f'Estimated "Sunset" User Days (Instagram) per {km_size:.0f} km grid, Aug-Dec 2017',
    filename="instagram_userdays_sunset_est", metric="userdays_est", store_benchmark_data=True)
```

```
Mapped ~5000000 coordinates to bins
Storing aggregate data as pickle..
Storing benchmark hll data as pickle..
Storing benchmark hll data as csv..
Plotting figure..
Classifying bins..
Formatting legend..
Storing figure as png..
CPU times: user 15.1 s, sys: 404 ms, total: 15.5 s
Wall time: 15.9 s
```

In [109]:

```
%%time
reset_metrics(grid)
load_plot(
    SUNRISE_INSTAGRAM, grid, title=f'Estimated "Sunrise" User Days (Instagram) per {km_size:.0f} km grid, Aug-Dec 2017',
    filename="instagram_userdays_sunrise_est", metric="userdays_est", store_benchmark_data=True)
```

```
Mapped ~5000000 coordinates to bins
Storing aggregate data as pickle..
Storing benchmark hll data as pickle..
Storing benchmark hll data as csv..
Plotting figure..
Classifying bins..
Formatting legend..
Storing figure as png..
CPU times: user 14.7 s, sys: 396 ms, total: 15.1 s
Wall time: 15.3 s
```

In [110]:

```
%%time
reset_metrics(grid)
load_plot(
    ALL_FLICKR, grid, title=f'Flickr all User Days (estimated) per {km_size:.0f} km grid, 2007-2018',
    filename="flickr_userdays_all_est", metric="userdays_est", store_benchmark_data=True)
```

```
Mapped ~5000000 coordinates to bins
Storing aggregate data as pickle..
Storing benchmark hll data as pickle..
Storing benchmark hll data as csv..
Plotting figure..
Classifying bins..
Formatting legend..
Storing figure as png..
CPU times: user 18.8 s, sys: 547 ms, total: 19.4 s
Wall time: 21.1 s
```

In [111]:

```
%%time
reset_metrics(grid)
load_plot(
    RANDOM_INSTAGRAM, grid, title=f'Instagram User Days (estimated, Random 20M) per {km_size:.0f} km grid',
    filename="instagram_userdays_random_est", metric="userdays_est", store_benchmark_data=True)
```

```
Mapped ~5000000 coordinates to bins
Storing aggregate data as pickle..
Storing benchmark hll data as pickle..
Storing benchmark hll data as csv..
Plotting figure..
Classifying bins..
Formatting legend..
Storing figure as png..
CPU times: user 15.4 s, sys: 439 ms, total: 15.8 s
Wall time: 16.4 s
```

## Merging Instagram Sunset and Sunrise data¶

For the Instagram chi calculation, we need a dataset with total number of users per bin. Given the volume of total number of photos shared on Instagram, this is quite difficult (~50 billion pictures shared to-date). As an alternative, we can combine both sunrise and sunset posts. Use the `load_plot()` function with `keep_hll` and create the plot only on second run.

In [112]:

```
def merge_instagram_sunsetsunrise(
        grid: gp.GeoDataFrame, metric: str,
        sunrise_instagram=SUNRISE_INSTAGRAM, sunset_instagram=SUNSET_INSTAGRAM,
        km_size_str=km_size_str):
    """Merge Instagram Sunset & Sunrise data as a replacement for Expected (All), store pickles"""
    # reset grid metrics
    reset_metrics(grid)
    load_plot(
        sunrise_instagram, grid, metric=metric, plot=False, keep_hll=True)
    
    # aggregate usercount per bin and store pickle
    load_plot(
        sunset_instagram, grid,
        title=(f'Estimated "Sunrise+Sunset" {metric.replace("_est", "").capitalize()} '
               f'(Instagram) per {km_size:.0f} km grid, Aug-Dec 2017'),
        filename=f"instagram_{metric.replace('_est', '')}_sunsetsunrise_est",
        metric=metric, keep_hll=True, reset_benchmark_data=False)
    
    # store benchmark data
    grid[metric.replace("_est", "_hll")].to_frame().to_pickle(
        OUTPUT / f"pickles{km_size_str}" / f"instagram_{metric.replace('_est', '')}_sunsetsunrise_est_hll.pkl")
    
    # drop hll column
    grid.drop(columns=[metric.replace("_est", "_hll")], inplace=True)
    # save to pickle
    grid.to_pickle(
        OUTPUT / f"pickles{km_size_str}" / f"instagram_{metric.replace('_est', '')}_sunsetsunrise_est.pkl")
```

In [113]:

```
%%time
merge_instagram_sunsetsunrise(grid=grid, metric="usercount_est")
```

```
Mapped ~5000000 coordinates to bins
Plotting figure..
Classifying bins..
Formatting legend..
Storing figure as png..
CPU times: user 15.2 s, sys: 396 ms, total: 15.6 s
Wall time: 16.7 s
```

In [114]:

```
%%time
merge_instagram_sunsetsunrise(grid=grid, metric="userdays_est")
```

```
Mapped ~5000000 coordinates to bins
Plotting figure..
Classifying bins..
Formatting legend..
Storing figure as png..
CPU times: user 14.8 s, sys: 416 ms, total: 15.2 s
Wall time: 16.2 s
```

In [115]:

```
%%time
merge_instagram_sunsetsunrise(grid=grid, metric="postcount_est")
```

```
Mapped ~5000000 coordinates to bins
Plotting figure..
Classifying bins..
Formatting legend..
Storing figure as png..
CPU times: user 14.5 s, sys: 404 ms, total: 14.9 s
Wall time: 16 s
```

# Save & load intermediate data¶

## Load & plot pickled dataframe¶

Loading (geodataframe) using pickle. This is the easiest way to store intermediate data, but may be incompatible if package versions change. If loading pickles does not work, a workaround is to load data from CSV and re-create pickle data, which will be compatible with used versions.

**Load pickled dataframe:**

In [116]:

```
%%time
grid = pd.read_pickle(OUTPUT / f"pickles{km_size_str}" / "flickr_postcount_all_est.pkl")
```

```
CPU times: user 269 ms, sys: 12 ms, total: 281 ms
Wall time: 280 ms
```

Then use `plot_figure` on dataframe to plot with new parameters, e.g. plot inverse:

In [117]:

```
plot_figure(grid, "Pickle Test", inverse=True, metric="postcount_est")
```

```
Classifying bins..
Formatting legend..
```

**To merge results (e.g. postcount, usercount, userdays) of multiple pickles:**

In [118]:

```
pickles_path = OUTPUT / f"pickles{km_size_str}"
pickle_list_flickr = [
    pickles_path / "flickr_postcount_all_est.pkl",
    pickles_path / "flickr_usercount_all_est.pkl",
    pickles_path / "flickr_userdays_all_est.pkl"
]
pickle_list_instagram = [
    pickles_path / "instagram_postcount_random_est.pkl",
    pickles_path / "instagram_usercount_random_est.pkl",
    pickles_path / "instagram_userdays_random_est.pkl"
]
column_list = [
    "postcount_est",
    "usercount_est",
    "userdays_est"
]
```

In [119]:

```
def load_pickle_merge(pickle_list: List[Path], column_list: List[str]):
    """Load pickles and merge columns based on list"""
    grid_list = []
    for pickle_path in pickle_list:
        grid_list.append(pd.read_pickle(pickle_path))
    grid = grid_list[0]
    for ix in range(1, len(grid_list)):
        grid = grid.merge(
            grid_list[ix][[column_list[ix]]],
            left_index=True, right_index=True)
    return grid
```

In [120]:

```
grid = load_pickle_merge(pickle_list_flickr, column_list)
```

Have a look at the numbers for exact and estimated values. Smaller values are exact in both hll and raw because Sparse Mode is used.

In [121]:

```
grid[grid["usercount_est"]>5].drop(columns=["geometry"]).head()
```

Out[121]:

|  |  | postcount\_hll | postcount\_est | usercount\_est | userdays\_est |
| --- | --- | --- | --- | --- | --- |
| xbin | ybin |  |  |  |  |
| -18040096 | 79952 | \x138b40106112221fa136213b8440c14661536157826fe479a4ca21eca1 | 13 | 11 | 12 |
| -17640096 | -2020048 | \x148b40084800084200c0208805104010902110062018201044430441080410000730401080220006618c82004020880200820004021080010042004080002018c240040108405080... | 2110 | 84 | 286 |
| -17540096 | -1720048 | \x138b4000c401010165040204a305e1082108620a020a420b640c0310c310e415021582166216a11701176218e11a011ae31b021ca11e611ee11f62200221a322a123442762286229... | 198 | 6 | 61 |
|  | -2020048 | \x138b40002200e101e10202028102e10302034103c1056405e1084108e2098109c20a410b610b840ba40bc20cc20d030d410d620d810da10ec20f4210641083110111421182122112... | 499 | 19 | 54 |
| -17440096 | -1620048 | \x138b40006200a300e10102014301a302210261038104c304e10703076208010841088209c109e30a420b820be20c410d010d810de10e010ec10f4210641141116112a3130113c214... | 485 | 20 | 127 |

## Load & store results from and to CSV¶

To export only aggregate counts (postcount, usercount) to CSV (e.g. for archive purposes):

**Store results to CSV for archive purposes:**

Define method:

In [122]:

```
def grid_agg_tocsv(
    grid: gp.GeoDataFrame, filename: str,
    metrics: List[str] = ["postcount_est", "usercount_est", "userdays_est"]):
    """Store geodataframe aggregate columns and indexes to CSV"""
    grid.to_csv(OUTPUT / f"csv{km_size_str}" / filename, mode='w', columns=metrics, index=True)
```

Convert/store to CSV (aggregate columns and indexes only):

In [123]:

```
grid_agg_tocsv(grid, "flickr_all_est.csv")
```

Repeat for Instagram

In [124]:

```
grid = load_pickle_merge(pickle_list_instagram, column_list)
```

In [125]:

```
grid.head()
```

Out[125]:

|  |  | geometry | postcount\_hll | postcount\_est | usercount\_est | userdays\_est |
| --- | --- | --- | --- | --- | --- | --- |
| xbin | ybin |  |  |  |  |  |
| -18040096 | 8979952 | POLYGON ((-18040096.000 8979952.000, -17940096.000 8979952.000, -17940096.000 8879952.000, -18040096.000 8879952.000, -18040096.000 8979952.000)) | NaN | 0 | 0 | 0 |
|  | 8879952 | POLYGON ((-18040096.000 8879952.000, -17940096.000 8879952.000, -17940096.000 8779952.000, -18040096.000 8779952.000, -18040096.000 8879952.000)) | NaN | 0 | 0 | 0 |
|  | 8779952 | POLYGON ((-18040096.000 8779952.000, -17940096.000 8779952.000, -17940096.000 8679952.000, -18040096.000 8679952.000, -18040096.000 8779952.000)) | NaN | 0 | 0 | 0 |
|  | 8679952 | POLYGON ((-18040096.000 8679952.000, -17940096.000 8679952.000, -17940096.000 8579952.000, -18040096.000 8579952.000, -18040096.000 8679952.000)) | NaN | 0 | 0 | 0 |
|  | 8579952 | POLYGON ((-18040096.000 8579952.000, -17940096.000 8579952.000, -17940096.000 8479952.000, -18040096.000 8479952.000, -18040096.000 8579952.000)) | NaN | 0 | 0 | 0 |

In [126]:

```
grid_agg_tocsv(grid, "instagram_random_est.csv")
```

In [127]:

```
def create_new_grid(
    grid_size: int = GRID_SIZE_METERS,
    proj_transformer: Transformer = PROJ_TRANSFORMER) -> gp.GeoDataFrame:
    """Create new 100x100km grid GeoDataFrame (Mollweide)"""
    # grid bounds from WGS1984 to Mollweide
    xmin = proj_transformer.transform(
        -180, 0)[0]
    xmax = proj_transformer.transform(
        180, 0)[0]
    ymax = proj_transformer.transform(
        0, 90)[1]
    ymin = proj_transformer.transform(
        0, -90)[1]
    grid = create_grid_df(
        grid_size=grid_size,
        xmin=xmin, ymin=ymin,
        xmax=xmax, ymax=ymax)
    # convert grid DataFrame to grid GeoDataFrame
    grid = grid_to_gdf(grid)
    return grid

def grid_agg_fromcsv(
    filename: str, columns: List[str] = ["xbin", "ybin"],
    metrics: List[str] = None, grid_size: int = GRID_SIZE_METERS):
    """Create a new Mollweide grid GeoDataFrame and 
    attach aggregate data columns from CSV based on index"""
    # 1. Create new 100x100km (e.g.) grid
    grid = create_new_grid(grid_size=grid_size)
    # 2. load aggregate data from CSV and attach to grid
    # -----
    if metrics is None:
        metrics = ["postcount_est", "usercount_est", "userdays_est"]
    types_dict = dict()
    for column in columns:
        if column.endswith('_hll'):
            types_dict[column] = str
    for metric in metrics:
        types_dict[metric] = int
    columns = columns + metrics
    # read only header first to check whether columns exist
    df = pd.read_csv(filename, usecols=columns, nrows=0)
    # remove columns if they do not exist in file
    columns[:] = [col for col in columns if col in (df.columns)]
    # load dataframe fully
    df = pd.read_csv(
        filename, dtype=types_dict,
        index_col=["xbin", "ybin"],
        usecols=columns)
    # join columns based on index
    grid = grid.join(df)
    # return grid with aggregate data attached
    return grid
```

To create a new grid and load aggregate counts from CSV:

In [128]:

```
grid = grid_agg_fromcsv(OUTPUT / f"csv{km_size_str}" / "flickr_all_est.csv")
```

In [129]:

```
grid[grid["usercount_est"]>5].head()
```

Out[129]:

|  |  | geometry | postcount\_est | usercount\_est | userdays\_est |
| --- | --- | --- | --- | --- | --- |
| xbin | ybin |  |  |  |  |
| -18040096 | 79952 | POLYGON ((-18040096.000 79952.000, -17940096.000 79952.000, -17940096.000 -20048.000, -18040096.000 -20048.000, -18040096.000 79952.000)) | 13 | 11 | 12 |
| -17640096 | -2020048 | POLYGON ((-17640096.000 -2020048.000, -17540096.000 -2020048.000, -17540096.000 -2120048.000, -17640096.000 -2120048.000, -17640096.000 -2020048.0... | 2110 | 84 | 286 |
| -17540096 | -1720048 | POLYGON ((-17540096.000 -1720048.000, -17440096.000 -1720048.000, -17440096.000 -1820048.000, -17540096.000 -1820048.000, -17540096.000 -1720048.0... | 198 | 6 | 61 |
|  | -2020048 | POLYGON ((-17540096.000 -2020048.000, -17440096.000 -2020048.000, -17440096.000 -2120048.000, -17540096.000 -2120048.000, -17540096.000 -2020048.0... | 499 | 19 | 54 |
| -17440096 | -1620048 | POLYGON ((-17440096.000 -1620048.000, -17340096.000 -1620048.000, -17340096.000 -1720048.000, -17440096.000 -1720048.000, -17440096.000 -1620048.0... | 485 | 20 | 127 |

**Store all other pickles as combined CSV (postcount, usercount, userdays)**

In [130]:

```
pickles_path = OUTPUT / f"pickles{km_size_str}"
# instagram sunset
pickle_list = [
    pickles_path / "instagram_postcount_sunset_est.pkl",
    pickles_path / "instagram_usercount_sunset_est.pkl",
    pickles_path / "instagram_userdays_sunset_est.pkl"
]
grid = load_pickle_merge(pickle_list, column_list)
grid_agg_tocsv(grid, "instagram_sunset_est.csv")
```

In [131]:

```
# instagram sunrise
pickle_list = [
    pickles_path / "instagram_postcount_sunrise_est.pkl",
    pickles_path / "instagram_usercount_sunrise_est.pkl",
    pickles_path / "instagram_userdays_sunrise_est.pkl"
]
grid = load_pickle_merge(pickle_list, column_list)
grid_agg_tocsv(grid, "instagram_sunrise_est.csv")
```

In [132]:

```
# instagram sunset+sunrise
pickle_list = [
    pickles_path / "instagram_postcount_sunsetsunrise_est.pkl",
    pickles_path / "instagram_usercount_sunsetsunrise_est.pkl",
    pickles_path / "instagram_userdays_sunsetsunrise_est.pkl"
]
grid = load_pickle_merge(pickle_list, column_list)
grid_agg_tocsv(grid, "instagram_sunsetsunrise_est.csv")
```

In [133]:

```
# flickr sunset
pickle_list = [
    pickles_path / "flickr_postcount_sunset_est.pkl",
    pickles_path / "flickr_usercount_sunset_est.pkl",
    pickles_path / "flickr_userdays_sunset_est.pkl"
]
grid = load_pickle_merge(pickle_list, column_list)
grid_agg_tocsv(grid, "flickr_sunset_est.csv")
```

In [134]:

```
# flickr sunrise
pickle_list = [
    pickles_path / "flickr_postcount_sunrise_est.pkl",
    pickles_path / "flickr_usercount_sunrise_est.pkl",
    pickles_path / "flickr_userdays_sunrise_est.pkl"
]
grid = load_pickle_merge(pickle_list, column_list)
grid_agg_tocsv(grid, "flickr_sunrise_est.csv")
```

# Close DB connection & Create notebook HTML¶

In [135]:

```
DB_CONN.close()
```

In [135]:

```
!jupyter nbconvert --to html_toc \
    --output-dir=../out/html{km_size_str} ./01_grid_agg.ipynb \
    --template=../nbconvert.tpl \
    --ExtractOutputPreprocessor.enabled=False >&- 2>&-  # create single output file
```

Copy new HTML file to resource folder

In [137]:

```
!cp ../out/html{km_size_str}/01_grid_agg.html ../resources/html/
```

In [ ]:

```

```
