# Supplementary material for: From sunrise to sunset: Exploring landscape preference through global reactions to ephemeral events captured in georeferenced social media
Source: PLoS One. 2023 Feb 22;18(2):e0280423. doi: 10.1371/journal.pone.0280423 (PMC9946259; doi:10.1371/journal.pone.0280423)
Supplement: S2 File — (HTML) [file pone.0280423.s002.html]

02\_visualization


# Interpretation: Interactive Visualization¶

*Alexander Dunkel, TU Dresden, Institute of Cartography; Maximilian Hartmann, Universität Zürich (UZH), Geocomputation*

---

•••

Out[3]:

Last updated: Jan-17-2023, Carto-Lab Docker Version 0.9.0

# Introduction¶

This is the second notebook in a series of nine notebooks:

1. the grid aggregation notebook (01\_gridagg.ipynb) is used to aggregate data from HLL sets at GeoHash 5 to a 100x100km grid
2. the visualization notebook (02\_visualization.ipynb) is used to create interactive maps, with additional information shown on hover
3. the chimaps notebook (03\_chimaps.ipynb) shows how to compute the chi square test per bin and event (sunset/sunrise).
4. the results notebook (04\_combine.ipynb) shows how to combine results from sunset/sunrise into a single interactive map.
5. Notebooks 5 to 9 are used for creating additional graphics and statistics.

# Preparations¶

## Parameters¶

For supplementing the final interactive graphic, define path to CSV data with the subset of Flickr photos published under Creative Commons. This includes the link to thumbnails, the CC-By Reference and the user name. This data is needed for accurate attribution of authors who explicitly published their photos for common use.

In [4]:

```
from pathlib import Path
root = Path.cwd().parents[1] / "00_hll_data"
SUNRISE_FLICKR_CCBY = root / "2020-04-07_Flickr_Sunrise_World_CCBy.csv"
SUNSET_FLICKR_CCBY = root / "2020-04-07_Flickr_Sunset_World_CCBy.csv"
```

## Load dependencies¶

In [5]:

```
import os, sys
import math
from pathlib import Path
from rtree import index
from IPython.display import Markdown
from bokeh.models import HoverTool, FixedTicker
from bokeh.io import export_svgs
```

In [6]:

```
module_path = str(Path.cwd().parents[0] / "py")
if module_path not in sys.path:
    sys.path.append(module_path)
from modules import preparations
```

In [7]:

```
preparations.init_imports()
```

Prepare svg export (if chromedriver found). To install chromedriver in Linux, see 1.

In [8]:

```
WEB_DRIVER = preparations.load_chromedriver()
```

```
[WDM] - Downloading: 100%|██████████| 6.96M/6.96M [00:00<00:00, 59.6MB/s]
```

```
Chromedriver loaded. Svg output enabled.
```

## Import first notebook¶

We're going to use many methods and the parameters defined in the previous notebook. These are imported from the jupytext converted python script file:

In [9]:

```
from _01_grid_agg import *
```

Importing all methods to local namespace is typically considered an anti-pattern. In this case, however, we can continue working with parameters and methods as if they were defined in this notebook.

To test that methods and parameters from previous notebook are available:

In [10]:

```
display(GRID_SIZE_METERS)
```

```
100000
```

Activate autoreload of changed python files:

In [11]:

```
%load_ext autoreload
%autoreload 2
```

## Load aggregate grid data¶

Restore grid (geodataframe) from CSV using the methods defined in previous notebook.

**Read benchmark data, only loading userdays\_hll column.**

In [12]:

```
grid = grid_agg_fromcsv(
    OUTPUT / f"csv{km_size_str}" / "flickr_sunset_est.csv")
```

# Interactive Map with Holoviews/ Geoviews¶

Geoviews and Holoviews can be used to create interactive maps, either insider Jupyter or as externally stored as HTML. The syntax of the plot methods must be slightly adapted from the matplotlib output. Given the advanced features of interactivity, we can also add additional information that is shown e.g. on mouse hover over certain grid cells.

### Define base plotting functions¶

We'll need to add some methods to the static mapping methods defined in `01_grid_agg.ipynb`

**Prepare interactive mapping**

Below methods are adapted for label processing and classification that is compatible with holoviews/geoviews.

These methods already include code for mapping significance, chi values and under/overrepresentation,
which are included in subsequent notebooks.

In [13]:

```
import warnings
def mask_series(
        grid: gp.GeoDataFrame,
        metric: str = "postcount_est",
        mask_nonsignificant: bool = None,
        mask_negative: bool = None,
        mask_positive: bool = None) -> pd.Series:
    """Create masks for selecting data"""
    if mask_negative and mask_positive:
        warnings.warn("Masking both positive and negative values!")
    mask = grid[metric].notna()
    # mask the values to be considered
    if mask_nonsignificant:
        mask = (mask) & (grid['significant'])
    if mask_negative:
        mask = (mask) & (grid[metric] > 0)
    if mask_positive:
        mask = (mask) & (grid[metric] < 0)
    # render zero as transparent,
    # ~ returns an inverted mask
    mask = (mask) & (~grid[metric].isin([0,-0]))
    # get value series
    # excluding NaN values
    series_nan = grid.loc[
        mask, metric].dropna().astype(float)
    return series_nan

def get_scheme_breaks(series_nan: pd.Series, scheme: str):
    """Classify series of values
    
    Notes: some classification schemes (e.g. HeadTailBreaks)
        do not support specifying the number of classes returned
        construct optional kwargs with k == number of classes
    """
    optional_kwargs = {"k":9}
    if scheme == "HeadTailBreaks":
        optional_kwargs = {}
    scheme_breaks = mc.classify(
        y=np.abs(series_nan.values), scheme=scheme, **optional_kwargs)
    return scheme_breaks

def classify_data(
        values_series: pd.Series,
        scheme: str):
    """Classify data (value series) and return classes,
       bounds, and colormap
       
    Args:
        values_series: A pandas.Series with metric column to classify
        scheme: The classification scheme to use.
        
    Adapted from:
        https://stackoverflow.com/a/58160985/4556479
    See available colormaps:
        http://holoviews.org/user_guide/Colormaps.html
    See available classification schemes:
        https://pysal.org/mapclassify/api.html
    """
    scheme_breaks = get_scheme_breaks(values_series, scheme)
    # get label bounds as flat array
    bounds = get_label_bounds(
        scheme_breaks.bins, np.abs(values_series.values), flat=True)
    return bounds, scheme_breaks

def get_hex_col(cmap) -> List[str]:
    """Return list of hex colors for cmap"""
    return [colors.rgb2hex(cmap(i)) for i in range(cmap.N)]

def get_cmap_list(
        cmap_name: str, length_n: int) -> [str]:
    """Create a classified colormap of length N
    """
    if cmap_name in ["OrRd", "Blues"]:
        # special color-mod for OrRd and Blues
        cmap = plt.cm.get_cmap(cmap_name, length_n)
        cmap_list = get_hex_col(cmap)
        cmap_color_contrast(cmap_list, cmap_name)
        return cmap_list
    # general case:
    # increase contrast of first color,
    # by extending and pop(0)
    cmap = plt.cm.get_cmap(cmap_name, length_n+1)
    cmap_list = get_hex_col(cmap)
    cmap_list.pop(0)
    if length_n != len (cmap_list):
        warnings.warn("Cmap list length mismatch")
    return cmap_list

def cmap_color_contrast(cmap: List[str], cname: str):
    """Minor contrast increase to the first and last colors in
    matplotlib OrRd and Blues"""
    if cname == "OrRd":
        # darken all colors by 
        # offset color list by 1
        lastcolor = '#440402'
        cmap.append(lastcolor)
        # remove first color that is
        # too light on white background
        del cmap[0]
        # lighten new first color a bit
        firstcolor = '#FCF0D8'
        cmap[0] = firstcolor
    if cname == "Blues":
        lastcolor = '#06214a'
        cmap.append(lastcolor)
        del cmap[0]
        firstcolor = '#DEE9F5'
        cmap[0] = firstcolor
    
def prepend_color(cmap_list: [str], color_hex: str):
    """manually prepend color to ListedColormap"""
    cmap_list.insert(0, color_hex)
```

Some helper functions first. Also added here is the option to show additional columns on hover, which will be later used to show chi\_value and significance.

In [14]:

```
def get_custom_tooltips(items: Dict[str, str]) -> str:
    """Compile HoverTool tooltip formatting with items to show on hover"""
    # thousands delimitor formatting
    # will be applied to the following columns
    tdelim_format = [
        'usercount_est', 'postcount_est', 'userdays_est']
    # in HoloViews, custom tooltip formatting can be
    # provided as a list of tuples (name, value)
    tooltips=[
        # f-strings explanation:
        # - k: the item name, v: the item value,
        # - @ means: get value from column
        # optional formatting is applied using
        # `"{,f}" if v in thousand_formats else ""`
        # - {,f} means: add comma as thousand delimiter
        # only for values in tdelim_format (usercount and postcount)
        # else apply automatic format
        (k, 
         f'@{v}{"{,f}" if v.replace("_expected", "") in tdelim_format else ""}'
        ) for k, v in items.items()
    ]
    return tooltips

def set_active_tool(plot, element):
    """Enable wheel_zoom in bokeh plot by default"""
    plot.state.toolbar.active_scroll = plot.state.tools[0]

def convert_gdf_to_gvimage(
        grid: gp.GeoDataFrame, metric: str, cat_count: Optional[int] = None, 
        cat_min: Optional[int] = None, cat_max: Optional[int] = None,
        hover_items: Dict[str, str] = None) -> gv.Image:
    """Convert GeoDataFrame to gv.Image using categorized
    metric column as value dimension
    
    Args:
        grid: A geopandas geodataframe with indexes x and y 
            (projected coordinates) and aggregate metric column
        metric: target column for value dimension.
            "_cat" will be added to retrieve classified values.
        cat_count: number of classes for value dimension
        hover_items: a dictionary with optional names 
            and column references that are included in 
            gv.Image to provide additional information
            (e.g. on hover)
    """
    if cat_count:
        cat_min = 0
        cat_max = cat_count
    else:
        if any([cat_min, cat_max]) is None:
            raise ValueError(
                "Either provide cat_count or cat_min and cat_max.")
    if hover_items is None:
        hover_items_list = []
    else:
        hover_items_list = [
            v for v in hover_items.values()]
    # convert GeoDataFrame to xarray object
    # the first vdim is the value being used 
    # to visualize classes on the map
    # include hover_items (postcount and usercount)
    # to show exact information through tooltip
    xa_dataset = gv.Dataset(
        grid.to_xarray(),
        vdims=[
            hv.Dimension(
                f'{metric}_cat', range=(cat_min, cat_max))]
            + hover_items_list,
        crs=crs.Mollweide())
    return xa_dataset.to(gv.Image, crs=crs.Mollweide())

def assign_special_categories(
        grid: gp.GeoDataFrame, values_series,
        metric, add_nodata_label,
        mask_nonsignificant):
    """Assign special categories to _cat column"""
    if add_nodata_label:
        # nodata label as explicit category in legend
        # values will not be rendered on map (cat = nan)
        # increment cat count
        grid[f'{metric}_cat'] += 1
        # Select inverted index for map values
        grid.loc[
            ~grid.index.isin(values_series.index),
            f'{metric}_cat'] = np.nan
    if mask_nonsignificant:
        # also set none-significant values to nan
        # to not affect classification (cat = nan)
        grid.loc[
            grid.significant == False,
            f'{metric}_cat'] = np.nan

def create_labels(
        cmap_list, bounds, add_nodata_label: str = None,
        mask_nonsignificant: bool = None) -> Dict[int, str]:
    """Create labels, add special categories to cmap"""
    # construct legend labels for colormap
    # get colormap
    label_dict = {}
    color_levels = len(bounds)
    # specific label cases
    spare_cats = 0
    if add_nodata_label:
        spare_cats = 1
        label_dict[0] = "No data"
    # process regular labels
    for idx, value in enumerate(bounds):
        label_dict[idx+spare_cats] = value
    if add_nodata_label and mask_nonsignificant:
        label_dict[spare_cats-1] = \
        f'{label_dict[spare_cats-1]} or not significant'
    return label_dict
```

Since interactive display of grid-polygons is too slow, we are converting the grid to an xarray object, which is then overlayed as a rastered image.

In [15]:

```
def get_classify_image(grid: gp.GeoDataFrame,
    metric: str = "postcount_est", responsive: bool = None,
    hover_items: Dict[str, str] = {
        'Post Count (estimated)':'postcount_est', 
        'User Count (estimated)':'usercount_est',
        'User Days (estimated)':'userdays_est'},
    mask_nonsignificant: bool = False,
    scheme: str = "HeadTailBreaks",
    cmap_name: str = "OrRd",
    add_nodata_label: str = "#FFFFFF"):
    """Create gv image layer from geodataframe (grid)

    Args:
        grid: A geopandas geodataframe with indexes x and y 
            (projected coordinates) and aggregate metric column
        metric: target column for aggregate. Default: postcount.
        responsive: Should be True for interactive HTML output.
        hover_items: additional items to show on hover
        dim_nonsignificant: transparent bins if significant column == False
        scheme: The classification scheme to use. Default "HeadTailBreaks".
        cmap: The colormap to use. Default "OrRd".
        inverse: plot other map elements inverse (black)
    """

    # get value series, excluding special categories
    kwargs = {
        "mask_nonsignificant":mask_nonsignificant
    }
    series_nan = mask_series(
        grid=grid, metric=metric, **kwargs)
    # classify values
    bounds, scheme_breaks = classify_data(
        values_series=series_nan, scheme=scheme)
    # assign categories column
    grid.loc[series_nan.index, f'{metric}_cat'] = scheme_breaks.find_bin(
        series_nan)
    # assign special categories (nodata, not significant, not representative)
    assign_special_categories(
        grid=grid, values_series=series_nan,
        metric=metric, add_nodata_label=add_nodata_label, **kwargs)
    cat_count = scheme_breaks.k
    cmap_list = get_cmap_list(cmap_name, length_n=cat_count)
    # spare cats are added to legend,
    # but have no representation on the map
    # (e.g. White "No Data" Label)
    spare_cats = 0
    if add_nodata_label:
        spare_cats = 1
        prepend_color(
            cmap_list=cmap_list, color_hex=add_nodata_label)
        kwargs["add_nodata_label"] = add_nodata_label
    # create cmap and labels
    label_dict = create_labels(
        cmap_list, bounds, **kwargs)
    cmap = colors.ListedColormap(cmap_list)
    # create gv.Image layer from gdf
    img_grid = convert_gdf_to_gvimage(
            grid=grid,
            metric=metric, cat_count=cat_count+spare_cats,
            hover_items=hover_items)
    return img_grid, cmap, label_dict
```

Plot compilation (layers, styling, legend)

In [16]:

```
def apply_layer_opts(
    img_grid: gv.Image, cmap: colors.ListedColormap,
    label_dict: Dict[str, str], responsive: bool = None,
    hover_items: Dict[str, str] = None) -> gv.Image:
    """Apply geoviews image layer opts

    Args:
        img_grid: A classified gv.Image layer
        responsive: Should be True for interactive HTML output.
        hover_items: additional items to show on hover
        cmap: A matplotlib colormap to colorize values and show as legend.
    """
    if hover_items is None:
        hover_items = {
        'Post Count (estimated)':'postcount_est', 
        'User Count (estimated)':'usercount_est',
        'User Days (estimated)':'userdays_est'}
    color_levels = len(cmap.colors)
    # define additional plotting parameters
    # width of static jupyter map,
    # 360° == 1200px
    width = 1200 
    # height of static jupyter map,
    # 360°/2 == 180° == 600px
    height = int(width/2) 
    aspect = None
    # if stored as html,
    # override values
    if responsive:
        width = None
        height = None
    # define width and height as optional parameters
    # only used when plotting inside jupyter
    optional_kwargs = dict(width=width, height=height)
    # compile only values that are not None into kwargs-dict
    # by using dict-comprehension
    optional_kwargs_unpack = {
        k: v for k, v in optional_kwargs.items() if v is not None}
    # prepare custom HoverTool
    tooltips = get_custom_tooltips(
        hover_items)
    hover = HoverTool(tooltips=tooltips)
    # get tick positions from label dict keys
    ticks = [key for key in sorted(label_dict)]
    # create image layer
    image_layer = img_grid.opts(
            color_levels=color_levels,
            cmap=cmap,
            colorbar=True,
            clipping_colors={'NaN': 'transparent'},
            colorbar_opts={
                # 'formatter': formatter,
                'major_label_text_align':'left',
                'major_label_overrides': label_dict,
                'ticker': FixedTicker(
                    ticks=ticks),
                },
            tools=[hover],
            # optional unpack of width and height
            **optional_kwargs_unpack
        )
    return image_layer
```

In [17]:

```
def compile_image_layer(grid: gp.GeoDataFrame,
    metric: str = "postcount_est", responsive: bool = None,
    hover_items: Dict[str, str] = None,
    mask_nonsignificant: bool = False,
    scheme: str = "HeadTailBreaks",
    cmap_name: str = "OrRd",
    add_nodata_label: str = "#FFFFFF"):
    """Compile geoviews image layer from grid

    Args:
        grid: A geopandas geodataframe with indexes x and y 
            (projected coordinates) and aggregate metric column
        metric: target column for aggregate. Default: postcount.
        responsive: Should be True for interactive HTML output.
        hover_items: additional items to show on hover
        dim_nonsignificant: transparent bins if significant column == False
        scheme: The classification scheme to use. Default "HeadTailBreaks".
        cmap: The colormap to use. Default "OrRd".
    """
    # work on a shallow copy,
    # to not modify original dataframe
    grid_plot = grid.copy()
    kwargs = {
        "grid":grid_plot,
        "metric":metric,
        "hover_items":hover_items,
        "mask_nonsignificant":mask_nonsignificant,
        "scheme":scheme, "cmap_name":cmap_name,
        "add_nodata_label":add_nodata_label
    }
    # get gv.Image layer, cmap, and label dict (legend)
    img_grid, cmap, label_dict = get_classify_image(**kwargs)
    # apply display opts to gv.Image layer
    image_layer = apply_layer_opts(
        img_grid=img_grid, cmap=cmap, label_dict=label_dict,
        responsive=responsive, hover_items=hover_items)
    return image_layer
```

In [18]:

```
def combine_gv_layers(
        image_layer: gv.Image, edgecolor: str = 'black',
        fill_color: str = '#dbdbdb', alpha: float = 0.15,
        additional_layers = None) -> gv.Overlay:
    """Combine layers into single overlay and set global plot options"""
    # fill_color = '#479AD4'
    # fill_color = '#E9EDEC'
    gv_layers = []
    gv_layers.append(
        gf.land.opts(
            alpha=alpha, fill_color=fill_color))
    gv_layers.append(
        gf.ocean.opts(
            alpha=alpha, fill_color=fill_color))
    gv_layers.append(
        image_layer)
    gv_layers.append(
        gf.coastline.opts(
            line_color=edgecolor)) 
    gv_layers.append(
        gf.borders.opts(
            line_color=edgecolor))
    if additional_layers:
        gv_layers.extend(additional_layers)
    return gv.Overlay(gv_layers)

def get_annotation_layer(
        sel_layer1: gv.Points, sel_layer2: gv.Points = None,
        sel_labels1: List[gv.Text] = None, sel_labels2: List[gv.Text] = None):
    """Prepare annotation layer
    
    add layer(s) for highlighting special grid cells (largest, smallest)
    see marker symbols
    https://docs.bokeh.org/en/2.4.1/docs/reference/models/markers.html#scatter
    """
    gv_layers = []
    gv_layers.append(
        sel_layer1.opts(
            line_color='black', fill_color=None, marker='square', 
            size=20, alpha=0.8))
    if sel_labels1:
        gv_layers.extend(sel_labels1)
    if sel_layer2:
        gv_layers.append(
            sel_layer2.opts(
                line_color='black', fill_color=None, marker='triangle', 
                size=20, alpha=0.8))
    if sel_labels2:
        gv_layers.extend(sel_labels2)
    return gv_layers
```

High level plotting function

In [19]:

```
def plot_interactive(grid: gp.GeoDataFrame, title: str,
    metric: str = "postcount_est",
    hover_items: Dict[str, str] = {
        'Post Count (estimated)':'postcount_est', 
        'User Count (estimated)':'usercount_est',
        'User Days (estimated)':'userdays_est'},
    mask_nonsignificant: bool = False,
    scheme: str = "HeadTailBreaks",
    cmap: str = "OrRd",
    store_html: str = None,
    plot: Optional[bool] = True,
    output: Optional[str] = OUTPUT,
    nodata_color = "#FFFFFF") -> gv.Overlay:
    """Plot interactive map with holoviews/geoviews renderer

    Args:
        grid: A geopandas geodataframe with indexes x and y 
            (projected coordinates) and aggregate metric column
        metric: target column for aggregate. Default: postcount.
        store_html: Provide a name to store figure as interactive HTML.
        title: Title of the map
        hover_items: additional items to show on hover
        mask_nonsignificant: transparent bins if significant column == False
        scheme: The classification scheme to use. Default "HeadTailBreaks".
        cmap: The colormap to use. Default "OrRd".
        plot: Prepare gv-layers to be plotted in notebook.
    """
     # check if all additional items are available
    for key, item in list(hover_items.items()):
        if item not in grid.columns:
            hover_items.pop(key)
    # chi layer opts
    # global plotting options for values layer
    layer_opts = {
        "metric":metric,
        "responsive":False,
        "mask_nonsignificant":mask_nonsignificant,
        "scheme":scheme,
        "hover_items":hover_items,
        "cmap_name":cmap,
        "add_nodata_label":nodata_color
    }
    # global plotting options for all layers (gv.Overlay)
    gv_opts = {
        "bgcolor":None,
        # "global_extent":True,
        "projection":crs.Mollweide(),
        "responsive":False,
        "data_aspect":1, # maintain fixed aspect ratio during responsive resize
        "hooks":[set_active_tool],
        "title":title
    }
    sel_points = grid.nlargest(5, metric).centroid
    # add y offset to correct for 1/2 bin length
    sel_layer = tools.series_to_point(sel_points, mod_y = GRID_SIZE_METERS/2)
    sel_labels = tools.series_to_label(sel_points)
    annotation_layers = get_annotation_layer(sel_layer1=sel_layer, sel_labels1=sel_labels)
    if plot:
        # get classified xarray gv image layer
        image_layer = compile_image_layer(
            grid=grid, **layer_opts)
        gv_layers = combine_gv_layers(
            image_layer, fill_color=nodata_color, alpha=0.5, 
            additional_layers=annotation_layers)
    if store_html:
        layer_opts["responsive"] = True
        image_layer = compile_image_layer(
            grid=grid, **layer_opts)
        responsive_gv_layers = combine_gv_layers(
            image_layer, fill_color=nodata_color, alpha=0.5,
            additional_layers=annotation_layers)
        gv_opts["responsive"] = True
        export_layers = responsive_gv_layers.opts(**gv_opts)
        hv.save(
            export_layers,
            output / f"html{km_size_str}" / f'{store_html}.html', backend='bokeh')
        if WEB_DRIVER:
            # store also as svg
            p =  hv.render(export_layers, backend='bokeh')
            p.output_backend = "svg"
            export_svgs(p, 
                filename=output / f"svg{km_size_str}" / f'{store_html}.svg',
                webdriver=WEB_DRIVER)
    if not plot:
        return
    gv_opts["responsive"] = False
    return gv_layers.opts(**gv_opts)
```

**plot interactive in-line:**

In [20]:

```
filename = "flickr_sunset_userdays_est"
gv_plot = plot_interactive(
    grid, title=f'Flickr User Days (estimated) for "sunset" per {km_size:.0f} km grid', metric="userdays_est",
    store_html=filename)
gv_plot
```

Out[20]:

View the interactive map here and hover over bins to view values.

**Repeat for sunrise**

In [21]:

```
grid = grid_agg_fromcsv(
    OUTPUT / f"csv{km_size_str}" / "flickr_sunrise_est.csv")
```

In [22]:

```
gv_plot = plot_interactive(
    grid, title=f'Flickr User Days (estimated) for "sunrise" per {km_size:.0f} km grid', metric="userdays_est",
    cmap="Blues", store_html="flickr_sunrise_userdays_est")
gv_plot
```

Out[22]:

### Attach CC-BY thumbnail URLs¶

The HoverTool can be used to provide additional information on each grid cell on hover. The information must be included in the gv.Image as additional columns.

It is also possible to load pictures using an url (see the bokeh docs)

Since some of our pictures contain CC-BY licenses, we can display those as examples using the provided Flickr URL. We're going to override the `get_custom_tooltips()` method for showing images.

In [23]:

```
cc_by_ref = {
  1:"CC-BY-NC-SA 2.0",
  2:"CC-BY-NC 2.0",
  3:"CC-BY-NC-ND 2.0",
  4:"CC-BY 2.0",
  5:"CC-BY-SA 2.0",
  6:"CC-BY-ND 2.0",
  7:"No known copyright restrictions",
  8:"United States Government Work",
  9:"Public Domain Dedication (CC0)",
  10:"Public Domain Mark"
}
```

Get list of xbins and ybins for best assignment:

In [24]:

```
_, rows, cols = create_grid_df(return_rows_cols=True)
```

In [25]:

```
YBINS = np.array(rows)
XBINS = np.array(cols)
```

In [26]:

```
def append_thumbs_grid(
    df: pd.DataFrame, grid: gp.GeoDataFrame,
    xbins: np.array = XBINS, ybins: np.array = YBINS) -> gp.GeoDataFrame:
    """Append thumbails to grid based on viewcount
    
        Args:
        df: A pandas dataframe with latitude and 
            longitude columns in WGS1984
        grid: A geopandas geodataframe with indexes 
            x and y (projected coordinates) and grid polys
        xbins: 1-d array of bins to snap lat/lng values
        ybins: 1-d array of bins to snap lat/lng values
    """
    # bin assigment of projected coordinates
    # df[['xbinx_match','ybins_match']] = 
    xbins_match, ybins_match = get_best_bins(
        search_values_x=df['x'].to_numpy(),
        search_values_y=df['y'].to_numpy(),
        xbins=xbins, ybins=ybins)
    # append target bins to original dataframe
    df.loc[:, 'xbins_match'] = xbins_match
    df.loc[:, 'ybins_match'] = ybins_match
    # now we'll loop values of the dataframe,
    # only remembering the image url of the 
    # top viewed image per grid
    grid.loc[:, "cc_img_url"] = ""
    grid.loc[:, "cc_img_views"] = 0
    x = 0
    cc_total = len(df)
    grid.loc[:, "post_content_license"] = ""
    for __, row in df.iterrows():
        if grid.loc[
                (row.xbins_match,
                 row.ybins_match),
                "cc_img_views"] < row.post_views_count:
            grid.loc[
                (row.xbins_match,
                 row.ybins_match),
                "cc_img_views"] = row.post_views_count
            grid.loc[
                (row.xbins_match,
                 row.ybins_match),
                "cc_img_url"] = row.post_thumbnail_url
            grid.loc[
                (row.xbins_match,
                 row.ybins_match),
                "post_content_license"] = row.post_content_license
        x += 1
        if x % 100 == 0:
            clear_output(wait=True)
            print(
                f'Attaching CC thumbails. Processed {x} of '
                f'{cc_total} CC Images ({x/(cc_total/100):.2f}%).')

def merge_thumbs_grid(
    grid_thumbs: gp.GeoDataFrame, grid: gp.GeoDataFrame, mask: Optional[pd.Series] = None):
    """Merge thumbnail grid data to grid geodataframe
    
    Args:
        grid_thumbs: Indexed GeoDataFrame with thumbnail data
        grid: Indexed GeoDataFrame target
        mask: Optional boolean mask (pd.Series)
              to partially merge thumb data"""
    usecols = [
        'cc_img_url', 'cc_img_views', 'post_content_license']
    for col in usecols:
        if mask is None:
            grid.loc[grid_thumbs.index, col] = grid_thumbs[col]
            continue
        grid_thumnbs_mask = grid_thumbs.loc[mask, col]
        grid.loc[grid_thumnbs_mask.index, col] = grid_thumnbs_mask
```

In [27]:

```
def attach_ccthumbnail_urls(
    grid: gp.GeoDataFrame, data: Path = None, pickle: Path = None, 
    mask: Optional[pd.Series] = None):
    """Attach ccthumbnail urls to grid
    
        Args:
        grid: A geopandas geodataframe with indexes x and y 
            (projected coordinates) and grid polys
        data: Path to read input CSV with Flickr CCBy data (optional)
        pickle: Path with binned and pickled GeoDataFrame (optional).
            Either data or pickle must be supplied.
    """
    if data is None and pickle is None:
        raise ValueError("Provide either path to data or pickle.")
    if pickle:
        grid_thumb = pd.read_pickle(pickle)
        merge_thumbs_grid(grid_thumbs=grid_thumb, grid=grid, mask=mask)
        return
    # read thumbs from source (CSV)
    usecols = [
        'latitude', 'longitude', 'post_thumbnail_url',
        'post_views_count', 'post_content_license']
    dtypes = {
        'latitude': float, 'longitude': float}
    df = pd.read_csv(
        data, usecols=usecols, dtype=dtypes, encoding='utf-8')
    # filter_null_island:
    filter_nullisland_df(df)
    # Only use images where thumbnails are available
    df.query(
        '(post_thumbnail_url.isnull()) == False', inplace=True)
    # replace licenses with actual text
    df['post_content_license'] = df['post_content_license'].apply(
        lambda x: cc_by_ref.get(x, ""))
    # project dataframe
    proj_df(df)
    # reset metric column
    append_thumbs_grid(
        df=df, grid=grid)
```

Create a new grid and store the single most viewed picture thumbnail per bin:

In [28]:

```
%%time
cc_thumbs_sunset = OUTPUT / f"pickles{km_size_str}" / "flickr_ccbythumbnails_sunset.pkl"
if not cc_thumbs_sunset.exists():
    sunset_thumb_grid = create_new_grid()
    attach_ccthumbnail_urls(
        data=SUNSET_FLICKR_CCBY, grid=sunset_thumb_grid)
    sunset_thumb_grid.to_pickle(
        cc_thumbs_sunset)
```

```
Attaching CC thumbails. Processed 284900 of 284989 CC Images (99.97%).
CPU times: user 51.8 s, sys: 1.53 s, total: 53.3 s
Wall time: 52.8 s
```

In [29]:

```
cc_thumbs_sunrise  = OUTPUT / f"pickles{km_size_str}" / "flickr_ccbythumbnails_sunrise.pkl"
if not cc_thumbs_sunrise.exists():
    sunrise_thumb_grid = create_new_grid()
    attach_ccthumbnail_urls(
        data=SUNRISE_FLICKR_CCBY, grid=sunrise_thumb_grid)
    sunrise_thumb_grid.to_pickle(
        cc_thumbs_sunrise)
```

```
Attaching CC thumbails. Processed 82800 of 82852 CC Images (99.94%).
```

**Attach thumbnails from pickle to the grid:**

In [30]:

```
%%time
attach_ccthumbnail_urls(
    grid, pickle=cc_thumbs_sunset)
```

```
CPU times: user 724 ms, sys: 24.1 ms, total: 748 ms
Wall time: 747 ms
```

**Override tooltips definition:**

In [31]:

```
def get_custom_tooltips(items: Dict[str, str]) -> str:
    """Compile HoverTool tooltip formatting with items to show on hover
    including showing a thumbail image from a url"""
    tdelim_format = [
        'usercount_est', 'postcount_est', 'userdays_est']
    # format html
    tooltips = "".join(
        f'<div><span style="font-size: 12px;">'
        f'<span style="color: #82C3EA;">{k}:</span> '
        f'@{v}{"{,f}" if v.replace("_expected", "") in tdelim_format else ""}'
        f'</span></div>' for k, v in items.items() if v not in ["cc_img_url", "post_content_license"])
    if 'cc_img_url' in items.values():
        tooltips += f'''<div><img src="@cc_img_url" alt="No CC Image available" style="height:170px"></img></div>'''
        tooltips += '''@post_content_license'''
    return tooltips
```

configure what should be shown on mouse hover tooltip

In [32]:

```
hover_items = { 
    '"Sunrise" Post Count (est)':'postcount_est',
    '"Sunrise" User Count (est)':'usercount_est',
    '"Sunrise" User Days (est)':'userdays_est'}
hover_items_thumbs = {
    'CC-Top-Image-ViewCount':'cc_img_views',
    'CC-License':'post_content_license',
    'CC-Image-URL':'cc_img_url'}
if 'cc_img_views' in grid.columns:
    hover_items.update(hover_items_thumbs)
```

In [33]:

```
gv_plot = plot_interactive(
    grid, title=f'Flickr User Days (estimated) for "sunrise" per {km_size:.0f} km grid', metric="userdays_est",
    cmap="Blues", store_html="flickr_sunrise_userdays_est", hover_items=hover_items)
gv_plot
```

Out[33]:

# Interactive Figure comparison with HTML tabs¶

In the last notebook, several figures were generated and stored as png. Create a simple HTML with tabs to compare **Post Count, User Count and User Days** and **Instagram and Flickr** and **Sunset and Sunrise**

In [34]:

```
import ipywidgets as widgets
# dictionary with filename and title
pathrefs = {
    0: ('flickr_postcount_all_est.png', 'Post Count (Flickr total)'),
    1: ('flickr_usercount_all_est.png', 'User Count (Flickr total)'),
    2: ('flickr_userdays_all_est.png', 'User Days (Flickr total)'),
    3: ('flickr_postcount_sunrise_est.png', 'Post Count (Flickr "sunrise")'),
    4: ('flickr_usercount_sunrise_est.png', 'User Count (Flickr "sunrise")'),
    5: ('flickr_userdays_sunrise_est.png', 'User Days (Flickr "sunrise")'),
    6: ('flickr_postcount_sunset_est.png', 'Post Count (Flickr "sunset")'),
    7: ('flickr_usercount_sunset_est.png', 'User Count (Flickr "sunset")'),
    8: ('flickr_userdays_sunset_est.png', 'User Days (Flickr "sunset")'),
    9: ('instagram_postcount_sunrise_est.png', 'Post Count (Instagram "sunrise")'),
    10: ('instagram_usercount_sunrise_est.png', 'User Count (Instagram "sunrise")'),
    11: ('instagram_userdays_sunrise_est.png', 'User Days (Instagram "sunrise")'),
    12: ('instagram_postcount_sunset_est.png', 'Post Count (Instagram "sunset")'),
    13: ('instagram_usercount_sunset_est.png', 'User Count (Instagram "sunset")'),
    14: ('instagram_userdays_sunset_est.png', 'User Days (Instagram "sunset")'),
    15: ('instagram_usercount_sunsetsunrise_est.png', 'User Count (Instagram "sunset+sunrise")'),
    }

def get_img_width(filename: str):
    if 'sample' in filename:
        return 700
    return 1300

widgets_images = [
    widgets.Image(
        value=open(Path('OUT') / OUTPUT / f"figures{km_size_str}" / pathref[0], "rb").read(),
        format='png',
        width=get_img_width(pathref[0])
     )
    for pathref in pathrefs.values()]
```

Configure tabs

In [35]:

```
children = widgets_images
tab = widgets.Tab()
tab.children = children
for i in range(len(children)):
    tab.set_title(i, pathrefs[i][1])
```

Display inside live notebook:

In [36]:

```
tab
```

Out[36]:

```
Tab(children=(Image(value=b'\x89PNG\r\n\x1a\n\x00\x00\x00\rIHDR\x00\x00\x16\xef\x00\x00\r!\x08\x06\x00\x00\x00…
```

The above tab display is not available in the static notebook HTML export. A standalone HTML with the above tabs can be generated with the following command:

In [37]:

```
from ipywidgets.embed import embed_minimal_html
embed_minimal_html(
    Path('OUT') / OUTPUT / f'html{km_size_str}' / 'compare_figures.html',
    views=[tab], title='Worldwide sunset + sunrise reactions (Instagram and Flickr) compared for different metrics')
```

View the result here.

# Create notebook HTML¶

In [46]:

```
!jupyter nbconvert --to html_toc \
    --output-dir=../out/html{km_size_str} ./02_visualization.ipynb \
    --template=../nbconvert.tpl \
    --ExtractOutputPreprocessor.enabled=False >&- 2>&- # create single output file
```

Copy new HTML file to resource folder

In [47]:

```
!cp ../out/html{km_size_str}/02_visualization.html ../resources/html/
```

In [ ]:

```

```
