# Supplementary material for: From sunrise to sunset: Exploring landscape preference through global reactions to ephemeral events captured in georeferenced social media
Source: PLoS One. 2023 Feb 22;18(2):e0280423. doi: 10.1371/journal.pone.0280423 (PMC9946259; doi:10.1371/journal.pone.0280423)
Supplement: S3 File — (HTML) [file pone.0280423.s003.html]

03\_chimaps


# Interpretation: Chi-Square Test¶

*Alexander Dunkel, TU Dresden, Institute of Cartography; Maximilian Hartmann, Universität Zürich (UZH), Geocomputation*

---

•••

Out[1]:

Last updated: Jan-16-2023, Carto-Lab Docker Version 0.9.0

# Introduction¶

This is the third notebook in a series of nine notebooks:

1. the grid aggregation notebook (01\_gridagg.ipynb) is used to aggregate data from HLL sets at GeoHash 5 to a 100x100km grid
2. the visualization notebook (02\_visualization.ipynb) is used to create interactive maps, with additional information shown on hover
3. the chimaps notebook (03\_chimaps.ipynb) shows how to compute the chi square test per bin and event (sunset/sunrise).
4. the results notebook (04\_combine.ipynb) shows how to combine results from sunset/sunrise into a single interactive map.
5. Notebooks 5 to 9 are used for creating additional graphics and statistics.

# Preparations¶

## Parameters¶

Pearson's chi-squared test can be used to evaluate whether there is a statistically significant difference between the expected frequencies (e.g. all reactions on Flickr) and the observed frequencies (e.g. reactions to sunset on Flickr). This type of chi-test belongs to the category of Goodness of Fit Tests. We'll calculate the Chi-square for each bin. In here, we use a specific version called the `signed` chi square, which is suitble for mapping over- and underrepresentation.

First, define the input parameter:

- **dof**: degrees of freedom (dof) is calculated: (variables - 1) with the variables being: observed posts, expected posts
- **chi\_crit\_val**: given a dof value of 1 and a confidence interval of 0.05, the critical value to accept or neglect the h0 is 3.84
- **chi\_column**: we'll do the chi calculation based on the usercount-column, but this notebook can be run for postcount or userdays, too.

In [2]:

```
DOF = 1
CHI_CRIT_VAL = 3.84
CHI_COLUMN = "usercount_est"
```

In [3]:

```
METRIC_NAME_REF = {
    "postcount_est":"Post Count",
    "usercount_est":"User Count",
    "userdays_est":"User Days"}
```

## Load dependencies¶

Import code from other jupyter notebooks, synced to \*.py with jupytext:

In [4]:

```
import sys
from pathlib import Path
module_path = str(Path.cwd().parents[0] / "py")
if module_path not in sys.path:
    sys.path.append(module_path)
```

In [5]:

```
from modules import preparations
preparations.init_imports()
WEB_DRIVER = preparations.load_chromedriver()
```

```
[WDM] - Downloading: 100%|██████████| 6.96M/6.96M [00:00<00:00, 37.9MB/s]
```

```
Chromedriver loaded. Svg output enabled.
```

**Import visualization notebook**

We're going to use many methods and the parameters defined in the previous notebook. These are imported form the jupytext converted python script file:

In [6]:

```
from _02_visualization import *
```

Activate autoreload of changed python files:

In [7]:

```
%load_ext autoreload
%autoreload 2
```

# Create Chi-maps¶

## Calculate Normalization Factor¶

To find regions where sunset or sunrise reactions are over or underrepresented, we first have to normalize our input data. The normalization of the observed data in regards to the expected data is necessary to account for their different data volumes. This is achieved by calculating their overall ratio. This ratio is then multiplied with the calculated chi value for each of the 65'342 bins to get a normalized chi map.

In [8]:

```
def calc_norm(
    grid_expected: gp.GeoDataFrame,
    grid_observed: gp.GeoDataFrame,
    chi_column: str = CHI_COLUMN):
    """Fetch the number of data points for the observed and 
    expected dataset by the relevant column
    and calculate the normalisation value
    """
    v_expected = grid_expected[chi_column].sum()
    v_observed = grid_observed[chi_column].sum()
    norm_val = (v_expected / v_observed)
    return norm_val
```

**Load data**

Load two files: expected ("total") and observed (e.g. "sunset", "sunrise" reactions).

In [9]:

```
grid_observed = grid_agg_fromcsv(
    OUTPUT / f"csv{km_size_str}" / "flickr_sunset_est.csv")

grid_expected = grid_agg_fromcsv(
    OUTPUT / f"csv{km_size_str}" / "flickr_all_est.csv")
```

In [10]:

```
grid_observed[grid_observed["postcount_est"]>100].head()
```

Out[10]:

|  |  | geometry | postcount\_est | usercount\_est | userdays\_est |
| --- | --- | --- | --- | --- | --- |
| xbin | ybin |  |  |  |  |
| -15540096 | -2220048 | POLYGON ((-15540096.000 -2220048.000, -1544009... | 254 | 35 | 63 |
| -15340096 | 2779952 | POLYGON ((-15340096.000 2779952.000, -15240096... | 2946 | 657 | 1036 |
|  | 2679952 | POLYGON ((-15340096.000 2679952.000, -15240096... | 811 | 233 | 313 |
|  | -2520048 | POLYGON ((-15340096.000 -2520048.000, -1524009... | 494 | 95 | 154 |
| -15240096 | 2779952 | POLYGON ((-15240096.000 2779952.000, -15140096... | 560 | 203 | 285 |

**Calculate normalization value**

In [11]:

```
norm_val = calc_norm(grid_expected, grid_observed)
print(norm_val)
```

```
17.455601895863786
```

### Merge dataframes¶

For easier calculation, we are loading data into a single GeoDataFrame by renaming expected column accordingly and then applying a merge operation.

In [12]:

```
rename_expected = {
    'postcount_est':'postcount_est_expected',
    'usercount_est':'usercount_est_expected',
    'userdays_est':'userdays_est_expected',
    }
grid_expected.rename(
    columns=rename_expected,
    inplace=True)
```

Merge expected and obsered GeoDataFrames based on index

In [13]:

```
merge_cols = ['postcount_est', 'usercount_est', 'userdays_est']
grid_expected_observed = grid_expected.merge(
    grid_observed[merge_cols],
    left_index=True, right_index=True)
```

Preview merged values

In [14]:

```
grid_expected_observed[
    grid_expected_observed['usercount_est_expected']>100].drop(['geometry'], axis = 1).head()
```

Out[14]:

|  |  | postcount\_est\_expected | usercount\_est\_expected | userdays\_est\_expected | postcount\_est | usercount\_est | userdays\_est |
| --- | --- | --- | --- | --- | --- | --- | --- |
| xbin | ybin |  |  |  |  |  |  |
| -17040096 | -1620048 | 2980 | 147 | 389 | 95 | 27 | 42 |
| -16940096 | -1620048 | 4235 | 277 | 746 | 47 | 17 | 24 |
|  | -1720048 | 2811 | 168 | 359 | 11 | 9 | 10 |
| -16840096 | -1720048 | 3714 | 136 | 473 | 9 | 7 | 7 |
|  | -2520048 | 4097 | 192 | 587 | 55 | 14 | 27 |

### Calculate the Chi-value¶

The chi value functions as a measure to estimate if the observed value is statistically under- or overrepresented in regards to the distribution of the expected value. In our case this chi value allows us to map regions which show statistically more or fewer mentions of the terms sunset or sunrise. This step is necessary given the fact that if people simply post more in a given region, the likeliness of the appearance of our terms of interest also increases which might not be significant.

In [15]:

```
def chi_calc(x_observed: float, x_expected: float, x_normalized: float) -> pd.Series:
    """Apply chi calculation based on observed (normalized) and expected value"""
    value_observed_normalised = x_observed * x_normalized
    a = value_observed_normalised - x_expected
    b = math.sqrt(x_expected)
    # native division with division by zero protection
    chi_value = a / b if b else 0
    # chi_value = a.divide(b.replace(0, np.nan)).fillna(0)
    # use np.divide to prevent division by zero error
    # will return 0 for expected == zero
    # chi_value = np.divide(a, b, out=np.zeros_like(a), where=b!=0)
    return chi_value
    
def apply_chi_calc(
        grid: gp.GeoDataFrame, norm_val: float,
        chi_column: str = CHI_COLUMN, chi_crit_val: float = CHI_CRIT_VAL):
    """Calculate chi-values based on two GeoDataFrames (expected and observed values)
    and return new grid with results"""
    # lambda: apply function chi_calc() to each item
    grid['chi_value'] = grid.apply(
        lambda x: chi_calc(
           x[chi_column],
           x[f'{chi_column}_expected'],
           norm_val),
        axis=1)
    # add significant column, default False
    grid['significant'] = False
    # calculate significance for both negative and positive chi_values
    grid.loc[np.abs(grid['chi_value'])>chi_crit_val, 'significant'] = True
```

**Apply calculation**

In [16]:

```
%%time
apply_chi_calc(
    grid=grid_expected_observed,
    norm_val=norm_val)
```

```
CPU times: user 984 ms, sys: 7.68 ms, total: 992 ms
Wall time: 991 ms
```

### Distribution of chi-values¶

A quick glance at the distribution of the chi-values tells us that most grid cells have a very low chi-value, wheras the chi-value peaks quite high in a few grid cells:

In [17]:

```
chi_series=pd.Series(grid_expected_observed['chi_value'].astype(float))
```

In [18]:

```
chi_series.plot.hist(bins=50)
```

Out[18]:

```
<AxesSubplot:ylabel='Frequency'>
```

## Over- and underrepresentation plotting function¶

Unfortunately, the pyviz (holoviews, bokeh, geoviews etc.) framework requires quite some  
customization to be able to plot two colormaps in one map. The methods below is a repeat of  
the process from the previous Notebook, with modifications for a two-color range colormap,  
for representing over- and underrepresentation.

Configure what should be shown on mouse hover tooltip:

In [19]:

```
hover_items = { 
    'Post Count (est)':'postcount_est',
    'User Count (est)':'usercount_est',
    'User Days (est)':'userdays_est',}
hover_items_chi = {
    f'Total {METRIC_NAME_REF[CHI_COLUMN]}':f'{CHI_COLUMN}_expected',
    'Chi-value':'chi_value',
    'Chi-significant':'significant',
    'Label Cat':'chi_value_cat_label'}
hover_items.update(hover_items_chi)
```

Configure diverging labels of the colormap

In [20]:

```
def create_diverging_labels(
        div_bounds: List[List[float]],
        add_nodata_label: str = None,
        add_notopicdata_label: str = None,
        mask_nonsignificant: bool = None,
        true_negative: bool = True,
        offset: int = 0) -> Dict[int, str]:
    """Create labels for diverging cmap, add special categories to cmap"""
    label_dict = {}
    spare_cats = 0
    if add_notopicdata_label:
        spare_cats += 1
    if add_nodata_label:
        spare_cats += 1
    for ix, bounds in enumerate(div_bounds):
        ldict = {}
        for idx, value_str in enumerate(bounds):
            if ix == 1:
                neg = "" 
                if true_negative and value_str != "0":
                    # get "minus" range labels
                    neg = "-"
                    # invert cat number
                ldict[0-idx-offset] = f'{neg}{value_str}'
                continue
            ldict[idx+spare_cats+offset] = value_str
        label_dict.update(ldict)
    if add_notopicdata_label:
        label_dict[spare_cats] = "Not Significant" 
    if offset:
        label_dict[offset] = "No Data"
        label_dict[0] = "<0"
    # add symbols '≤' or '≥'
    symbol_plus = '≥'
    symbol_minus = '≥'
    if true_negative:
        symbol_minus = '≤'
    if int(label_dict[spare_cats+offset]) == 0:
        symbol_plus = '>'
    label_dict[spare_cats+offset] = f'{symbol_plus}{label_dict[spare_cats+offset]}'
    if spare_cats > 0 and int(label_dict[0-offset]) == 0:
        symbol_minus = '<'
        if true_negative:
            symbol_minus = '<'
    label_dict[0-offset] = f'{symbol_minus}{label_dict[0-offset]}'
    return label_dict
```

Method for assigning special categories to data.

In [21]:

```
def assign_special_categories(
        grid: gp.GeoDataFrame, series_plus, series_minus,
        metric, add_nodata_label,
        mask_nonsignificant, add_notopicdata_label,
        add_underrepresented_label):
    """Assign special categories to _cat column"""
    plus_mask = grid.index.isin(series_plus.index)
    minus_mask = grid.index.isin(series_minus.index)
    offset = 0
    if add_notopicdata_label:
        offset = 1
    if add_nodata_label:
        # render data values as transparent
        # this is category "0" in the legend
        grid.loc[
            ~((plus_mask) | (minus_mask)),
            f'{metric}_cat'] = np.nan
    if add_underrepresented_label:
        mask = (grid["underrepresented"] == "sunset")
        grid.loc[mask, f'{metric}_cat'] = str(offset+1)
        mask = (grid["underrepresented"] == "sunrise")
        grid.loc[mask, f'{metric}_cat'] = str(-offset)
    if mask_nonsignificant:
        # also set none-significant values to nan
        # to not affect classification (cat = nan)
        mask = (grid.significant == False)
        if add_underrepresented_label:
            mask = mask & (grid["underrepresented"].isna())
        grid.loc[
            mask,
            f'{metric}_cat'] = np.nan
    if add_notopicdata_label:
        # render where data exists, 
        # but no cat label so far, as grey
        mask = (grid["usercount_est_expected"] >= 1) & \
            (grid[f'{metric}_cat'].isna())
        grid.loc[
            mask,
            f'{metric}_cat'] = '1'
```

In [22]:

```
from typing import Union
def cast_to_int(f: float)-> Union[int, float]:
    """Return int if float is whole number
    Note: Fixes a display bug in Bokeh (Holoviews #3583), 
    where tick locations based on floats that are whole 
    numbers (e.g. 10.0) do not get plotted.
    """
    if f.is_integer():
        return int(f)
    return f

def update_tick_positions(label_dict):
    """(Ugly) method to adjust tick positions, due to one additional
    label shown in legend (no_data_label).
    
    Notes:
        There are x number of labels, but the length 
        of categories mapped is x-1, due to one
        additional entry only shown in the cmap legend. As a result,
        x labels need to fit into x-1 vertical space, meaning
        that the y-size of color-bins is < 1. The position
        of ticks must be adjusted to fit the new reduced y-size of
        bins. TODO: Simplify code.
    """
    label_dict_new = {}
    cat_count = len(label_dict)-1
    cat_height = cat_count/(cat_count+1)
    loc_offsets = []
    _min = min(label_dict.keys())
    _max = max(label_dict.keys())
    max_cats = int(max(np.abs(_min), _max))
    for cat_nr in range(0, max_cats+1):
        if cat_nr == 0:
            loc_offset = cat_height/2
            label_dict_new[loc_offset] = label_dict[1]  
            label_dict_new[-loc_offset] = label_dict[0]
            continue
        else:
            loc_offset += cat_height
        if cat_nr <= _max-1:
            if cat_nr != np.abs(_max)-1:
                tick_lock = loc_offset
                tick_lock = cast_to_int(tick_lock)
            else:
                tick_lock = max_cats-1
            label_dict_new[tick_lock] = label_dict[cat_nr+1]
        if cat_nr <= np.abs(_min):
            if cat_nr != np.abs(_min):
                tick_lock = loc_offset
                tick_lock = cast_to_int(tick_lock)
            else:
                tick_lock = int(np.abs(_min))
            label_dict_new[-tick_lock] = label_dict[-cat_nr]
    return label_dict_new
```

Main methods to create a diverging image layer,  
combined from `series_plus` and `series_minus`.

**Note:** To map/combine two classes/colormaps,  
the second series label category indices are internally  
prepended with a minus sign (-). This minus sign can  
be used to represent ranges other than over- or  
underrepresentation, such as sunset/sunrise or  
instagram/flickr. However, the actual values  
bounds/scheme\_breaks that are classified are left  
unmodified, e.g. remain positive or negative in case of  
over- or underrepresentation.

In [23]:

```
def compile_diverging_image_layer(
        grid: gp.GeoDataFrame, series_plus: pd.Series,
        series_minus: pd.Series,
        metric: str = "chi_value", responsive: bool = None,
        hover_items: Dict[str, str] = hover_items,
        mask_nonsignificant: bool = False,
        add_notopicdata_label: str = None,
        scheme: str = "HeadTailBreaks",
        cmaps_diverging: Tuple[str] = ("OrRd", "Blues"),
        add_nodata_label: str = "#FFFFFF",
        true_negative: bool = True):
    """Modified function to get/combine diverging image layer
    
    Additional Args:
        series_plus: Series of values to show on plus y range cmap
        series_minus: Series of values to show on minus y range cmap
        cmaps_diverging: Tuple with plus and minus cmap reference
    """
    ##stats = {}
    div_labels: List[Dict[int, str]] = []
    div_cmaps: List[List[str]] = []
    cat_counts: List[int] = []
    div_bounds: List[List[float]] = []
    spare_cats = 0
    plus_offset = 0
    offset = 0
    if "underrepresented" in grid.columns:
        # used in 04_combine.ipynb
        offset = 1
    for ix, series_nan in enumerate([series_plus, series_minus]):
        # classify values for both series
        cmap_name = cmaps_diverging[ix]
        bounds, scheme_breaks = classify_data(
            values_series=series_nan, scheme=scheme)
        div_bounds.append(bounds)
        # assign categories column
        cat_series = scheme_breaks.find_bin(
            np.abs(series_nan.values))
        cat_count = scheme_breaks.k
        color_count = scheme_breaks.k + offset
        cmap_list = get_cmap_list(
            cmap_name, length_n=color_count)
        if ix == 0:
            if add_notopicdata_label:
                # add grey color
                plus_offset = 1
                prepend_color(
                    cmap_list=cmap_list, color_hex=add_notopicdata_label)
            if add_nodata_label:
                # offset
                spare_cats = 1
                prepend_color(
                    cmap_list=cmap_list, color_hex=add_nodata_label)
            cat_count += (offset+plus_offset)
            # nodata label as explicit category in legend
            # values will not be rendered on map (cat = nan):
            # increment cat count, but not series
            cat_series += (offset+plus_offset+spare_cats)
        if ix == 1:
            cat_count += offset
            # cat labels always prepended with minus sign (-)
            cat_series = np.negative(cat_series)
            # offset
            cat_series -= (1+offset)
        cat_counts.append(cat_count)
        div_cmaps.append(cmap_list)
        # assign categories
        grid.loc[series_nan.index, f'{metric}_cat'] = cat_series.astype(str)
    # general application:
    # assign special cat labels to data
    assign_special_categories(
        grid=grid, series_plus=series_plus, series_minus=series_minus,
        metric=metric, add_notopicdata_label=add_notopicdata_label,
        add_underrepresented_label=offset,
        add_nodata_label=add_nodata_label, mask_nonsignificant=mask_nonsignificant)
    # allow label cat to be shown on hover
    grid.loc[grid.index, f"{metric}_cat_label"] = grid[f"{metric}_cat"]
    hover_items['Label Cat'] = 'chi_value_cat_label'
    # special categories
    kwargs = {
        "mask_nonsignificant":mask_nonsignificant,
        "add_nodata_label":add_nodata_label,
        "add_notopicdata_label":add_notopicdata_label,
        "true_negative":true_negative,
        "offset":offset
    }
    label_dict = create_diverging_labels(
        div_bounds, **kwargs)
    # adjust tick positions, 
    # due to additional no_data_label
    # shown in legend only
    if add_nodata_label:
        label_dict = update_tick_positions(label_dict)
    # reverse colors of minus cmap
    div_cmaps[1].reverse()
    # combine cmaps
    cmap_nodata_list = div_cmaps[1] + div_cmaps[0]
    cmap = colors.ListedColormap(cmap_nodata_list)
    # create gv.image layer from gdf
    img_grid = convert_gdf_to_gvimage(
            grid=grid,
            metric=metric, cat_min=-cat_counts[1],
            cat_max=cat_counts[0],
            hover_items=hover_items)
    image_layer = apply_layer_opts(
        img_grid=img_grid, cmap=cmap, label_dict=label_dict,
        responsive=responsive, hover_items=hover_items)
    return image_layer
```

High level plotting method

In [24]:

```
def plot_diverging(grid: gp.GeoDataFrame, title: str,
    hover_items: Dict[str, str] = {
        'Post Count (estimated)':'postcount_est', 
        'User Count (estimated)':'usercount_est',
        'User Days (estimated)':'userdays_est'},
    mask_nonsignificant: bool = False,
    scheme: str = "HeadTailBreaks",
    cmaps_diverging: Tuple[str] = ("OrRd", "Blues"),
    store_html: str = None,
    plot: Optional[bool] = True,
    output: Optional[str] = OUTPUT,
    nodata_color: str = "#FFFFFF",
    notopicdata_color: str = None,
    true_negative: bool = True) -> gv.Overlay:
    """Plot interactive map with holoviews/geoviews renderer

    Args:
        grid: A geopandas geodataframe with indexes x and y 
            (projected coordinates) and aggregate metric column
        metric: target column for aggregate. Default: postcount.
        store_html: Provide a name to store figure as interactive HTML. If 
            WEB_DRIVER is set, will also export to svg.
        title: Title of the map
        cmaps_diverging: Tuple for colormaps to use.
        hover_items: additional items to show on hover
        mask_nonsignificant: transparent bins if significant column == False
        scheme: The classification scheme to use. Default "HeadTailBreaks".
        cmap: The colormap to use. Default "OrRd".
        plot: Prepare gv-layers to be plotted in notebook.
        true_negative: Whether "minus" values should show "-" in legend.
    """
     # check if all additional items are available
    for key, item in list(hover_items.items()):
        if item not in grid.columns:
            hover_items.pop(key)
    grid_plot = grid.copy()
    # chi layer opts
    base_kwargs = {
        "mask_nonsignificant":mask_nonsignificant,
        "metric":"chi_value",
        "grid":grid_plot,
    }
    # classify based on positive and negative chi
    series_plus = mask_series(
        mask_negative=True,
        **base_kwargs)
    series_minus = mask_series(
        mask_positive=True, 
        **base_kwargs)
    # global plotting options for value layer
    layer_opts = {
        "series_plus":series_plus,
        "series_minus":series_minus,
        "responsive":False,
        "scheme":scheme,
        "hover_items":hover_items,
        "cmaps_diverging":cmaps_diverging,
        "add_nodata_label":nodata_color,
        "add_notopicdata_label":notopicdata_color,
        "true_negative":true_negative,
    }    
    # global plotting options for all layers (gv.Overlay)
    gv_opts = {
        "bgcolor":None,
        # "global_extent":True,
        "projection":crs.Mollweide(),
        "responsive":False,
        "data_aspect":1, # maintain fixed aspect ratio during responsive resize
        "hooks":[set_active_tool],
        "title":title
    }
    # create layers for highlighting outliers
    selmax_points = grid.nlargest(5, "chi_value").centroid
    selmax_layer = tools.series_to_point(selmax_points) 
    selmax_labels = tools.series_to_label(selmax_points)
    selmin_points = grid.nsmallest(5, "chi_value").centroid
    selmin_layer = tools.series_to_point(selmin_points)
    selmin_labels = tools.series_to_label(selmin_points)
    annotation_layers = get_annotation_layer(
        sel_layer1=selmax_layer, sel_labels1=selmax_labels,
        sel_layer2=selmin_layer, sel_labels2=selmin_labels)
    # plot responsive (html) or non-responsive (interactive)
    if plot:
        # get classified xarray gv image layer
        image_layer = compile_diverging_image_layer(
            **layer_opts, **base_kwargs)
        gv_layers = combine_gv_layers(
            image_layer, fill_color=nodata_color, alpha=0.5,
            additional_layers=annotation_layers)
    if store_html:
        layer_opts["responsive"] = True
        image_layer = compile_diverging_image_layer(
            **layer_opts, **base_kwargs)
        responsive_gv_layers = combine_gv_layers(
            image_layer, fill_color=nodata_color, alpha=0.5)
        gv_opts["responsive"] = True
        export_layers = responsive_gv_layers.opts(**gv_opts)
        hv.save(
            export_layers,
            output / f"html{km_size_str}" / f'{store_html}.html', backend='bokeh')
        if WEB_DRIVER:
            # store also as svg
            p =  hv.render(export_layers, backend='bokeh')
            p.output_backend = "svg"
            export_svgs(
                p, filename=output / f"svg{km_size_str}" / f'{store_html}.svg',
                webdriver=WEB_DRIVER)
    if not plot:
        return
    gv_opts["responsive"] = False
    return gv_layers.opts(**gv_opts)
```

## Plot results to interactive map¶

Set global config options:

- map bin colors to `chi_value`
- show `hover_items` on hover

In [25]:

```
kwargs = {
    "hover_items":hover_items,
    "cmaps_diverging":("OrRd", "Blues")
}
```

### Output with non-significant values¶

If we plot chi-values without specific treatment of non-significant values, the map looks pretty noisy.

In [26]:

```
pd.set_option('display.max_colwidth', 500)
gv_plot = plot_diverging(
    grid_expected_observed,
    title=f'Chi values (over- and underrepresentation): Flickr "Sunset" {METRIC_NAME_REF[CHI_COLUMN]} (estimated) per {km_size:.0f} km grid, 2007-2018',
    mask_nonsignificant=False, **kwargs)
gv_plot
```

Out[26]:

**Percentage of bins with none-significant values?**

In [27]:

```
total = len(grid_expected_observed[f"{CHI_COLUMN}_expected"].dropna())
total_data = total - len(grid_expected_observed.query(f'{CHI_COLUMN}_expected == 0'))
significant = len(grid_expected_observed[grid_expected_observed["significant"] == True])
print(f"Chi-value is significant for {significant} of {total_data} non-empty bins ({significant/(total_data/100):.2f}%)")
```

```
Chi-value is significant for 7230 of 26859 non-empty bins (26.92%)
```

### Remove non-significant values¶

To remove noise, use `mask_nonsignificant=True`, which renders non-significant chi-values as transparent:

In [28]:

```
kwargs["mask_nonsignificant"] = True
```

In [29]:

```
gv_plot = plot_diverging(
    grid_expected_observed,
    title=f'Chi-value (overrepresentation): Flickr "Sunset" {METRIC_NAME_REF[CHI_COLUMN]} (estimated) per {km_size:.0f} km grid, 2007-2018', **kwargs)
gv_plot
```

Out[29]:

### Use different Classification Scheme¶

When not showing non-significant values, it makes sense to increase visibility of lower chi-values by applying "NaturalBreaks" classification instead of "HeadTailBreaks"

In [30]:

```
gv_plot = plot_diverging(
    grid_expected_observed, title=f'Chi-value: Flickr "Sunset" {METRIC_NAME_REF[CHI_COLUMN]} (estimated) per {km_size:.0f} km grid, 2007-2018',
    scheme="NaturalBreaks", store_html=f"sunset_flickr_chi_{CHI_COLUMN.removesuffix('_est')}_naturalbreaks", **kwargs)
gv_plot
```

Out[30]:

Combine into single method

In [31]:

```
def get_chimap_fromcsv(
        csv_expected: str = "flickr_all_est.csv", 
        csv_observed: str = "flickr_sunset_est.csv",
        output: Optional[Path] = OUTPUT,
        km_size_str: Optional[str] = km_size_str,
        chi_column: Optional[str] = CHI_COLUMN) -> gp.GeoDataFrame:
    """Restore grid-data from CSV for expected and observed values
    and calculate chi-values (over- and underrepresentation).
    """
    columns = ["xbin", "ybin", "postcount_est", "usercount_est", "userdays_est"]
    grid_observed = grid_agg_fromcsv(
        output / f"csv{km_size_str}" / csv_observed)
    grid_expected = grid_agg_fromcsv(
        output / f"csv{km_size_str}" / csv_expected)
    # calculate normalization value based on global average
    norm_val = calc_norm(
        grid_expected, grid_observed, chi_column=chi_column)
    # merge data
    grid_expected.rename(
        columns={
            'usercount_est':'usercount_est_expected',
            'postcount_est':'postcount_est_expected',
            'userdays_est':'userdays_est_expected'},
        inplace=True)
    merge_cols = ['postcount_est', 'usercount_est', 'userdays_est']
    grid_expected_observed = grid_expected.merge(
        grid_observed[merge_cols],
        left_index=True, right_index=True)
    # calculate chi
    apply_chi_calc(
        grid=grid_expected_observed,
        norm_val=norm_val,
        chi_column=chi_column)
    return grid_expected_observed
```

**Repeat for sunrise:**

In [32]:

```
grid_expected_observed = get_chimap_fromcsv(
    csv_expected="flickr_all_est.csv",
    csv_observed="flickr_sunrise_est.csv",
    chi_column=CHI_COLUMN)
```

In [33]:

```
gv_plot = plot_diverging(
    grid_expected_observed, title=f'Chi-value: Flickr "Sunrise" {METRIC_NAME_REF[CHI_COLUMN]} (estimated) per {km_size:.0f} km grid, 2007-2018',
    scheme="NaturalBreaks", store_html=f"sunrise_flickr_chi_{CHI_COLUMN.removesuffix('_est')}_naturalbreaks", **kwargs)
gv_plot
```

Out[33]:

**Repeat for Instagram**

In [34]:

```
csv_kwargs = {
    # "csv_expected":"instagram_sunsetsunrise_est.csv",
    "csv_expected":"instagram_random_est.csv",
    "csv_observed":"instagram_sunrise_est.csv",
    "chi_column":CHI_COLUMN
}
grid_expected_observed = get_chimap_fromcsv(**csv_kwargs)
```

In [35]:

```
kwargs["scheme"] ="NaturalBreaks"
gv_plot = plot_diverging(
    grid_expected_observed, title=f'Chi-value: Instagram "Sunrise" {METRIC_NAME_REF[CHI_COLUMN]} (estimated) per {km_size:.0f} km grid, Aug-Dec 2017',
    store_html=f"sunrise_instagram_chi_{CHI_COLUMN.removesuffix('_est')}_naturalbreaks", **kwargs)
gv_plot
```

Out[35]:

In [36]:

```
csv_kwargs["csv_observed"] = "instagram_sunset_est.csv"
grid_expected_observed = get_chimap_fromcsv(**csv_kwargs)
```

In [37]:

```
gv_plot = plot_diverging(
    grid_expected_observed, title=f'Chi-value: Instagram "Sunset" {METRIC_NAME_REF[CHI_COLUMN]} (estimated) per {km_size:.0f} km grid, Aug-Dec 2017',
    store_html=f"sunset_instagram_chi_{CHI_COLUMN.removesuffix('_est')}_naturalbreaks",**kwargs)
gv_plot
```

Out[37]:

**Use Flickr expected to calculate Instagram sunrise/sunset chi**

In [38]:

```
csv_kwargs["csv_expected"] = "flickr_all_est.csv"
grid_expected_observed = get_chimap_fromcsv(**csv_kwargs)
```

In [ ]:

```
gv_plot = plot_diverging(
    grid_expected_observed,
    title=f'Chi-value (Flickr expected): Instagram "Sunset" {METRIC_NAME_REF[CHI_COLUMN]} (estimated) per {km_size:.0f} km grid, Aug-Dec 2017',
    store_html=f"sunset_instagram_chi_fe_{CHI_COLUMN.removesuffix('_est')}_naturalbreaks", **kwargs)
gv_plot
```

In [40]:

```
csv_kwargs["csv_observed"] = "instagram_sunrise_est.csv"
grid_expected_observed = get_chimap_fromcsv(**csv_kwargs)
```

In [ ]:

```
gv_plot = plot_diverging(
    grid_expected_observed, 
    title=f'Chi-value (Flickr expected): Instagram "Sunrise" {METRIC_NAME_REF[CHI_COLUMN]} (estimated) per {km_size:.0f} km grid, Aug-Dec 2017',
    store_html=f"sunrise_instagram_chi_fe_{CHI_COLUMN.removesuffix('_est')}_naturalbreaks", **kwargs)
gv_plot
```

# Create notebook HTML¶

In [38]:

```
!jupyter nbconvert --to html_toc \
    --output-dir=../out/html{km_size_str} ./03_chimaps.ipynb \
    --template=../nbconvert.tpl \
    --ExtractOutputPreprocessor.enabled=False >&- 2>&- # create single output file
```

Copy new HTML file to resource folder

In [2]:

```
!cp ../out/html{km_size_str}/03_chimaps.html ../resources/html/
```

In [ ]:

```

```
