# Supplementary material for: From sunrise to sunset: Exploring landscape preference through global reactions to ephemeral events captured in georeferenced social media
Source: PLoS One. 2023 Feb 22;18(2):e0280423. doi: 10.1371/journal.pone.0280423 (PMC9946259; doi:10.1371/journal.pone.0280423)
Supplement: S4 File — (HTML) [file pone.0280423.s004.html]

04\_combine


# Interpretation: Combine results¶

*Alexander Dunkel, TU Dresden, Institute of Cartography; Maximilian Hartmann, Universität Zürich (UZH), Geocomputation*

---

•••

Out[1]:

Last updated: Jan-17-2023, Carto-Lab Docker Version 0.9.0

# Introduction¶

This is the fourth notebook in a series of nine notebooks:

1. the grid aggregation notebook (01\_gridagg.ipynb) is used to aggregate data from HLL sets at GeoHash 5 to a 100x100km grid
2. the visualization notebook (02\_visualization.ipynb) is used to create interactive maps, with additional information shown on hover
3. the chimaps notebook (03\_chimaps.ipynb) shows how to compute the chi square test per bin and event (sunset/sunrise).
4. the results notebook (04\_combine.ipynb) shows how to combine results from sunset/sunrise into a single interactive map.
5. Notebooks 5 to 9 are used for creating additional graphics and statistics.

**Merge sunset and sunrise chi values to a single map**

Using a diverging colormap, we here combine sunset & sunrise positive chi values only, to increase information content per map.

For merging sunset and sunrise chi, the following considerations apply:

- only use positive chi values for sunset and sunrise (overrepresentation)
- snap underrepresentation for both sunset/sunrise to a single color label
- normalize chi values for sunset and sunrise (to 1-1000 range), as a means to allow comparison
- if both sunset and sunrise chi\_value have the same significance (both False or both True): use the larger value of the two
- combine in a single map, based two colormaps (blue = sunrise, reds = sunset), merged as a diverging colormap

In this notebook, the negative category range (blue colors) is used to represent "sunrise" and the positive category range (red) is used to represent "sunset", on a diverging color map. Do not confuse with underrepresentation, which is reduced/mapped to a single category/color on these map.

## Load dependencies¶

Import code from other jupyter notebooks, synced to \*.py with jupytext:

In [2]:

```
import sys
from pathlib import Path
module_path = str(Path.cwd().parents[0] / "py")
if module_path not in sys.path:
    sys.path.append(module_path)
# import all previous chained notebooks
from _03_chimaps import *
from modules import preparations
```

```
Chromedriver loaded. Svg output enabled.
```

# Parameters¶

Override which metric is used to calculate chi-values. Default is `usercount_est`.

In [3]:

```
# CHI_COLUMN = "postcount_est"
# CHI_COLUMN = "userdays_est" 
# CHI_COLUMN = "usercount_est"
```

In [4]:

```
display(CHI_COLUMN)
```

```
'usercount_est'
```

# Additional preparations¶

Activate autoreload of changed python files:

In [5]:

```
%load_ext autoreload
%autoreload 2
```

# Merge Sunset & Sunrise Chi¶

## Merge grids¶

Load sunset and sunrise grid from CSV:

In [6]:

```
grid_sunset = get_chimap_fromcsv(
        csv_expected="flickr_all_est.csv",
        csv_observed="flickr_sunset_est.csv",
        chi_column=CHI_COLUMN
        )
grid_sunrise = get_chimap_fromcsv(
        csv_expected="flickr_all_est.csv",
        csv_observed="flickr_sunrise_est.csv",
        chi_column=CHI_COLUMN
        )
```

Normalize chi\_values to 1 to 1000 range for comparison.

In [7]:

```
def norm_series(
        col_series: pd.Series, range_min: Optional[int] = 1, range_max: Optional[int] = 1000) -> np.ndarray:
    """Normalize (interpolate) Series to new range min-max"""
    return np.interp(
        col_series, (col_series.min(), col_series.max()), (range_min, range_max))
    
def norm_col_diverging(
    df: gp.GeoDataFrame, col_name: Optional[str] = "chi_value",
    range_min: Optional[int] = 1, range_max: Optional[int] = 1000,
    mask_minus: Optional[pd.Series] = None, mask_plus: Optional[pd.Series] = None):
    """Normalize positive and negative slice of df[col_name] to range (-)min to (-)max"""
    if mask_minus is None:
        mask_minus = df[col_name] < 0
    if mask_plus is None:
        mask_plus = df[col_name] > 0
    for ix, mask in enumerate([mask_plus, mask_minus]):
        col_series  = df.loc[mask, col_name]
        if ix == 1:
            # -1,-1000
            df.loc[mask, col_name] = norm_series(
                col_series, np.negative(range_max), np.negative(range_min))
            continue
        df.loc[mask, col_name] = norm_series(
            col_series, range_min, range_max)
```

Apply normalization

In [8]:

```
for grid in [grid_sunrise, grid_sunset]:
    norm_col_diverging(grid)
```

Validate

In [9]:

```
print(grid_sunset["chi_value"].max())
print(grid_sunset.loc[grid_sunset["chi_value"] > 0, "chi_value"].min())
print(grid_sunset.loc[grid_sunset["chi_value"] < 0, "chi_value"].max())
print(grid_sunset["chi_value"].min())
```

```
1000.0
1.0
-1.0
-1000.0
```

Rename cols, so that both grids can be merged:

In [10]:

```
def grid_rename_cols(grid: gp.GeoDataFrame, colmap: Dict[str, str]):
    """Rename columns in gdf according to column mapping (colmap)"""
    grid.rename(columns=colmap, inplace=True)
```

Merge grids:

- Merge only some cols, excluding e.g. polygons and index
- rename columns

In [11]:

```
def merge_df(df_a: pd.DataFrame, df_b: pd.DataFrame, merge_cols: List[str]) -> pd.DataFrame:
    """Merge two DataFrames based on index, merge only
    columns specified in merge_cols. Returns a new DataFrame"""
    df_merged = df_a.merge(
        df_b[merge_cols],
        left_index=True, right_index=True)
    return df_merged
```

Specific syntax to merge grid columns with two topics (e.g. sunset/sunrise)

In [12]:

```
def merge_grids(
        grid_plus: gp.GeoDataFrame, grid_minus: gp.GeoDataFrame,
        t_plus: str = "sunset", t_minus: str = "sunrise") -> gp.GeoDataFrame:
    """Merge two GeoDataFrame with two topics t_minus and t_plus,
    rename columns on merge"""
    colmap_plus = {
        'chi_value':f'chi_value_{t_plus}',
        'significant':f'significant_{t_plus}',
        'usercount_est':f'usercount_est_{t_plus}',
        'postcount_est':f'postcount_est_{t_plus}',
        'userdays_est':f'userdays_est_{t_plus}'}
    colmap_minus = {
        'chi_value':f'chi_value_{t_minus}',
        'significant':f'significant_{t_minus}',
        'usercount_est':f'usercount_est_{t_minus}',
        'postcount_est':f'postcount_est_{t_minus}',
        'userdays_est':f'userdays_est_{t_minus}'}
    # merge sunset & sunrise chi
    grid_rename_cols(grid_plus, colmap_plus)
    grid_rename_cols(grid_minus, colmap_minus)
    merge_cols = [
        f'chi_value_{t_minus}', f'significant_{t_minus}',
        f'usercount_est_{t_minus}', f'postcount_est_{t_minus}',
        f'userdays_est_{t_minus}']
    grid = merge_df(grid_plus, grid_minus, merge_cols)
    return grid
```

In [13]:

```
grid = merge_grids(grid_sunset, grid_sunrise)
```

**Create chi\_value and significant column to store merged values**

Comparison step, according the rules defined in the introduction.

To later distinguish between chi\_values from sunset and sunrise,  
use negative values for sunrise and positive for sunset.

The syntax here is quite complex because a lot of values/combinations  
need to be solved during aggregation. Consider the result with a grain of salt.

- if both chi values (sunset & sunrise) are positive,
  - select significant value, if other is not significant
  - or select the larger value, if significance for both is the same
  - if both are equal, select the first
- if either one of the two chi values is positive,
  use this one
- for both underrepresented: add a column with a reference  
  of the larger of the two topics

In [14]:

```
def merge_chi_value(grid: gp.GeoDataFrame, t_minus: str = "sunrise", t_plus: str = "sunset", metric: str = "chi_value", chi_column = CHI_COLUMN):
    """Merge positive chi value (overrepresentation) of two topics to single, new column. Join significance.
    
    Notes: t_minus topic values will be turned to negative values, 
       for mapping on diverging cmap. The procedure uses boolean indexing.
       Grid can be given as a slice of a larger dataframe (e.g. focus on positive values only)
    """
    # init columns
    grid[metric] = np.nan
    grid["significant"] = False
    # (1) --
    # get positive chi values grid slice (overrepresented)
    sel_mask = grid_slice(grid, t_minus, t_plus, metric, positive=True)
    gsel = grid[sel_mask]
    grid.loc[sel_mask, f'{metric}'] = np.where(
        (((gsel[f"significant_{t_minus}"] == False) & (gsel[f"significant_{t_plus}"] == True)) | # or
         ((gsel[f"significant_{t_minus}"] == gsel[f"significant_{t_plus}"]) & # and
          (gsel[f"{metric}_{t_plus}"] > gsel[f"{metric}_{t_minus}"]))),
        gsel[f"{metric}_{t_plus}"], # if True
        np.negative(gsel[f"{metric}_{t_minus}"]) # if False
        )
    # (2)--
    # special case: over- and underrepresentation present
    sel_mask = grid_slice(grid, t_minus, t_plus, metric, both=True)
    sel_mask_tplus = (sel_mask) & (grid[f"{metric}_{t_plus}"] > 0)
    gsel = grid[sel_mask_tplus]
    grid.loc[sel_mask_tplus, f'{metric}'] = gsel[f"{metric}_{t_plus}"]
    sel_mask_tminus = (sel_mask) & (grid[f"{metric}_{t_minus}"] > 0)
    gsel = grid[sel_mask_tminus]
    grid.loc[sel_mask_tminus, f'{metric}'] = np.negative(gsel[f"{metric}_{t_minus}"])
    # --
    # join significance
    mask_t_plus = (grid[f'{metric}'] > 0) & (grid[f"significant_{t_plus}"] == True)
    mask_t_minus =(grid[f'{metric}'] < 0) & (grid[f"significant_{t_minus}"] == True)
    grid.loc[mask_t_plus, 'significant'] = True
    grid.loc[mask_t_minus, 'significant'] = True
    # (3)--
    # last case: both underrepresented
    # join the larger of the two topic's 
    # underrepresented references to one column,
    sel_mask = grid_slice(grid, t_minus, t_plus, metric, negative=True)
    # only significant
    sel_mask = sel_mask \
        & ((grid[f"significant_{t_plus}"] == True) | (grid[f"significant_{t_minus}"] == True))
    # only where at least one metric is not null 
    # (e.g. usercount_est_sunrise or usercount_est_sunset)
    sel_mask = sel_mask & ((grid[f"{chi_column}_{t_minus}"] != 0) | (grid[f"{chi_column}_{t_plus}"] != 0))
    gsel = grid[sel_mask]
    grid.loc[sel_mask, 'underrepresented'] = np.where(
        (((gsel[f"significant_{t_minus}"] == False) & (gsel[f"significant_{t_plus}"] == True)) | # or
         ((gsel[f"significant_{t_minus}"] == gsel[f"significant_{t_plus}"]) & # and
          (gsel[f"{metric}_{t_plus}"] > gsel[f"{metric}_{t_minus}"]))),
        t_plus, # if True
        t_minus # if False
        )

def grid_slice(
        grid: gp.GeoDataFrame, t_minus: str = "sunrise", t_plus: str = "sunset",
        metric: str = "chi_value",
        positive: bool = None, negative: bool = None, both: bool = None) -> gp.GeoDataFrame:
    """Return positive or negative grid slices for selected topics"""
    if positive:
        plus_mask = grid[f"{metric}_{t_plus}"] > 0
        minus_mask = grid[f"{metric}_{t_minus}"] > 0
    elif negative:
        plus_mask = grid[f"{metric}_{t_plus}"] < 0
        minus_mask = grid[f"{metric}_{t_minus}"] < 0
    elif both:
        plus_mask = grid[f"{metric}_{t_plus}"] < 0
        minus_mask = grid[f"{metric}_{t_minus}"] > 0
    else:
        raise ValueError(
            "Provide either one of negative, positive, or both")
    if both:
        sel_mask = ((plus_mask) & (minus_mask)) | (~(plus_mask) & ~(minus_mask))
        return sel_mask
    sel_mask = (plus_mask) & (minus_mask)
    return sel_mask
```

Merge

In [15]:

```
merge_chi_value(grid)
```

Validation: both sunset and sunrise extreme values must be returned, which is -1000 (inverted) chi for sunrise and +1000 chi for sunset.

In [16]:

```
print(grid["chi_value"].max())
print(grid["chi_value"].min())
```

```
1000.0
-1000.0
```

In [17]:

```
grid.drop(['geometry'], axis = 1).sample(50).head(10)
```

Out[17]:

|  |  | postcount\_est\_expected | usercount\_est\_expected | userdays\_est\_expected | postcount\_est\_sunset | usercount\_est\_sunset | userdays\_est\_sunset | chi\_value\_sunset | significant\_sunset | chi\_value\_sunrise | significant\_sunrise | usercount\_est\_sunrise | postcount\_est\_sunrise | userdays\_est\_sunrise | chi\_value | significant | underrepresented |
| --- | --- | --- | --- | --- | --- | --- | --- | --- | --- | --- | --- | --- | --- | --- | --- | --- | --- |
| xbin | ybin |  |  |  |  |  |  |  |  |  |  |  |  |  |  |  |  |
| 6959904 | 1279952 | 0 | 0 | 0 | 0 | 0 | 0 | 0.000000 | False | 0.000000 | False | 0 | 0 | 0 | NaN | False | NaN |
| -8440096 | 79952 | 2 | 1 | 1 | 0 | 0 | 0 | -10.628659 | False | -7.156078 | False | 0 | 0 | 0 | NaN | False | NaN |
| 7259904 | -5820048 | 0 | 0 | 0 | 0 | 0 | 0 | 0.000000 | False | 0.000000 | False | 0 | 0 | 0 | NaN | False | NaN |
| -240096 | 4279952 | 2379 | 229 | 804 | 60 | 13 | 29 | -2.317064 | False | -49.564497 | True | 2 | 3 | 3 | NaN | False | sunrise |
| -17040096 | 4279952 | 0 | 0 | 0 | 0 | 0 | 0 | 0.000000 | False | 0.000000 | False | 0 | 0 | 0 | NaN | False | NaN |
| 1459904 | 7579952 | 4902 | 411 | 1041 | 70 | 31 | 52 | 23.583805 | True | 37.678195 | True | 13 | 18 | 17 | -37.678195 | True | NaN |
| -15940096 | 279952 | 0 | 0 | 0 | 0 | 0 | 0 | 0.000000 | False | 0.000000 | False | 0 | 0 | 0 | NaN | False | NaN |
| -13340096 | 8179952 | 0 | 0 | 0 | 0 | 0 | 0 | 0.000000 | False | 0.000000 | False | 0 | 0 | 0 | NaN | False | NaN |
| -11440096 | -120048 | 0 | 0 | 0 | 0 | 0 | 0 | 0.000000 | False | 0.000000 | False | 0 | 0 | 0 | NaN | False | NaN |
| -10840096 | 3379952 | 9 | 6 | 6 | 1 | 1 | 1 | 17.438833 | True | -16.293150 | False | 0 | 0 | 0 | 17.438833 | True | NaN |

## Visualization of chi values with diverging colormap¶

Configure what should be shown on hover

In [18]:

```
hover_items = { 
    '"Sunset" Chi Value':'chi_value_sunset',
    '"Sunrise" Chi Value':'chi_value_sunrise',
    '"Sunset" Chi Significant':'significant_sunset',
    '"Sunrise" Chi Significant':'significant_sunrise',
    '"Sunset" User Count':'usercount_est_sunset',
    '"Sunrise" User Count':'usercount_est_sunrise',
    '"Sunset" User Days':'userdays_est_sunset',
    '"Sunrise" User Days':'userdays_est_sunrise',
    '"Sunset" Post Count':'postcount_est_sunset',
    '"Sunrise" Post Count':'postcount_est_sunrise',
    'Expected Post Count':'postcount_est_expected',
    'Expected User Days':'userdays_est_expected',
    'Expected User Count':'usercount_est_expected',
    'Underrepresented':'underrepresented'
}
hover_items_chi = {
    f'Total {METRIC_NAME_REF[CHI_COLUMN]}':f'{CHI_COLUMN}_expected',
    'Chi-value':'chi_value',
    'Chi-significant':'significant'
}
hover_items.update(hover_items_chi)
```

Set plot options for diverging, merged chi maps

In [19]:

```
kwargs = {
    "hover_items":hover_items,
    "cmaps_diverging":("OrRd", "Blues"),
    "true_negative":False, # all + labels
    "notopicdata_color":"#F0F0F0", # grey
    "mask_nonsignificant":True # mapped to notopic class 
}
```

Plot map

In [20]:

```
gv_plot = plot_diverging(
    grid, title=(
        f'Chi value merged: '
        f'Flickr {METRIC_NAME_REF[CHI_COLUMN]} for "Sunrise" (blue) and "Sunset" (red) '
        f'(estimated, normalized to 1-1000 range) per {km_size:.0f} km grid, 2007-2018'), **kwargs)
gv_plot
```

Out[20]:

**Update interactive plotting method for diverging colormap**

In [21]:

```
def chimaps_fromcsv(
    title: Optional[str] = None,
    t_minus: str = "sunrise", t_plus: str = "sunset",
    csv_expected: Optional[str] = "flickr_all_est.csv",
    csv_observed_plus: Optional[str] = "flickr_sunset_est.csv",
    csv_observed_minus: Optional[str] = "flickr_sunrise_est.csv",
    chi_column: Optional[str] = CHI_COLUMN,
    scheme: Optional[str] = "HeadTailBreaks",
    mask_nonsignificant: bool = True,
    store_html: Optional[str] = None,
    show_cc_thumbs: Optional[bool] = False,
    plot: Optional[bool] = True,
    km_size_str: Optional[str] = km_size_str,
    normalize: Optional[bool] = True,
    hover_items: Dict[str, str] = hover_items,
    true_negative: bool = False):
    """Load, calculate and plot diverging chimaps from CSV"""
    if title is None:
        title = ""
    # load create chimap grids from CSVs 
    # with observed & expected values
    grid_plus = get_chimap_fromcsv(
        csv_expected=csv_expected,
        csv_observed=csv_observed_plus,
        chi_column=chi_column)
    grid_minus = get_chimap_fromcsv(
        csv_expected=csv_expected,
        csv_observed=csv_observed_minus,
        chi_column=chi_column)
    # normalize chi_values
    if normalize:
        for grid in [grid_minus, grid_plus]:
            norm_col_diverging(grid)
        # rare case of both max 
        # chi_values occupying the same bin:
        # use second largest chi_value for _plus
        if grid_plus["chi_value"].idxmax() == grid_minus["chi_value"].idxmax():
            mask_plus = (grid_plus["chi_value"] > 0) & \
                (grid_plus.index != grid_plus["chi_value"].idxmax())
            norm_col_diverging(grid, mask_plus=mask_plus)
    # merge grids
    grid = merge_grids(grid_plus, grid_minus, t_plus, t_minus)
    # merge chi value
    merge_chi_value(grid, chi_column=chi_column)
    # optionally attach cc thumbnails, on hover
    if t_plus == "sunset" and t_minus == "sunrise" and show_cc_thumbs:
        # attach cc thumbs from pickle
        pickles_path = OUTPUT / f"pickles{km_size_str}"
        thumb_source = {
            t_plus.title(): pickles_path / f"flickr_ccbythumbnails_{t_plus}.pkl",
            t_minus.title(): pickles_path / f"flickr_ccbythumbnails_{t_minus}.pkl"
        }
        topic = "Sunrise"
        if thumb_source.get(topic).exists():
            attach_ccthumbnail_urls(
                grid,
                pickle=thumb_source.get(topic),
                mask=grid["chi_value"] < 0)
        topic = "Sunset"
        if thumb_source.get(topic).exists():
            attach_ccthumbnail_urls(
                grid,
                pickle=thumb_source.get(topic),
                mask=grid["chi_value"] > 0)
        additional_items_thumbs = {
            'CC-Top-Image-ViewCount':'cc_img_views',
            'CC-License':'post_content_license',
            'CC-Image-URL':'cc_img_url'}
        hover_items.update(additional_items_thumbs)
    if not plot and not store_html:
        return grid
    gv_plot = plot_diverging(
        grid=grid, title=title, cmaps_diverging=("OrRd", "Blues"),
        scheme=scheme, mask_nonsignificant=mask_nonsignificant,
        store_html=store_html, hover_items=hover_items, true_negative=true_negative,
        notopicdata_color="#F0F0F0")
    if plot:
        return gv_plot
```

Execute test for userdays (instead of usercount). Notice that the balance is more towards sunset when measuring userdays instead of usercounts.

In [22]:

```
gv_plot = chimaps_fromcsv(chi_column="usercount_est", scheme="HeadTailBreaks")
gv_plot
```

Out[22]:

Preview Instagram:

- chi expected based on random 20M

In [23]:

```
plot_args = {
    # "csv_expected":"flickr_all_est.csv",
    "csv_expected":"instagram_random_est.csv",
    "csv_observed_plus":"instagram_sunset_est.csv",
    "csv_observed_minus":"instagram_sunrise_est.csv",
    "show_cc_thumbs":False, "plot":True}
```

In [24]:

```
gv_plot = chimaps_fromcsv(chi_column="usercount_est", scheme="HeadTailBreaks", **plot_args)
gv_plot
```

Out[24]:

## Process all metrics and store to HTML¶

In [25]:

```
%%time
for chi_column, name_ref in METRIC_NAME_REF.items():
    print(f"Processing {chi_column}..")
    title = (f'Chi-value merged: Flickr {name_ref} for "Sunrise" (blue) and "Sunset" (red) '
        f'(estimated, normalized to 1-1000 range) per {km_size:.0f} km grid, 2007-2018')
    filename = f"sunsetsunrise_chimap_flickr_{chi_column.removesuffix('_est')}"
    chimaps_fromcsv(
        plot=False, title=title, 
        store_html=filename,
        show_cc_thumbs=True, chi_column=chi_column)
```

```
Processing postcount_est..
Processing usercount_est..
Processing userdays_est..
CPU times: user 1min, sys: 492 ms, total: 1min
Wall time: 1min 4s
```

**Create with different classification**

In [26]:

```
%%time
for chi_column, name_ref in METRIC_NAME_REF.items():
    print(f"Processing {chi_column} (Natural Breaks)..")
    title = (f'Chi-value merged: Flickr {name_ref} for "Sunrise" (blue) and "Sunset" (red) '
        f'(estimated, normalized to 1-1000 range, Natural Breaks) per {km_size:.0f} km grid')
    filename = f"sunsetsunrise_chimap_flickr_naturalbreaks_{chi_column.removesuffix('_est')}"
    chimaps_fromcsv(
        plot=False, title=title, 
        scheme="NaturalBreaks", store_html=filename,
        show_cc_thumbs=False, chi_column=chi_column)
```

```
Processing postcount_est (Natural Breaks)..
Processing usercount_est (Natural Breaks)..
Processing userdays_est (Natural Breaks)..
CPU times: user 3min 50s, sys: 10.5 s, total: 4min
Wall time: 1min 13s
```

**Repeat for Instagram**

In [27]:

```
%%time
plot_args = {
    "csv_expected":"instagram_sunsetsunrise_est.csv",
    "csv_observed_plus":"instagram_sunset_est.csv",
    "csv_observed_minus":"instagram_sunrise_est.csv",
    "show_cc_thumbs":True, "plot":False}
for chi_column, name_ref in METRIC_NAME_REF.items():
    print(f"Processing {chi_column}..")
    title = (f'Chi-value merged: Instagram {name_ref} for "Sunrise" (blue) and "Sunset" (red) '
            f'(estimated, normalized to 1-1000 range) per {km_size:.0f} km grid')
    filename = f"sunsetsunrise_chimap_instagram_{chi_column.removesuffix('_est')}"
    chimaps_fromcsv(
        title=title,
        store_html=filename,
        chi_column=chi_column,
        **plot_args)
```

```
Processing postcount_est..
Processing usercount_est..
Processing userdays_est..
CPU times: user 1min 1s, sys: 483 ms, total: 1min 1s
Wall time: 1min 5s
```

In [28]:

```
%%time
for chi_column, name_ref in METRIC_NAME_REF.items():
    filename = f"sunsetsunrise_chimap_instagram_naturalbreaks_{chi_column.removesuffix('_est')}"
    title = (f'Chi-value merged: Instagram {name_ref} for "Sunrise" (blue) and "Sunset" (red) '
            f'(estimated, normalized to 1-1000 range, Natural Breaks) per {km_size:.0f} km grid')
    chimaps_fromcsv(
        title=title,
        store_html=filename,
        scheme="NaturalBreaks",
        chi_column=chi_column,
        **plot_args)
```

```
CPU times: user 3min 5s, sys: 6.12 s, total: 3min 11s
Wall time: 1min 19s
```

**Use Flickr expected values to calculate chi\_value for Instagram sunset+sunrise**

In [29]:

```
%%time
plot_args = {
    "csv_expected":"flickr_all_est.csv",
    "csv_observed_plus":"instagram_sunset_est.csv",
    "csv_observed_minus":"instagram_sunrise_est.csv",
    "show_cc_thumbs":True, "plot":False}
for chi_column, name_ref in METRIC_NAME_REF.items():
    title = (f'Chi-value merged, based on Flickr totals (expected values): Instagram {name_ref} for "Sunrise" (blue) and "Sunset" (red) '
            f'(estimated, normalized to 1-1000 range) per {km_size:.0f} km grid')
    filename = f"sunsetsunrise_chimap_instagram_flickrexpected_{chi_column.removesuffix('_est')}"
    chimaps_fromcsv(
        title=title,
        store_html=filename,
        chi_column=chi_column,
        **plot_args)
```

```
CPU times: user 1min, sys: 357 ms, total: 1min
Wall time: 1min 5s
```

Repeat with natural breaks:

In [30]:

```
%%time
for chi_column, name_ref in METRIC_NAME_REF.items():
    title = (f'Chi-value based on Flickr totals (expected values): Instagram {name_ref} for "Sunrise" (blue) and "Sunset" (red) '
            f'(estimated, normalized to 1-1000 range, Natural Breaks) per {km_size:.0f} km grid')
    filename = f"sunsetsunrise_chimap_instagram_flickrexpected_naturalbreaks_{chi_column.removesuffix('_est')}"
    chimaps_fromcsv(
        title=title,
        store_html=filename,
        scheme="NaturalBreaks",
        chi_column=chi_column,
        **plot_args)
```

```
CPU times: user 3min 50s, sys: 8.74 s, total: 3min 59s
Wall time: 1min 23s
```

**Use Instagram Random 20M as expected values to calculate chi\_value for Instagram sunset+sunrise**

In [31]:

```
%%time
plot_args = {
    "csv_expected":"instagram_random_est.csv",
    "csv_observed_plus":"instagram_sunset_est.csv",
    "csv_observed_minus":"instagram_sunrise_est.csv",
    "show_cc_thumbs":True, "plot":False}
for chi_column, name_ref in METRIC_NAME_REF.items():
    title = (f'Chi-value merged, expected values based on a random selection of 20M posts: Instagram {name_ref} for "Sunrise" (blue) and "Sunset" (red) '
            f'(estimated, normalized to 1-1000 range) per {km_size:.0f} km grid')
    filename = f"sunsetsunrise_chimap_instagram_randomexpected_{chi_column.removesuffix('_est')}"
    chimaps_fromcsv(
        title=title,
        store_html=filename,
        chi_column=chi_column,
        **plot_args)
```

```
CPU times: user 1min 2s, sys: 502 ms, total: 1min 3s
Wall time: 1min 7s
```

# Create notebook HTML¶

In [1]:

```
!jupyter nbconvert --to html_toc \
    --output-dir=../out/html{km_size_str} ./04_combine.ipynb \
    --template=../nbconvert.tpl \
    --ExtractOutputPreprocessor.enabled=False  >&- 2>&- # create single output file
```

Copy single HTML file to resource folder

In [2]:

```
!cp ../out/html{km_size_str}/04_combine.html ../resources/html/
```

In [ ]:

```

```
