# Supplementary material for: From sunrise to sunset: Exploring landscape preference through global reactions to ephemeral events captured in georeferenced social media
Source: PLoS One. 2023 Feb 22;18(2):e0280423. doi: 10.1371/journal.pone.0280423 (PMC9946259; doi:10.1371/journal.pone.0280423)
Supplement: S5 File — (HTML) [file pone.0280423.s005.html]

05\_countries


# Summary: Aggregation of chi values per country¶

*Alexander Dunkel, TU Dresden, Institute of Cartography; Maximilian Hartmann, Universität Zürich (UZH), Geocomputation*

---

•••

Out[1]:

Last updated: Jan-17-2023, Carto-Lab Docker Version 0.9.0

# Introduction¶

In this notebook, aggregate data per grid bin is used to generate summary data (chi square) per country. We'll use the 50 km grid data, to reduce errors from MAUP. Our goal is to see whether some countries feature a bias towards either sunset or sunrise, to support discussion of possible context factors.

# Preparations¶

## Load dependencies¶

Import code from other jupyter notebooks, synced to \*.py with jupytext:

In [2]:

```
import sys
from pathlib import Path
module_path = str(Path.cwd().parents[0] / "py")
if module_path not in sys.path:
    sys.path.append(module_path)
# import all previous chained notebooks
from _04_combine import *
```

```
Chromedriver loaded. Svg output enabled.
```

Load additional dependencies

In [3]:

```
import requests, zipfile, io
```

## Parameters¶

Activate autoreload of changed python files:

In [4]:

```
%load_ext autoreload
%autoreload 2
```

Via `gp.datasets.get_path('naturalearth_lowres')`, country geometries area easily available. However, these do not include separated spatial region subunits, which would combine all overseas regions of e.g. France together. In the admin-0 natural earth subunits dataset, these subunit areas are available.

- Natural Earth map units shapefile (1:50m)

In [5]:

```
NE_PATH = Path.cwd().parents[0] / "resources" / "naturalearth"
NE_URI = "https://www.naturalearthdata.com/http//www.naturalearthdata.com/download/50m/cultural/"
NE_FILENAME = "ne_50m_admin_0_map_subunits.zip"
```

# Country aggregation¶

## Load grid geometry¶

In [6]:

```
grid_empty = create_grid_df(
    grid_size=50000)
grid_empty = grid_to_gdf(grid_empty)
```

## Load country geometry¶

In [7]:

```
def get_zip_extract(
    uri: str, filename: str, output_path: Path, 
    create_path: bool = True, skip_exists: bool = True,
    report: bool = False):
    """Get Zip file and extract to output_path.
    Create Path if not exists."""
    if create_path:
        output_path.mkdir(
            exist_ok=True)
    if skip_exists and Path(
        output_path / filename.replace(".zip", ".shp")).exists():
        if report:
            print("File already exists.. skipping download..")
        return
    r = requests.get(f'{uri}{filename}', stream=True)
    z = zipfile.ZipFile(io.BytesIO(r.content))
    z.extractall(output_path)
```

In [8]:

```
get_zip_extract(
    uri=NE_URI,
    filename=NE_FILENAME,
    output_path=NE_PATH,
    report=True)
```

```
File already exists.. skipping download..
```

Read country shapefile to GeoDataFrame:

In [9]:

```
world = gp.read_file(
    NE_PATH / NE_FILENAME.replace(".zip", ".shp"))
```

In [10]:

```
world = world.to_crs(CRS_PROJ)
```

In [11]:

```
COLUMNS_KEEP = ['geometry','ADM0_A3','SOV_A3','ADMIN','SOVEREIGNT', 'ISO_A3', 'SU_A3']
```

In [12]:

```
def drop_cols_except(df: pd.DataFrame, columns_keep: List[str] = COLUMNS_KEEP):
    """Drop all columns from DataFrame except those specified in cols_except"""
    df.drop(
        df.columns.difference(columns_keep), axis=1, inplace=True)
```

In [13]:

```
drop_cols_except(world)
```

In [14]:

```
world.head()
```

Out[14]:

|  | SOVEREIGNT | SOV\_A3 | ADMIN | ADM0\_A3 | SU\_A3 | ISO\_A3 | geometry |
| --- | --- | --- | --- | --- | --- | --- | --- |
| 0 | Zimbabwe | ZWE | Zimbabwe | ZWE | ZWE | ZWE | POLYGON ((2987278.542 -2742733.921, 2979383.40... |
| 1 | Zambia | ZMB | Zambia | ZMB | ZMB | ZMB | POLYGON ((2976200.722 -1924957.705, 2961959.54... |
| 2 | Yemen | YEM | Yemen | YEM | YEM | YEM | MULTIPOLYGON (((5181525.454 2047361.573, 51352... |
| 3 | Yemen | YEM | Yemen | YEM | YES | -99 | POLYGON ((5307347.563 1557616.990, 5313584.814... |
| 4 | Vietnam | VNM | Vietnam | VNM | VNM | VNM | MULTIPOLYGON (((10323687.558 1282070.654, 1032... |

In [15]:

```
world[world["SOVEREIGNT"] == "France"].head(10)
```

Out[15]:

|  | SOVEREIGNT | SOV\_A3 | ADMIN | ADM0\_A3 | SU\_A3 | ISO\_A3 | geometry |
| --- | --- | --- | --- | --- | --- | --- | --- |
| 204 | France | FR1 | France | FRA | FXC | -99 | POLYGON ((783658.015 5100500.165, 782655.467 5... |
| 205 | France | FR1 | France | FRA | FXX | FRA | MULTIPOLYGON (((597064.157 5619098.479, 587921... |
| 206 | France | FR1 | France | FRA | MYT | MYT | POLYGON ((4456352.999 -1599245.685, 4450078.32... |
| 207 | France | FR1 | France | FRA | REU | REU | POLYGON ((5351998.016 -2615032.781, 5337783.57... |
| 208 | France | FR1 | France | FRA | MTQ | MTQ | POLYGON ((-5975639.717 1784830.421, -5977605.9... |
| 209 | France | FR1 | France | FRA | GLP | GLP | MULTIPOLYGON (((-5993924.853 1996500.313, -600... |
| 210 | France | FR1 | France | FRA | GUF | GUF | POLYGON ((-5471007.672 287661.361, -5469831.69... |
| 211 | France | FR1 | Saint Pierre and Miquelon | SPM | SPM | SPM | MULTIPOLYGON (((-4445751.267 5530331.017, -444... |
| 212 | France | FR1 | Wallis and Futuna | WLF | WLF | WLF | MULTIPOLYGON (((-17360004.526 -1642824.256, -1... |
| 213 | France | FR1 | Saint Martin | MAF | MAF | MAF | POLYGON ((-6120916.004 2219901.617, -6131782.6... |

In [16]:

```
world.plot()
```

Out[16]:

```
<AxesSubplot:>
```

Define column to use for country-aggregation:

In [17]:

```
COUNTRY_COL = 'SU_A3'
```

These SU\_A3 country codes are extended ISO codes, see this ref table.

Only keep COUNTRY\_COL column, SOVEREIGNT, and geometry:

In [18]:

```
columns_keep = ['geometry', 'SOVEREIGNT', COUNTRY_COL]
drop_cols_except(world, columns_keep)
```

## Country overlay with grid¶

First, write multi-index to columns, to later re-create the index:

In [19]:

```
grid_empty['xbin'] = grid_empty.index.get_level_values(0)
grid_empty['ybin'] = grid_empty.index.get_level_values(1)
```

Create an overlay, only stroing country-grid intersection:

In [20]:

```
%%time
grid_overlay = gp.overlay(
    grid_empty, world, 
    how='intersection')
```

```
CPU times: user 1min 10s, sys: 196 ms, total: 1min 10s
Wall time: 1min 10s
```

In [21]:

```
grid_overlay[grid_overlay[COUNTRY_COL] == "DEU"].head()
```

Out[21]:

|  | xbin | ybin | SOVEREIGNT | SU\_A3 | geometry |
| --- | --- | --- | --- | --- | --- |
| 37091 | 409904 | 6079952 | Germany | DEU | POLYGON ((459904.000 6031192.645, 457473.846 6... |
| 37092 | 409904 | 6029952 | Germany | DEU | POLYGON ((459904.000 5979952.000, 439777.346 5... |
| 37093 | 409904 | 5979952 | Germany | DEU | POLYGON ((459904.000 5979952.000, 459904.000 5... |
| 37094 | 459904 | 6179952 | Germany | DEU | POLYGON ((509904.000 6129952.000, 495314.233 6... |
| 37095 | 459904 | 6129952 | Germany | DEU | MULTIPOLYGON (((509904.000 6079952.000, 502808... |

In [22]:

```
grid_overlay[
    grid_overlay[COUNTRY_COL].isin(["DEU", "FXX"])].plot(
    edgecolor='white', column=COUNTRY_COL, linewidth=0.3)
```

Out[22]:

```
<AxesSubplot:>
```

Calculate area:

In [23]:

```
grid_overlay["area"] = grid_overlay.area
```

In [24]:

```
grid_overlay[grid_overlay[COUNTRY_COL] == "DEU"].head()
```

Out[24]:

|  | xbin | ybin | SOVEREIGNT | SU\_A3 | geometry | area |
| --- | --- | --- | --- | --- | --- | --- |
| 37091 | 409904 | 6079952 | Germany | DEU | POLYGON ((459904.000 6031192.645, 457473.846 6... | 3.886671e+08 |
| 37092 | 409904 | 6029952 | Germany | DEU | POLYGON ((459904.000 5979952.000, 439777.346 5... | 1.891485e+08 |
| 37093 | 409904 | 5979952 | Germany | DEU | POLYGON ((459904.000 5979952.000, 459904.000 5... | 2.931637e+08 |
| 37094 | 459904 | 6179952 | Germany | DEU | POLYGON ((509904.000 6129952.000, 495314.233 6... | 2.813983e+08 |
| 37095 | 459904 | 6129952 | Germany | DEU | MULTIPOLYGON (((509904.000 6079952.000, 502808... | 2.012630e+08 |

In [25]:

```
grid_overlay.groupby(["xbin", "ybin"], sort=False).head()
```

Out[25]:

|  | xbin | ybin | SOVEREIGNT | SU\_A3 | geometry | area |
| --- | --- | --- | --- | --- | --- | --- |
| 0 | -17640096 | -1970048 | Fiji | FJI | POLYGON ((-17596068.762 -1988932.933, -1759674... | 1.375008e+07 |
| 1 | -17590096 | -2020048 | Fiji | FJI | MULTIPOLYGON (((-17558604.458 -2070048.000, -1... | 2.640252e+08 |
| 2 | -17590096 | -2070048 | Fiji | FJI | POLYGON ((-17544904.267 -2070048.000, -1754482... | 1.000075e+08 |
| 3 | -17490096 | -2070048 | Fiji | FJI | POLYGON ((-17440096.000 -2113755.099, -1744249... | 1.220128e+07 |
| 4 | -17440096 | -2070048 | Fiji | FJI | POLYGON ((-17435352.110 -2120048.000, -1743544... | 4.561355e+07 |
| ... | ... | ... | ... | ... | ... | ... |
| 70868 | 17009904 | 929952 | Marshall Islands | MHL | POLYGON ((17059904.000 883328.346, 17059165.99... | 7.021293e+05 |
| 70869 | 17059904 | 929952 | Marshall Islands | MHL | POLYGON ((17064020.522 879952.000, 17059904.00... | 2.124275e+07 |
| 70870 | 17059904 | 879952 | Marshall Islands | MHL | POLYGON ((17070021.406 879952.000, 17079826.49... | 7.197785e+07 |
| 70871 | 17109904 | 879952 | Marshall Islands | MHL | POLYGON ((17119886.235 867969.633, 17127193.92... | 3.998398e+07 |
| 70872 | 16709904 | -20048 | Nauru | NRU | POLYGON ((16730605.928 -68100.872, 16728352.51... | 2.785668e+07 |

70873 rows × 6 columns

Next steps:

- group by xbin/ybin and select max area per group
- get max id from area comparison per group
- select ISO\_A3 column to assign values back (based on max area per bin)

In [26]:

```
idx_maxarea = grid_overlay.groupby(
    ["xbin", "ybin"], sort=False)['area'].idxmax()
```

In [27]:

```
idx_maxarea.head()
```

Out[27]:

```
xbin       ybin    
-17640096  -1970048    0
-17590096  -2020048    1
           -2070048    2
-17490096  -2070048    3
-17440096  -2070048    4
Name: area, dtype: int64
```

In [28]:

```
bin_adm_maxarea = grid_overlay.loc[
    idx_maxarea, ["xbin", "ybin", COUNTRY_COL]]
```

Recreate index (Note: duplicate xbin/ybin indexes exist):

In [29]:

```
bin_adm_maxarea.set_index(
    ['xbin', 'ybin'], inplace=True)
```

In [30]:

```
bin_adm_maxarea.head()
```

Out[30]:

|  |  | SU\_A3 |
| --- | --- | --- |
| xbin | ybin |  |
| -17640096 | -1970048 | FJI |
| -17590096 | -2020048 | FJI |
|  | -2070048 | FJI |
| -17490096 | -2070048 | FJI |
| -17440096 | -2070048 | FJI |

Assign back to grid:

In [31]:

```
grid_empty.loc[
    bin_adm_maxarea.index,
    COUNTRY_COL] = bin_adm_maxarea[COUNTRY_COL]
```

Set nan to Empty class

In [32]:

```
grid_empty.loc[
    grid_empty[COUNTRY_COL].isna(),
    COUNTRY_COL] = "Empty"
```

In [33]:

```
grid_empty[grid_empty[COUNTRY_COL] != "Empty"].head()
```

Out[33]:

|  |  | geometry | xbin | ybin | SU\_A3 |
| --- | --- | --- | --- | --- | --- |
| xbin | ybin |  |  |  |  |
| -17640096 | -1970048 | POLYGON ((-17640096.000 -1970048.000, -1759009... | -17640096 | -1970048 | FJI |
| -17590096 | -2020048 | POLYGON ((-17590096.000 -2020048.000, -1754009... | -17590096 | -2020048 | FJI |
|  | -2070048 | POLYGON ((-17590096.000 -2070048.000, -1754009... | -17590096 | -2070048 | FJI |
| -17540096 | -1720048 | POLYGON ((-17540096.000 -1720048.000, -1749009... | -17540096 | -1720048 | WLF |
| -17490096 | -570048 | POLYGON ((-17490096.000 -570048.000, -17440096... | -17490096 | -570048 | KIR |

Check assignment

In [34]:

```
fig, ax = plt.subplots(1, 1, figsize=(10,12))
bbox_italy = (
    7.8662109375, 36.24427318493909,
    19.31396484375, 43.29320031385282)
buf = 1000000
# create bounds from WGS1984 italy and project to Mollweide
minx, miny = PROJ_TRANSFORMER.transform(
    bbox_italy[0], bbox_italy[1])
maxx, maxy = PROJ_TRANSFORMER.transform(
    bbox_italy[2], bbox_italy[3])
ax.set_xlim(minx-buf, maxx+buf)
ax.set_ylim(miny-buf, maxy+buf)
empty = grid_empty[grid_empty[COUNTRY_COL] == "Empty"].plot(
    ax=ax, edgecolor=None, facecolor='white', linewidth=0)
base = grid_empty[grid_empty[COUNTRY_COL] != "Empty"].plot(
    ax=ax, edgecolor='white', column=COUNTRY_COL, linewidth=0.3)
world.plot(
    ax=base, color='none', edgecolor='black', linewidth=0.2)
```

Out[34]:

```
<AxesSubplot:>
```

In [35]:

```
grid_empty.head()
```

Out[35]:

|  |  | geometry | xbin | ybin | SU\_A3 |
| --- | --- | --- | --- | --- | --- |
| xbin | ybin |  |  |  |  |
| -18040096 | 8979952 | POLYGON ((-18040096.000 8979952.000, -17990096... | -18040096 | 8979952 | Empty |
|  | 8929952 | POLYGON ((-18040096.000 8929952.000, -17990096... | -18040096 | 8929952 | Empty |
|  | 8879952 | POLYGON ((-18040096.000 8879952.000, -17990096... | -18040096 | 8879952 | Empty |
|  | 8829952 | POLYGON ((-18040096.000 8829952.000, -17990096... | -18040096 | 8829952 | Empty |
|  | 8779952 | POLYGON ((-18040096.000 8779952.000, -17990096... | -18040096 | 8779952 | Empty |

**Combine in a single method:**

In [36]:

```
def grid_assign_country(
    grid: gp.GeoDataFrame, countries: gp.GeoDataFrame,
    country_col: Optional[str] = COUNTRY_COL):
    """Assign countries code based on max area overlay to grid"""
    # get index as column
    grid['xbin'] = grid.index.get_level_values(0)
    grid['ybin'] = grid.index.get_level_values(1)
    # intersect
    grid_overlay = gp.overlay(
        grid, countries, 
        how='intersection')
    # calculate area
    grid_overlay["area"] = grid_overlay.area
    # select indexes based on area overlay
    idx_maxarea = grid_overlay.groupby(
        ["xbin", "ybin"], sort=False)["area"].idxmax()
    bin_country_maxarea = grid_overlay.loc[
        idx_maxarea, ["xbin", "ybin", country_col]]
    # recreate grid index
    bin_country_maxarea.set_index(
        ['xbin', 'ybin'], inplace=True)
    # assign country back to grid
    grid.loc[
        bin_country_maxarea.index,
        country_col] = bin_country_maxarea[country_col]
    # drop index columns not needed anymore
    grid.drop(
        ["xbin", "ybin"], axis=1, inplace=True)
```

### Optional: TFIDF country data¶

For exploring TFIDF per country, we'll also export country data here for PostGIS

**Grid to Postgis**

For further work, we'll export the SQL syntax here to import the 100km Grid to to Postgis (optional).
In addition,

- we'll need to insert the Mollweide projection string to the spatial ref table.
- and create a table for the grid:

```
CREATE TABLE spatial. "grid100km" (
    xbin int,
    ybin int,
    PRIMARY KEY (xbin, ybin),
    su_a3 char(3),
    geometry geometry(Polygon, 54009)
);
```

In [37]:

```
grid_empty[grid_empty[COUNTRY_COL] == "USB"].plot()
```

Out[37]:

```
<AxesSubplot:>
```

In [38]:

```
from shapely.wkt import dumps as wkt_dumps
    
def add_wkt_col(gdf: gp.GeoDataFrame):
    """Converts gdf.geometry to WKT (Well-Known-Text) as new column
    Requires `from shapely.wkt import dumps as wkt_dumps`"""
    gdf.loc[gdf.index, "geom_wkt"] = gdf["geometry"].apply(lambda x: wkt_dumps(x))
```

In [39]:

```
add_wkt_col(grid_empty)

value_list = ',\n'.join(
    f"({index[0]},{index[1]}, '{row[COUNTRY_COL]}', 'SRID=54009;{row.geom_wkt}')"
    for index, row in grid_empty[grid_empty[COUNTRY_COL] != "Empty"].iterrows())
with open(OUTPUT / "csv" / "grid_100km_Mollweide_WKT.sql", 'w') as wkt_file:
    wkt_file.write(
        f'''
        INSERT INTO spatial."grid100km" (
            xbin,ybin,su_a3,geometry)
        VALUES
        {value_list};
        ''')
```

**Country (su\_a3) to Postgis**

```
CREATE TABLE spatial. "country_sua3" (
    su_a3 char(3),
    PRIMARY KEY (su_a3),
    geometry geometry(Geometry, 54009)
);
```

In [40]:

```
world_empty = world.copy().set_index("SU_A3").drop(columns=['SOVEREIGNT'])
add_wkt_col(world_empty)
```

In [41]:

```
world_empty.head()
```

Out[41]:

|  | geometry | geom\_wkt |
| --- | --- | --- |
| SU\_A3 |  |  |
| ZWE | POLYGON ((2987278.542 -2742733.921, 2979383.40... | POLYGON ((2987278.5419494602829218 -2742733.92... |
| ZMB | POLYGON ((2976200.722 -1924957.705, 2961959.54... | POLYGON ((2976200.7224715156480670 -1924957.70... |
| YEM | MULTIPOLYGON (((5181525.454 2047361.573, 51352... | MULTIPOLYGON (((5181525.4542204160243273 20473... |
| YES | POLYGON ((5307347.563 1557616.990, 5313584.814... | POLYGON ((5307347.5630814759060740 1557616.990... |
| VNM | MULTIPOLYGON (((10323687.558 1282070.654, 1032... | MULTIPOLYGON (((10323687.5583364013582468 1282... |

In [42]:

```
value_list = ',\n'.join(
    f"('{index}', 'SRID=54009;{row.geom_wkt}')"
    for index, row in world_empty.iterrows())
with open(OUTPUT / "csv" / "country_sua3_Mollweide_WKT.sql", 'w') as wkt_file:
    wkt_file.write(
        f'''
        INSERT INTO spatial. "country_sua3" (
            su_a3,geometry)
        VALUES
        {value_list};
        ''')
```

## Load benchmark data¶

In [43]:

```
grid = pd.read_pickle(
    OUTPUT / f"pickles_50km" / "flickr_userdays_all_est_hll.pkl")
```

In [44]:

```
grid["userdays_hll"].dropna().head()
```

Out[44]:

```
xbin       ybin   
-18040096   29952     \x138b4006221e8426e32a853ee4512366876f017dc384...
           -20048                                          \x138b4038c3
           -70048                                          \x138b402f41
-17840096   129952                                         \x138b401ae1
           -20048                                          \x138b403fe2
Name: userdays_hll, dtype: object
```

In [45]:

```
grid.dropna().head()
```

Out[45]:

|  |  | userdays\_hll | geometry |
| --- | --- | --- | --- |
| xbin | ybin |  |  |
| -18040096 | 29952 | \x138b4006221e8426e32a853ee4512366876f017dc384... | POLYGON ((-18040096.000 29952.000, -17990096.0... |
|  | -20048 | \x138b4038c3 | POLYGON ((-18040096.000 -20048.000, -17990096.... |
|  | -70048 | \x138b402f41 | POLYGON ((-18040096.000 -70048.000, -17990096.... |
| -17840096 | 129952 | \x138b401ae1 | POLYGON ((-17840096.000 129952.000, -17790096.... |
|  | -20048 | \x138b403fe2 | POLYGON ((-17840096.000 -20048.000, -17790096.... |

## Assign Countries to grid:¶

The actual assignment takes pretty long. We will later store the assignment (bin-idx, country-idx) in a pickle that can be loaded.

In [46]:

```
%%time
grid_assign_country(
    grid, world, country_col=COUNTRY_COL)
```

```
CPU times: user 1min 12s, sys: 95.7 ms, total: 1min 12s
Wall time: 1min 12s
```

In [47]:

```
grid.head()
```

Out[47]:

|  |  | userdays\_hll | geometry | SU\_A3 |
| --- | --- | --- | --- | --- |
| xbin | ybin |  |  |  |
| -18040096 | 8979952 | NaN | POLYGON ((-18040096.000 8979952.000, -17990096... | NaN |
|  | 8929952 | NaN | POLYGON ((-18040096.000 8929952.000, -17990096... | NaN |
|  | 8879952 | NaN | POLYGON ((-18040096.000 8879952.000, -17990096... | NaN |
|  | 8829952 | NaN | POLYGON ((-18040096.000 8829952.000, -17990096... | NaN |
|  | 8779952 | NaN | POLYGON ((-18040096.000 8779952.000, -17990096... | NaN |

In [48]:

```
grid.plot(edgecolor='white', column=COUNTRY_COL, figsize=(22,28), linewidth=0.3)
```

Out[48]:

```
<AxesSubplot:>
```

## Merge hll sets per country and estimate cardinality¶

Merge hll sets per country id, connect to hll worker db:

In [49]:

```
DB_CONN = psycopg2.connect(
        host=DB_HOST,
        port=DB_PORT ,
        dbname=DB_NAME,
        user=DB_USER,
        password=DB_PASS
)
DB_CONN.set_session(
    readonly=True)
DB_CALC = tools.DbConn(
    DB_CONN)
CUR_HLL = DB_CONN.cursor()
```

In [50]:

```
db_conn = tools.DbConn(DB_CONN)
db_conn.query("SELECT 1;")
```

Out[50]:

|  | ?column? |
| --- | --- |
| 0 | 1 |

## HLL Union¶

In [51]:

```
def union_hll(
    hll_series: pd.Series, db_conn: tools.DbConn, cardinality: bool = True,
    group_by: Optional[pd.Series] = None) -> pd.Series:
    """HLL Union and (optional) cardinality estimation from series of hll sets
    based on group by composite index.

    Args:
        hll_series: Indexed series (bins) of hll sets. 
        cardinality: If True, returns cardinality (counts). Otherwise,
            the unioned hll set will be returned.
        group_by: Optional Provide Series to group hll sets by. If None,
            Index will be used.
            
    The method will combine all groups of hll sets first,
        in a single SQL command. Union of hll hll-sets belonging 
        to the same group (bin) and (optionally) returning the cardinality 
        (the estimated count) per group will be done in postgres.
    
    By utilizing Postgres´ GROUP BY (instead of, e.g. doing 
        the group with numpy), it is possible to reduce the number
        of SQL calls to a single run, which saves overhead 
        (establishing the db connection, initializing the SQL query 
        etc.). Also note that ascending integers are used for groups,
        instead of their full original bin-ids, which also reduces
        transfer time.
    
    cardinality = True should be used when calculating counts in
        a single pass.
        
    cardinality = False should be used when incrementally union
        of hll sets is required, e.g. due to size of input data.
        In the last run, set to cardinality = True.
    """
    if group_by is None:
        group_by_series = hll_series.index
    else:
        group_by_series = group_by
    # group all hll-sets per index (bin-id)
    series_grouped = hll_series.groupby(
        group_by_series).apply(list)
    # From grouped hll-sets,
    # construct a single SQL Value list;
    # if the following nested list comprehension
    # doesn't make sense to you, have a look at
    # spapas.github.io/2016/04/27/python-nested-list-comprehensions/
    # with a decription on how to 'unnest'
    # nested list comprehensions to regular for-loops
    hll_values_list = ",".join(
        [f"({ix}::int,'{hll_item}'::hll)" 
         for ix, hll_items
         in enumerate(series_grouped.values.tolist())
         for hll_item in hll_items])
    # Compilation of SQL query,
    # depending on whether to return the cardinality
    # of unioned hll or the unioned hll
    return_col = "hll_union"
    hll_calc_pre = ""
    hll_calc_tail = "AS hll_union"
    if cardinality:
        # add sql syntax for cardinality 
        # estimation
        # (get count distinct from hll)
        return_col = "hll_cardinality"
        hll_calc_pre = "hll_cardinality("
        hll_calc_tail = ")::int"
    db_query = f"""
        SELECT sq.{return_col} FROM (
            SELECT s.group_ix,
                   {hll_calc_pre}
                   hll_union_agg(s.hll_set)
                   {hll_calc_tail}
            FROM (
                VALUES {hll_values_list}
                ) s(group_ix, hll_set)
            GROUP BY group_ix
            ORDER BY group_ix ASC) sq
        """
    df = db_conn.query(db_query)
    # to merge values back to grouped dataframe,
    # first reset index to ascending integers
    # matching those of the returned df;
    # this will turn series_grouped into a DataFrame;
    # the previous index will still exist in column 'index'
    df_grouped = series_grouped.reset_index()
    # drop hll sets not needed anymore
    df_grouped.drop(columns=[hll_series.name], inplace=True)
    # append hll_cardinality counts 
    # using matching ascending integer indexes
    df_grouped.loc[df.index, return_col] = df[return_col]
    # set index back to original bin-ids
    df_grouped.set_index(group_by_series.name, inplace=True)
    # return column as indexed pd.Series
    return df_grouped[return_col]
```

In [52]:

```
%%time
cardinality_series = union_hll(
    hll_series=grid["userdays_hll"].dropna(),
    group_by=grid[COUNTRY_COL],
    db_conn=db_conn)
```

```
CPU times: user 225 ms, sys: 64.8 ms, total: 290 ms
Wall time: 1.08 s
```

In [53]:

```
cardinality_series.head()
```

Out[53]:

```
SU_A3
ABW    6548
ACA    4837
ACB     207
AFG    7472
AGO    4243
Name: hll_cardinality, dtype: int64
```

## Assign HLL Cardinality to Countries¶

In [54]:

```
world.set_index(COUNTRY_COL, inplace=True)
```

In [55]:

```
world.loc[
    cardinality_series.index,
    "userdays_est"] = cardinality_series
```

Calculate area and normalize:

In [56]:

```
world["area"] = world.area
world["userdays_est_norm"] = ((world.userdays_est ** 2) / world.area)
```

In [57]:

```
world.head()
```

Out[57]:

|  | SOVEREIGNT | geometry | userdays\_est | area | userdays\_est\_norm |
| --- | --- | --- | --- | --- | --- |
| SU\_A3 |  |  |  |  |  |
| ZWE | Zimbabwe | POLYGON ((2987278.542 -2742733.921, 2979383.40... | 4969.0 | 3.910570e+11 | 0.000063 |
| ZMB | Zambia | POLYGON ((2976200.722 -1924957.705, 2961959.54... | 8066.0 | 7.572867e+11 | 0.000086 |
| YEM | Yemen | MULTIPOLYGON (((5181525.454 2047361.573, 51352... | 2950.0 | 4.516298e+11 | 0.000019 |
| YES | Yemen | POLYGON ((5307347.563 1557616.990, 5313584.814... | 346.0 | 3.526072e+09 | 0.000034 |
| VNM | Vietnam | MULTIPOLYGON (((10323687.558 1282070.654, 1032... | 214834.0 | 3.296746e+11 | 0.139998 |

Preview plot:

In [58]:

```
fig, ax = plt.subplots(1, 1, figsize=(22,28))
world.plot(
    column='userdays_est_norm',
    cmap='OrRd',
    ax=ax,
    linewidth=0.2,
    edgecolor='grey',
    legend=True,
    scheme='headtail_breaks')
```

Out[58]:

```
<AxesSubplot:>
```

Prepare method:

In [59]:

```
def merge_countries_grid(
    grid_countries: gp.GeoDataFrame, grid: gp.GeoDataFrame, 
    usecols: List[str], mask: Optional[pd.Series] = None):
    """Merge temporary country assigned grid data to grid geodataframe
    
    Args:
        grid_countries: Indexed GeoDataFrame with country data
        grid: Indexed GeoDataFrame target
        usecols: Col names to merge from countries
        mask: Optional boolean mask (pd.Series)
              to partially merge country data
    """
    for col in usecols:
        if mask is None:
            grid.loc[grid_countries.index, col] = grid_countries[col]
            continue
        grid_countries_mask = grid_countries.loc[mask, col]
        grid.loc[grid_countries_mask.index, col] = grid_countries_mask
        
def group_union_cardinality(
    countries: gp.GeoDataFrame, grid: gp.GeoDataFrame, 
    db_conn: tools.DbConn, 
    metric_hll: str = "usercount_hll",
    country_col: str = COUNTRY_COL,
    grid_countries_pickle: Optional[Path] = None):
    """Group hll sets per country and assign cardinality to countries
    
    Args:
        grid: Indexed GeoDataFrame with hll data to use in union
        countries: Country GeoDataFrame to group grid-bins and assign
            cardinality.
        country_col: Name/ID of column in country to use in group by
        metric_hll: the name of HLL column that is used in hll union
        db_conn: A (read-only) DB connection to PG HLL Worker, for HLL
            calculation.
        grid_countries_pickle: Optional path to store and load intermediate
            grid-country assignment
    """
    if grid_countries_pickle and grid_countries_pickle.exists():
        grid_countries_tmp = pd.read_pickle(
            grid_countries_pickle).to_frame()
        merge_countries_grid(
            grid_countries=grid_countries_tmp,
            grid=grid,
            usecols=[country_col])
    else:
        grid_assign_country(
            grid, countries, country_col=country_col)
        # store intermediate, to speed up later runs    
        if grid_countries_pickle:
            grid[country_col].to_pickle(
                grid_countries_pickle)
            print("Intermediate country-grid assignment written..")
    # calculate cardinality by hll union
    cardinality_series = union_hll(
        hll_series=grid[metric_hll].dropna(),
        group_by=grid[country_col],
        db_conn=db_conn)
    # set index
    if not countries.index.name == country_col:
        countries.set_index(country_col, inplace=True)
    # assign cardinality
    countries.loc[
        cardinality_series.index,
        metric_hll.replace("_hll", "_est")] = cardinality_series
```

In [60]:

```
def country_agg_frompickle(
    grid_pickle: Path, db_conn: tools.DbConn,
    ne_path: Path = NE_PATH, ne_filename: str = NE_FILENAME, 
    ne_uri: str = NE_URI, country_col: str = COUNTRY_COL,
    metric_hll: str = "usercount_hll") -> gp.GeoDataFrame:
    """Load grid pickle, load country shapefile, join cardinality to country
    and return country GeoDataFrame"""
    # prepare country gdf
    get_zip_extract(
        uri=ne_uri,
        filename=ne_filename,
        output_path=ne_path)
    world = gp.read_file(
        ne_path / ne_filename.replace(".zip", ".shp"))
    world = world.to_crs(CRS_PROJ)
    columns_keep = ['geometry', country_col]
    drop_cols_except(world, columns_keep)
    # prepare grid gdf
    grid = pd.read_pickle(
        grid_pickle)
    # check if intermediate country agg file already exists
    grid_countries_pickle = Path(
        ne_path / f'{km_size_str}_{ne_filename.replace(".zip", ".pickle")}')
    group_union_cardinality(
        world, grid, country_col=country_col, db_conn=db_conn,
        grid_countries_pickle=grid_countries_pickle, metric_hll=metric_hll)
    return world
```

Test:

- for userdays
- for usercount

In [61]:

```
metrics = ["userdays", "usercount"]
```

In [62]:

```
%%time
for metric in metrics:
    world = country_agg_frompickle(
        grid_pickle=OUTPUT / f"pickles_50km" / f"flickr_{metric}_all_est_hll.pkl",
        db_conn=db_conn, metric_hll=f"{metric}_hll")
    fig, ax = plt.subplots(1, 1, figsize=(22,28))
    ax.set_title(metric.capitalize())
    world.plot(
        column=f'{metric}_est',
        cmap='OrRd',
        ax=ax,
        linewidth=0.2,
        edgecolor='grey',
        legend=True,
        scheme='headtail_breaks')
```

```
CPU times: user 7.87 s, sys: 395 ms, total: 8.26 s
Wall time: 9.63 s
```

# Calculate Chi¶

For chi, we need to combine results from expected versus observed per country. Basically, repeat the grid chi aggregation, just for countries.

In [63]:

```
CHI_COLUMN = f"usercount_est"
METRIC = CHI_COLUMN.replace("_est", "")
```

Calculate chi value according to 03\_combine.ipynb

In [64]:

```
%%time
world_observed = country_agg_frompickle(
    grid_pickle=OUTPUT / f"pickles_50km" / f"flickr_{METRIC}_sunset_est_hll.pkl",
    db_conn=db_conn, metric_hll=CHI_COLUMN.replace("_est", "_hll"))
world_expected = country_agg_frompickle(
    grid_pickle=OUTPUT / f"pickles_50km" / f"flickr_{METRIC}_all_est_hll.pkl",
    db_conn=db_conn, metric_hll=CHI_COLUMN.replace("_est", "_hll"))
```

```
CPU times: user 7.17 s, sys: 165 ms, total: 7.33 s
Wall time: 8.13 s
```

Calculate chi:

In [65]:

```
def calculate_country_chi(
        gdf_expected: gp.GeoDataFrame, gdf_observed: gp.GeoDataFrame,
        chi_column: str = CHI_COLUMN) -> gp.GeoDataFrame:
    """Calculate chi for expected vs observed based on two geodataframes (country geom)"""
    norm_val = calc_norm(
        gdf_expected, gdf_observed, chi_column=chi_column)
    rename_expected = {
        chi_column:f'{chi_column}_expected',
        }
    gdf_expected.rename(
        columns=rename_expected,
        inplace=True)
    merge_cols = [chi_column]
    gdf_expected_observed = gdf_expected.merge(
        gdf_observed[merge_cols],
        left_index=True, right_index=True)
    apply_chi_calc(
        grid=gdf_expected_observed,
        norm_val=norm_val,
        chi_column=chi_column)
    return gdf_expected_observed
```

In [66]:

```
%%time
world_expected_observed = calculate_country_chi(world_expected, world_observed)
```

```
CPU times: user 11.2 ms, sys: 60 µs, total: 11.2 ms
Wall time: 11 ms
```

In [67]:

```
world_expected_observed.head()
```

Out[67]:

|  | geometry | usercount\_est\_expected | usercount\_est | chi\_value | significant |
| --- | --- | --- | --- | --- | --- |
| SU\_A3 |  |  |  |  |  |
| ZWE | POLYGON ((2987278.542 -2742733.921, 2979383.40... | 1345.0 | 158.0 | 10.816126 | True |
| ZMB | POLYGON ((2976200.722 -1924957.705, 2961959.54... | 2511.0 | 260.0 | 7.085320 | True |
| YEM | MULTIPOLYGON (((5181525.454 2047361.573, 51352... | 740.0 | 38.0 | -11.804480 | True |
| YES | POLYGON ((5307347.563 1557616.990, 5313584.814... | 93.0 | 11.0 | 2.929983 | False |
| VNM | MULTIPOLYGON (((10323687.558 1282070.654, 1032... | 31437.0 | 1442.0 | -87.653996 | True |

In [68]:

```
fig, ax = plt.subplots(1, 1, figsize=(22,28))
world_expected_observed.plot(
    column='chi_value',
    cmap='OrRd',
    ax=ax,
    linewidth=0.2,
    edgecolor='grey',
    legend=True,
    scheme='headtail_breaks')
```

Out[68]:

```
<AxesSubplot:>
```

Store full country chi gdf as CSV (used in 06\_relationships.ipynb):

In [69]:

```
cols: List[str] = [f"{METRIC}_est_expected", f"{METRIC}_est", "chi_value", "significant"]
world_expected_observed.to_csv(OUTPUT / "csv" / f"countries_{METRIC}_chi_flickr_sunset.csv", mode='w', columns=cols, index=True)
```

Combine everything in one function:

In [70]:

```
def load_store_country_chi(
    topic: str, source: str, metric: str = METRIC, flickrexpected: bool = False, return_gdf: bool = None,
    store_csv: bool = False):
    """Load, calculate and plot country chi map based on topic (sunset, sunrise)
    and source (instagram, flickr)
    """
    chi_column = f"{metric}_est"
    world_observed = country_agg_frompickle(
        grid_pickle=OUTPUT / f"pickles_50km" / f"{source}_{metric}_{topic}_est_hll.pkl",
        db_conn=db_conn,
        metric_hll=f"{metric}_hll")
    if not flickrexpected and source == "instagram":
        # expected all not available for Instagram
        expected = f"{source}_{metric}_random"
    else:
        expected = f'flickr_{metric}_all'
    world_expected = country_agg_frompickle(
        grid_pickle=OUTPUT / f"pickles_50km" / f"{expected}_est_hll.pkl",
        db_conn=db_conn,
        metric_hll=f"{metric}_hll")
    # calculate chi
    world_expected_observed = calculate_country_chi(
        world_expected, world_observed, chi_column=chi_column)
    # store intermediate csv
    cols: List[str] = [
        f"{metric}_est_expected", f"{metric}_est", "chi_value", "significant"]
    ext = ""
    if flickrexpected and source == "instagram":
        ext = "_fe"
    if store_csv:
        world_expected_observed.to_csv(
            OUTPUT / "csv" / f"countries_{metric}_chi_{source}_{topic}{ext}.csv",
            mode='w', columns=cols, index=True)
    if return_gdf:
        return world_expected_observed
```

Repeat for userdays:

In [71]:

```
%%time
load_store_country_chi(
    topic="sunset", source="flickr", metric="userdays", store_csv = True)
```

```
CPU times: user 6.91 s, sys: 172 ms, total: 7.08 s
Wall time: 7.88 s
```

**Repeat for postcount:**

In [72]:

```
%%time
load_store_country_chi(
    topic="sunrise", source="flickr", metric="postcount", store_csv = True)
```

```
CPU times: user 6.9 s, sys: 242 ms, total: 7.14 s
Wall time: 8.2 s
```

In [73]:

```
%%time
load_store_country_chi(
    topic="sunset", source="flickr", metric="postcount", store_csv = True)
```

```
CPU times: user 6.16 s, sys: 245 ms, total: 6.4 s
Wall time: 7.54 s
```

In [74]:

```
%%time
load_store_country_chi(
    topic="sunrise", source="instagram", metric="postcount", store_csv = True)
```

```
CPU times: user 6.82 s, sys: 169 ms, total: 6.99 s
Wall time: 7.48 s
```

In [75]:

```
%%time
load_store_country_chi(
    topic="sunset", source="instagram", metric="postcount", store_csv = True)
```

```
CPU times: user 5.8 s, sys: 172 ms, total: 5.97 s
Wall time: 6.48 s
```

**Repeat for Sunrise**

In [76]:

```
%%time
load_store_country_chi(
    topic="sunrise", source="flickr", metric="usercount", store_csv = True)
```

```
CPU times: user 6.54 s, sys: 120 ms, total: 6.65 s
Wall time: 7.24 s
```

In [77]:

```
load_store_country_chi(
    topic="sunrise", source="flickr", metric="userdays", store_csv = True)
```

**Repeat for Instagram**

In [78]:

```
%%time
load_store_country_chi(
    topic="sunrise", source="instagram", metric="usercount", store_csv = True)
```

```
CPU times: user 5.8 s, sys: 140 ms, total: 5.94 s
Wall time: 6.39 s
```

In [79]:

```
%%time
load_store_country_chi(
    topic="sunrise", source="instagram", metric="userdays", store_csv = True)
```

```
CPU times: user 6.46 s, sys: 112 ms, total: 6.57 s
Wall time: 7.03 s
```

In [80]:

```
%%time
load_store_country_chi(
    topic="sunset", source="instagram", metric="usercount", store_csv = True)
```

```
CPU times: user 5.7 s, sys: 132 ms, total: 5.83 s
Wall time: 6.37 s
```

In [81]:

```
load_store_country_chi(
    topic="sunset", source="instagram", metric="userdays", store_csv = True)
```

# Visualization¶

Plot Interactive Country chi maps (diverging colormap).

**ToDo**: The code below is very similar to the one in `03_chimaps.ipynb` and `06_semantics.ipynb`. Can be reduced significantly with deduplication and refactoring.

Prepare methods. The first one is needed to plot country polygons in `hv` using geoviews `gv.Polygons`. The syntax is very similar to `convert_gdf_to_gvimage()`. There are further slight adjustments necessary to other methods, which are copied from previous notebooks.

We also need to create a pooled classification, meaning that all values for all maps are used to create scheme breaks, and then those global breaks are used across maps to classify bins. This allows use to compare maps.

In [82]:

```
def convert_gdf_to_gvpolygons(
        poly_gdf: gp.GeoDataFrame, metric: str = METRIC, cat_count: Optional[int] = None, 
        cat_min: Optional[int] = None, cat_max: Optional[int] = None,
        hover_items: Dict[str, str] = None) -> gv.Polygons:
    """Convert GeoDataFrame to gv.polygons using categorized
    metric column as value dimension
    
    Args:
        poly_gdf: A geopandas geodataframe with  
            (projected coordinates) and aggregate metric column
        metric: target column for value dimension.
            "_cat" will be added to retrieve classified values.
        cat_count: number of classes for value dimension
        hover_items: a dictionary with optional names 
            and column references that are included in 
            gv.Image to provide additional information
            (e.g. on hover)
    """
    if cat_count:
        cat_min = 0
        cat_max = cat_count
    else:
        if any([cat_min, cat_max]) is None:
            raise ValueError(
                "Either provide cat_count or cat_min and cat_max.")
    if hover_items is None:
        hover_items_list = []
    else:
        hover_items_list = [
            v for v in hover_items.values()]
    # convert GeoDataFrame to gv.Polygons Layer
    # the first vdim is the value being used 
    # to visualize classes on the map
    # include additional_items (postcount and usercount)
    # to show exact information through tooltip
    gv_layer = gv.Polygons(
        poly_gdf,
        vdims=[
            hv.Dimension(
                f'{metric}_cat', range=(cat_min, cat_max))]
            + hover_items_list,
        crs=crs.Mollweide())
    return gv_layer
```

Derived from `get_classify_image`:

In [83]:

```
def compile_diverging_poly_layer(
        poly_gdf: gp.GeoDataFrame, series_plus: pd.Series,
        series_minus: pd.Series,
        metric: str = "chi_value", responsive: bool = None,
        hover_items: Dict[str, str] = hover_items,
        mask_nonsignificant: bool = False,
        add_notopicdata_label: str = None,
        scheme: str = "HeadTailBreaks",
        cmaps_diverging: Tuple[str] = ("OrRd", "Blues"),
        add_nodata_label: str = "#FFFFFF",
        true_negative: bool = True,
        bounds_plusminus: Tuple[List[str], List[str]] = (None, None),
        schemebreaks_plusminus: Tuple["mc.classifier", "mc.classifier"] = (None, None)):
    """Modified function to get/combine diverging image layer
    
    Additional Args:
        series_plus: Series of values to show on plus y range cmap
        series_minus: Series of values to show on minus y range cmap
        cmaps_diverging: Tuple with plus and minus cmap reference
        bounds: Optional predefined bounds (map comparison)
        scheme_breaks: Optional predefined scheme_breaks (map comparison)
    """
    ##stats = {}
    div_labels: List[Dict[int, str]] = []
    div_cmaps: List[List[str]] = []
    cat_counts: List[int] = []
    div_bounds: List[List[float]] = []
    spare_cats = 0
    plus_offset = 0
    offset = 0
    for ix, series_nan in enumerate([series_plus, series_minus]):
        # classify values for both series
        cmap_name = cmaps_diverging[ix]
        if bounds_plusminus[ix] and schemebreaks_plusminus[ix]:
            # get predifined breaks
            bounds = bounds_plusminus[ix]
            scheme_breaks = schemebreaks_plusminus[ix]
        else:
            # calculate new
            bounds, scheme_breaks = classify_data(
                values_series=series_nan, scheme=scheme)
        div_bounds.append(bounds)
        # assign categories column
        cat_series = scheme_breaks.find_bin(
            np.abs(series_nan.values))
        cat_count = scheme_breaks.k
        color_count = scheme_breaks.k + offset
        cmap_list = get_cmap_list(
            cmap_name, length_n=color_count)
        if ix == 0:
            if add_notopicdata_label:
                # add grey color
                plus_offset = 1
                prepend_color(
                    cmap_list=cmap_list, color_hex=add_notopicdata_label)
            if add_nodata_label:
                # offset
                spare_cats = 1
                prepend_color(
                    cmap_list=cmap_list, color_hex=add_nodata_label)
            cat_count += (offset+plus_offset)
            # nodata label as explicit category in legend
            # values will not be rendered on map (cat = nan):
            # increment cat count, but not series
            cat_series += (offset+plus_offset+spare_cats)
        if ix == 1:
            cat_count += offset
            # cat labels always prepended with minus sign (-)
            cat_series = np.negative(cat_series)
            # offset
            cat_series -= (1+offset)
        cat_counts.append(cat_count)
        div_cmaps.append(cmap_list)
        # assign categories
        poly_gdf.loc[series_nan.index, f'{metric}_cat'] = cat_series.astype(str)
    # general application:
    # assign special cat labels to data
    assign_special_categories(
        grid=poly_gdf, series_plus=series_plus, series_minus=series_minus,
        metric=metric, add_notopicdata_label=add_notopicdata_label,
        add_underrepresented_label=offset,
        add_nodata_label=add_nodata_label, mask_nonsignificant=mask_nonsignificant)
    # clean na() values !important
    mask = (poly_gdf[f'{metric}_cat'].isna())
    poly_gdf.loc[
        mask,
        f'{metric}_cat'] = '0'
    # poly_gdf[f'{metric}_cat'] = poly_gdf[f'{metric}_cat'].astype(str)
    # allow label cat to be shown on hover
    poly_gdf.loc[poly_gdf.index, f"{metric}_cat_label"] = poly_gdf[f"{metric}_cat"]
    hover_items['Label Cat'] = 'chi_value_cat_label'
    # special categories
    kwargs = {
        "mask_nonsignificant":mask_nonsignificant,
        "add_nodata_label":add_nodata_label,
        "add_notopicdata_label":add_notopicdata_label,
        "true_negative":true_negative,
        "offset":offset
    }
    label_dict = create_diverging_labels(
        div_bounds, **kwargs)
    # adjust tick positions, 
    # due to additional no_data_label
    # shown in legend only
    if add_nodata_label:
        label_dict = update_tick_positions(label_dict)
    # reverse colors of minus cmap
    # div_cmaps[1].reverse()
    # combine cmaps
    cmap_nodata_list = div_cmaps[1] + div_cmaps[0]
    cmap = colors.ListedColormap(cmap_nodata_list)
    # create gv.image layer from gdf
    gv_poly = convert_gdf_to_gvpolygons(
            poly_gdf=poly_gdf,
            metric=metric, cat_min=-cat_counts[1],
            cat_max=cat_counts[0],
            hover_items=hover_items)
    poly_layer = apply_polylayer_opts(
        gv_poly=gv_poly, cmap=cmap, label_dict=label_dict,
        responsive=responsive, hover_items=hover_items)
    return poly_layer
```

In [84]:

```
def apply_polylayer_opts(
    gv_poly: gv.Polygons, cmap: colors.ListedColormap,
    label_dict: Dict[str, str], responsive: bool = None,
    hover_items: Dict[str, str] = None) -> gv.Image:
    """Apply geoviews polygons layer opts

    Args:
        gv_poly: A classified gv.Polygons layer
        responsive: Should be True for interactive HTML output.
        hover_items: additional items to show on hover
        cmap: A matplotlib colormap to colorize values and show as legend.
    """
    if hover_items is None:
        hover_items = {
        'Post Count (estimated)':'postcount_est', 
        'User Count (estimated)':'usercount_est',
        'User Days (estimated)':'userdays_est'}
    color_levels = len(cmap.colors)
    # define additional plotting parameters
    # width of static jupyter map,
    # 360° == 1200px
    width = 1200
    # height of static jupyter map,
    # 360°/2 == 180° == 600px
    height = int(width/2) 
    aspect = None
    # if stored as html,
    # override values
    if responsive:
        width = None
        height = None
    # define width and height as optional parameters
    # only used when plotting inside jupyter
    optional_kwargs = dict(width=width, height=height)
    # compile only values that are not None into kwargs-dict
    # by using dict-comprehension
    optional_kwargs_unpack = {
        k: v for k, v in optional_kwargs.items() if v is not None}
    # prepare custom HoverTool
    tooltips = get_custom_tooltips(
        hover_items)
    hover = HoverTool(tooltips=tooltips)
    # get tick positions from label dict keys
    ticks = [key for key in sorted(label_dict)]
    # create image layer
    return gv_poly.sort('chi_value_cat').opts(
            show_legend=True,
            color_levels=color_levels,
            cmap=cmap,
            colorbar=True,
            line_color='grey',
            line_width=0.3,
            clipping_colors={'NaN': 'transparent'},
            colorbar_opts={
                # 'formatter': formatter,
                'major_label_text_align':'left',
                'major_label_overrides': label_dict,
                'ticker': FixedTicker(
                    ticks=ticks),
                },
            tools=[hover],
            # optional unpack of width and height
            **optional_kwargs_unpack
        )
```

In [85]:

```
def combine_gv_layers(
        poly_layer: gv.Polygons, edgecolor: str = 'black',
        fill_color: str = '#dbdbdb', alpha: float = 0.15) -> gv.Overlay:
    """Combine layers into single overlay and set global plot options"""
    # fill_color = '#479AD4'
    # fill_color = '#E9EDEC'
    gv_layers = []
    gv_layers.append(
        gf.land.opts(
            alpha=alpha, fill_color=fill_color, line_width=0.5))
    gv_layers.append(
        poly_layer)
    return gv.Overlay(gv_layers)
```

In [86]:

```
from typing import Any
def get_gobal_scheme_breaks(
    series: pd.Series, scheme: str = "HeadTailBreaks") -> Tuple[Any, Any]:
        bounds, scheme_breaks = classify_data(
            values_series=series, scheme=scheme)
        return bounds, scheme_breaks
```

In [87]:

```
def get_combine_plus_minus(list_df: List[pd.DataFrame], mask_nonsignificant: bool = False,
    metric: str = "chi_value") -> Tuple[pd.Series, pd.Series]:
    """Merge (concat) all metric values of all dataframes to a single series. Returns
    two merged series for positive and negative values"""
    base_kwargs = {
        "mask_nonsignificant":mask_nonsignificant,
        "metric":metric}
    combined_plusminus = []
    mask_kwargs = ["mask_negative", "mask_positive"]
    for ix, mask_kwarg in enumerate(mask_kwargs):
        # merge two dictionaries
        kwargs = base_kwargs | { mask_kwarg:True }
        series_combined = None
        for df in list_df:
            df_copy = df.copy()
            masked_series = mask_series(
                grid=df_copy,
                **kwargs)
            if series_combined is None:
                series_combined = masked_series
                continue
            series_combined = pd.concat(
                [series_combined, masked_series], axis=0, 
                ignore_index=True)
        combined_plusminus.append(series_combined)
    return (combined_plusminus[0], combined_plusminus[1])
```

In [88]:

```
def plot_diverging_poly(poly_gdf: gp.GeoDataFrame, title: str,
    hover_items: Dict[str, str] = {
        'Post Count (estimated)':'postcount_est', 
        'User Count (estimated)':'usercount_est',
        'User Days (estimated)':'userdays_est'},
    mask_nonsignificant: bool = False,
    scheme: str = "HeadTailBreaks",
    cmaps_diverging: Tuple[str] = ("OrRd", "Blues"),
    store_html: str = None,
    plot: Optional[bool] = True,
    output: Optional[str] = OUTPUT,
    nodata_color: str = "#FFFFFF",
    notopicdata_color: str = None,
    true_negative: bool = True,
    bounds_plusminus: Tuple[List[str], List[str]] = (None, None),
    schemebreaks_plusminus: Tuple["mc.classifier", "mc.classifier"] = (None, None)) -> gv.Overlay:
    """Plot interactive map with holoviews/geoviews renderer

    Args:
        grid: A geopandas geodataframe with indexes x and y 
            (projected coordinates) and aggregate metric column
        metric: target column for aggregate. Default: postcount.
        store_html: Provide a name to store figure as interactive HTML.
        title: Title of the map
        cmaps_diverging: Tuple for colormaps to use.
        hover_items: additional items to show on hover
        mask_nonsignificant: transparent bins if significant column == False
        scheme: The classification scheme to use. Default "HeadTailBreaks".
        cmap: The colormap to use. Default "OrRd".
        plot: Prepare gv-layers to be plotted in notebook.
        true_negative: Whether "minus" values should show "-" in legend.
    """
    # work on a shallow copy,
    # to not modify original dataframe
    poly_gdf_plot = poly_gdf.copy()
    poly_gdf_plot['SU_A3'] = poly_gdf_plot.index
     # check if all additional items are available
    for key, item in list(hover_items.items()):
        if item not in poly_gdf_plot.columns:
            hover_items.pop(key)
    # chi layer opts
    base_kwargs = {
        "mask_nonsignificant":mask_nonsignificant,
        "metric":"chi_value",
    }
    # classify based on positive and negative chi
    series_plus = mask_series(
        grid=poly_gdf_plot,
        mask_negative=True,
        **base_kwargs)
    series_minus = mask_series(
        grid=poly_gdf_plot,
        mask_positive=True, 
        **base_kwargs)
    # global plotting options for value layer
    layer_opts = {
        "poly_gdf":poly_gdf_plot,
        "series_plus":series_plus,
        "series_minus":series_minus,
        "responsive":False,
        "scheme":scheme,
        "hover_items":hover_items,
        "cmaps_diverging":cmaps_diverging,
        "add_nodata_label":nodata_color,
        "add_notopicdata_label":notopicdata_color,
        "true_negative":true_negative,
        "bounds_plusminus":bounds_plusminus,
        "schemebreaks_plusminus":schemebreaks_plusminus
    }    
    # global plotting options for all layers (gv.Overlay)
    gv_opts = {
        "bgcolor":None,
        # "global_extent":True,
        "projection":crs.Mollweide(),
        "responsive":False,
        "data_aspect":1, # maintain fixed aspect ratio during responsive resize
        "hooks":[set_active_tool],
        "title":title
    }
    # get global plotting bounds/breaks for consistent scheme across all maps
    
    # plot responsive (html) or non-responsive (interactive)
    if plot:
        # get classified gv poly layer
        poly_layer = compile_diverging_poly_layer(
            **layer_opts, **base_kwargs)
        gv_layers = combine_gv_layers(
            poly_layer, fill_color=nodata_color, alpha=0.5)
    if store_html:
        layer_opts["responsive"] = True
        poly_layer = compile_diverging_poly_layer(
            **layer_opts, **base_kwargs)
        responsive_gv_layers = combine_gv_layers(
            poly_layer, fill_color=nodata_color, alpha=0.5)
        gv_opts["responsive"] = True
        export_layers = responsive_gv_layers.opts(**gv_opts)
        hv.save(
            export_layers,
            output / f"html" / f'{store_html}.html', backend='bokeh')
        if WEB_DRIVER:
            # store also as svg
            p =  hv.render(export_layers, backend='bokeh')
            p.output_backend = "svg"
            export_svgs(
                p, filename=output / f"svg{km_size_str}" / f'{store_html}.svg',
                webdriver=WEB_DRIVER)
    if not plot:
        return
    gv_opts["responsive"] = False
    return gv_layers.opts(**gv_opts)
```

In [89]:

```
hover_items = { 
    'User Count (est)':'usercount_est',
    'Country Code':'SU_A3'}
hover_items_chi = {
    f'Total {METRIC_NAME_REF[CHI_COLUMN]}':f'{CHI_COLUMN}_expected',
    'Chi-value':'chi_value',
    'Chi-significant':'significant',
    'Label Cat':'chi_value_cat_label'}
hover_items.update(hover_items_chi)
```

Process data

In [90]:

```
world_flickr_sunset = load_store_country_chi(
    topic="sunset", source="flickr", metric="usercount", return_gdf=True)
```

In [91]:

```
world_flickr_sunrise = load_store_country_chi(
    topic="sunrise", source="flickr", metric="usercount", return_gdf=True)
```

Process Instagram data:

- expected based on random 20M (`flickrexpected = False`)
- or on flickr totals (`flickrexpected = True`)

In [92]:

```
flickrexpected = False
world_instagram_sunset = load_store_country_chi(
    topic="sunset", source="instagram", metric="usercount", flickrexpected=flickrexpected, return_gdf=True)
```

In [93]:

```
world_instagram_sunrise = load_store_country_chi(
    topic="sunrise", source="instagram", metric="usercount", flickrexpected=flickrexpected, return_gdf=True)
```

Set plotting args

In [94]:

```
kwargs = {
    "hover_items":hover_items,
    "cmaps_diverging":("OrRd", "Blues"),
    "scheme":"Quantiles",
    "mask_nonsignificant":False
}
```

Get global scheme breaks/classes

- the same class breaks will be applied to all *positive chi* values across all four maps
- the same class breaks will be applied to all *negative chi* values across all four maps

In [95]:

```
# 1. combine all plus and minus series
plus_series_merged, minus_series_merged = get_combine_plus_minus(
    [world_flickr_sunset, world_flickr_sunrise, world_instagram_sunset, world_instagram_sunrise])
# 2. get classes /breaks for combined series
plus_bounds, plus_scheme_breaks = get_gobal_scheme_breaks(plus_series_merged, scheme=kwargs.get("scheme"))
minus_bounds, minus_scheme_breaks = get_gobal_scheme_breaks(minus_series_merged, scheme=kwargs.get("scheme"))
kwargs["bounds_plusminus"] = (plus_bounds, minus_bounds)
kwargs["schemebreaks_plusminus"] = (plus_scheme_breaks, minus_scheme_breaks)
```

Print Legend labels and eval scheme breaks:

In [96]:

```
print(f'Plus: {plus_bounds}')
print(f'Plus scheme breaks: {plus_scheme_breaks}')
print(f'Minus: {minus_bounds}')
print(f'Minus scheme breaks: {minus_scheme_breaks}')
```

```
Plus: ['0', '3', '7', '13', '21', '31', '46', '74', '132', '700']
Plus scheme breaks: Quantiles               

    Interval       Count
------------------------
[  0.01,   3.15] |    69
(  3.15,   7.45] |    68
(  7.45,  12.64] |    69
( 12.64,  20.90] |    68
( 20.90,  31.33] |    69
( 31.33,  46.47] |    68
( 46.47,  74.34] |    69
( 74.34, 131.66] |    68
(131.66, 699.59] |    69
Minus: ['0', '3', '7', '11', '15', '21', '29', '46', '88', '709']
Minus scheme breaks: Quantiles               

    Interval       Count
------------------------
[  0.07,   2.82] |    58
(  2.82,   6.63] |    57
(  6.63,  10.53] |    57
( 10.53,  14.97] |    57
( 14.97,  21.15] |    58
( 21.15,  29.02] |    57
( 29.02,  46.01] |    57
( 46.01,  87.92] |    57
( 87.92, 709.02] |    58
```

In [97]:

```
world_instagram_sunset['chi_value'].max()
```

Out[97]:

```
699.5888929612047
```

In [98]:

```
title=f'Chi values (over- and underrepresentation): Flickr "Sunset" {METRIC_NAME_REF[CHI_COLUMN]} (estimated) for Countries, 2007-2018'
pd.set_option('display.max_colwidth', 500)
gv_plot = plot_diverging_poly(
    world_flickr_sunset,
    title=title,
    store_html=f"countries_sunset_flickr_chi_usercount_{kwargs.get('scheme')}", **kwargs)
gv_plot
```

Out[98]:

> QUANTILES will create attractive maps that place an equal number of observations in each class: If you have 30 counties and 6 data classes, you’ll have 5 counties in each class. The problem with quantiles is that you can end up with classes that have very different numerical ranges (e.g., 1-4, 4-9, 9-250).
>
> NATURAL BREAKS is a kind of “optimal” classification scheme that finds class breaks that will minimize within-class variance and maximize between-class differences. One drawback of this approach is each dataset generates a unique classification solution, and if you need to make comparison across maps, such as in an atlas or a series (e.g., one map each for 1980, 1990, 2000) you might want to use a single scheme that can be applied across all of the maps.

Compare to matplotlib rendered plot with single color cmap:

- define plotting parameter

In [99]:

```
base_kwargs = {
    "column":'chi_value',
    "edgecolor":'grey',
    }
all_kwargs = {
    "cmap":'OrRd',
    "linewidth":0.2,
    "legend":True,
    "k":9,
    "scheme":'quantiles'    
    }
hatch_kwargs = {
    "hatch":"///",
    "alpha":0.5,
    "facecolor":"none",
    "linewidth":0
    }
```

- plot in two steps, using linear color gradient and hatch to label underrepresented

In [100]:

```
fig, ax = plt.subplots(1, 1, figsize=(22,28))
world_flickr_sunset.plot(
    ax=ax, **base_kwargs, **all_kwargs)
world_flickr_sunset[world_flickr_sunset['chi_value']<0].plot(
    ax=ax, **base_kwargs, **hatch_kwargs)
ax.set_title(title)
```

Out[100]:

```
Text(0.5, 1.0, 'Chi values (over- and underrepresentation): Flickr "Sunset" User Count (estimated) for Countries, 2007-2018')
```

In [101]:

```
title=f'Chi values (over- and underrepresentation): Flickr "Sunrise" {METRIC_NAME_REF[CHI_COLUMN]} (estimated) for Countries, 2007-2018'
gv_plot = plot_diverging_poly(
    world_flickr_sunrise,
    title=title,
    store_html=f"countries_sunrise_flickr_chi_usercount_{kwargs.get('scheme')}", **kwargs)
gv_plot
```

Out[101]:

Compare to matplotlib rendered plot (without synced/pooled cmap/classes):

In [102]:

```
fig, ax = plt.subplots(1, 1, figsize=(22,28))
world_flickr_sunrise.plot(
    ax=ax, **base_kwargs, **all_kwargs)
world_flickr_sunrise[world_flickr_sunrise['chi_value']<0].plot(
    ax=ax, **base_kwargs, **hatch_kwargs)
ax.set_title(title)
```

Out[102]:

```
Text(0.5, 1.0, 'Chi values (over- and underrepresentation): Flickr "Sunrise" User Count (estimated) for Countries, 2007-2018')
```

In [103]:

```
title=f'Chi values (over- and underrepresentation): Instagram "Sunset" {METRIC_NAME_REF[CHI_COLUMN]} (estimated) for Countries, Aug-Dec 2017'
gv_plot = plot_diverging_poly(
    world_instagram_sunset,
    title=title,
    store_html=f"countries_sunset_instagram_chi_usercount_{kwargs.get('scheme')}{'_flickrexpected' if flickrexpected else ''}", **kwargs)
gv_plot
```

Out[103]:

Compare to matplotlib rendered plot (without synced/pooled cmap/classes):

In [104]:

```
fig, ax = plt.subplots(1, 1, figsize=(22,28))
world_instagram_sunset.plot(
    ax=ax, **base_kwargs, **all_kwargs)
world_instagram_sunset[world_instagram_sunset['chi_value']<0].plot(
    ax=ax, **base_kwargs, **hatch_kwargs)
ax.set_title(title)
```

Out[104]:

```
Text(0.5, 1.0, 'Chi values (over- and underrepresentation): Instagram "Sunset" User Count (estimated) for Countries, Aug-Dec 2017')
```

In [105]:

```
title=f'Chi values (over- and underrepresentation): Instagram "Sunrise" {METRIC_NAME_REF[CHI_COLUMN]} (estimated) for Countries, Aug-Dec 2017'
gv_plot = plot_diverging_poly(
    world_instagram_sunrise,
    title=title,
    store_html=f"countries_sunrise_instagram_chi_usercount_{kwargs.get('scheme')}{'_flickrexpected' if flickrexpected else ''}", **kwargs)
gv_plot
```

Out[105]:

Compare to matplotlib rendered plot (without synced/pooled cmap/classes):

In [106]:

```
fig, ax = plt.subplots(1, 1, figsize=(22,28))
world_instagram_sunrise.plot(
    ax=ax, **base_kwargs, **all_kwargs)
world_instagram_sunrise[world_instagram_sunrise['chi_value']<0].plot(
    ax=ax, **base_kwargs, **hatch_kwargs)
ax.set_title(title)
```

Out[106]:

```
Text(0.5, 1.0, 'Chi values (over- and underrepresentation): Instagram "Sunrise" User Count (estimated) for Countries, Aug-Dec 2017')
```

world\_flickr\_sunset### Pooled Classification

Create the combined figure for the paper, based on pooled classification.

First, combine values into single dataframe.

In [107]:

```
world = world_flickr_sunset
world.rename(columns={'chi_value':'flickrsunset'}, inplace=True)
world.rename(columns={'significant':'significant_flickrsunset'}, inplace=True)
world.rename(columns={'usercount_est':'usercount_est_flickrsunset'}, inplace=True)
world.rename(columns={'usercount_est_expected':'usercount_est_expected_flickr'}, inplace=True)
world['flickrsunset'] = world['flickrsunset'].astype('float')
world['flickrsunrise'] = world_flickr_sunrise['chi_value'].astype('float')
world['usercount_est_flickrsunrise'] = world_flickr_sunrise['usercount_est'].astype('float')
world['significant_flickrsunrise'] = world_flickr_sunrise['significant']
world['instagramsunset'] = world_instagram_sunset['chi_value'].astype('float')
world['instagramsunrise'] = world_instagram_sunrise['chi_value'].astype('float')
world['significant_instagramsunrise'] = world_instagram_sunrise['significant']
world['usercount_est_instagramsunrise'] = world_instagram_sunrise['usercount_est'].astype('float')
world['usercount_est_instagramsunset'] = world_instagram_sunset['usercount_est'].astype('float')
world['usercount_est_expected_instagram'] = world_instagram_sunset['usercount_est_expected'].astype('float')
world['significant_instagramsunset'] = world_instagram_sunset['significant']
world.fillna(0, inplace=True)
```

Specify the columns to be used for pooled classification

In [108]:

```
submaps = ["flickrsunrise","flickrsunset","instagramsunrise","instagramsunset"]
# Create pooled classification
k_classes = 18
pooled = mc.Pooled(
    world[submaps], classifier='Quantiles', k=k_classes
)
```

In [109]:

```
pooled.global_classifier.bins
```

Out[109]:

```
array([-7.21350945e+01, -3.76494048e+01, -2.40401678e+01, -1.65053352e+01,
       -1.07166104e+01, -5.81677972e+00, -1.77537441e+00,  0.00000000e+00,
        1.08146617e-01,  3.19047689e+00,  7.61108909e+00,  1.27635435e+01,
        2.10064008e+01,  3.15454240e+01,  4.68749952e+01,  7.44087720e+01,
        1.32223741e+02,  6.99588893e+02])
```

In [110]:

```
title_ref = {
    "flickrsunset":f'Flickr Sunset',
    "flickrsunrise":f'Flickr Sunrise',
    "instagramsunset":f'Instagram Sunset',
    "instagramsunrise":f'Instagram Sunrise'
}
```

The colormap that is used in 100x100km grids (`get_cmap_list()` in `02_visualization.ipynb`) results in two quite dark colors for max (minus and plus).

Below, this will be slighlty adapted, with a lighter color. Also, instead of blue, use purple, to not confuse sunset-sunrise meaning of blue/red in other graphics.

In [111]:

```
def get_diverging_colormap(cmap_diverging:Tuple[str, str], color_count: int = 9):
    """Create a diverging colormap from two existing with k classes"""
    div_cmaps: List[List[str]] = []
    for ix, cmap_name in enumerate(cmap_diverging):
        if ix == 1:
            # offset by 1, to darken first color a bit
            cmap =  plt.cm.get_cmap(cmap_name, color_count+1)
        cmap = plt.cm.get_cmap(cmap_name, color_count)
        cmap_list = get_hex_col(cmap)
        if ix == 0:
            # set first color as white
            cmap_list[0] = '#ffffff'
        if ix == 1:
            # remove first (too light) color
            cmap_list.pop(0)
        div_cmaps.append(cmap_list)
    div_cmaps[1] = list(reversed(div_cmaps[1]))
    cmap_nodata_list = div_cmaps[1] + div_cmaps[0]
    return colors.ListedColormap(cmap_nodata_list)
```

Experiment with values below.

In [112]:

```
# cmaps_diverging: Tuple[str] = ("OrRd", "Purples")
cmaps_diverging: Tuple[str] = ("OrRd", "Blues")
cmap = get_diverging_colormap(cmaps_diverging, color_count=(k_classes/2)+1)
```

In [113]:

```
len(cmap.colors)
```

Out[113]:

```
19
```

Preview colormap (also useful for legend).

In [114]:

```
tools.display_hex_colors(cmap.colors, as_id=True)
```

|  | 00000 | 00001 | 00002 | 00003 | 00004 | 00005 | 00006 | 00007 | 00008 | 00009 | 00010 | 00011 | 00012 | 00013 | 00014 | 00015 | 00016 | 00017 | 00018 |
| --- | --- | --- | --- | --- | --- | --- | --- | --- | --- | --- | --- | --- | --- | --- | --- | --- | --- | --- | --- |
| 0 |  |  |  |  |  |  |  |  |  |  |  |  |  |  |  |  |  |  |  |

Use `False` for legend below to store figure.

In [115]:

```
import matplotlib as mpl
# adjust hatch width
mpl.rcParams['hatch.linewidth'] = 2

all_kwargs = {
    "cmap":cmap,
    "edgecolor":'grey',
    "linewidth":0.2,
    "legend":True,   
    }
hatch_kwargs = {
    "hatch":"///",
    "alpha":1.0,
    "edgecolor":"white",
    "facecolor":"none",
    "linewidth":0,
    }
```

Total number of countries:

In [116]:

```
len(world)
```

Out[116]:

```
307
```

Count the number of total and non-significant countries for Flickr sunrise:

In [117]:

```
len(world[world["significant_flickrsunrise"]==False])
```

Out[117]:

```
65
```

.. and Flickr sunset:

In [118]:

```
len(world[world["significant_flickrsunset"]==False])
```

Out[118]:

```
72
```

Count the number of total and non-significant countries for Instagram sunset:

In [119]:

```
len(world[world["significant_instagramsunset"]==False])
```

Out[119]:

```
52
```

Count the number of total and non-significant countries for Instagram sunrise:

In [120]:

```
len(world[world["significant_instagramsunrise"]==False])
```

Out[120]:

```
59
```

Print the top 10 non-significant countries sorted by `usercount_est`(decending) for **Flickr/Sunset**:

In [121]:

```
world[world["significant_flickrsunset"]==False].drop(
    world.columns.difference(
        ["usercount_est_expected_flickr", "usercount_est_flickrsunset", "significant_flickrsunset"]),
    axis=1,
    inplace=False).sort_values(
        ["usercount_est_flickrsunset"], ascending=False).head(10)
```

Out[121]:

|  | usercount\_est\_expected\_flickr | usercount\_est\_flickrsunset | significant\_flickrsunset |
| --- | --- | --- | --- |
| SU\_A3 |  |  |  |
| CUB | 11826.0 | 1108.0 | False |
| TZA | 5612.0 | 515.0 | False |
| BGD | 5229.0 | 455.0 | False |
| PMD | 4332.0 | 373.0 | False |
| OMN | 3289.0 | 317.0 | False |
| KWT | 2745.0 | 232.0 | False |
| RUC | 2398.0 | 225.0 | False |
| PAZ | 2424.0 | 212.0 | False |
| ECG | 2115.0 | 198.0 | False |
| IMN | 1704.0 | 151.0 | False |

Print the top 10 non-significant countries sorted by `usercount_est`(decending) for **Instagram/Sunset**:

In [122]:

```
world[world["significant_instagramsunset"]==False].drop(
    world.columns.difference(
        ["usercount_est_expected_instagram", "usercount_est_instagramsunset", "significant_instagramsunset"]),
    axis=1,
    inplace=False).sort_values(
        ["usercount_est_instagramsunset"], ascending=False).head(10)
```

Out[122]:

|  | usercount\_est\_instagramsunset | usercount\_est\_expected\_instagram | significant\_instagramsunset |
| --- | --- | --- | --- |
| SU\_A3 |  |  |  |
| NOR | 24498.0 | 83627.0 | False |
| DOM | 6327.0 | 21275.0 | False |
| ECD | 4655.0 | 15416.0 | False |
| ARM | 1266.0 | 4417.0 | False |
| GHA | 552.0 | 1765.0 | False |
| LBY | 277.0 | 852.0 | False |
| DNB | 268.0 | 942.0 | False |
| AIA | 136.0 | 398.0 | False |
| DMA | 106.0 | 434.0 | False |
| BEN | 85.0 | 321.0 | False |

These are all pretty small countries or islands. Lets compare the area/size of non-significant cpuntries

In [123]:

```
area_instagramsunset_nonsignificant = \
    world.loc[(world["significant_instagramsunset"]==False), 'geometry'].area.sum()
area_instagramsunset_significant = \
    world.loc[(world["significant_instagramsunset"]==True), 'geometry'].area.sum()
percentage_sunset = area_instagramsunset_nonsignificant/(area_instagramsunset_significant/100)
```

In [124]:

```
print(f'{area_instagramsunset_significant/1000000:,.0f} km² significant')
print(f'{area_instagramsunset_nonsignificant/1000000:,.0f} km² non-significant')
print(f'{percentage_sunset:.0f}%')
```

```
143,807,670 km² significant
2,980,617 km² non-significant
2%
```

In [125]:

```
fig, axs = plt.subplots(2, 2, figsize=(22, 22))

# Flatten the array of axis so you can loop over
# in one dimension
axs = axs.flatten()
# Loop over each map
topic = "flickr"
for i, col in enumerate(submaps):
    if i >= 2:
        topic = "instagram"
    if (i % 2) == 0:
        topicsel = f'{topic}sunset'
    else:
        topicsel = f'{topic}sunrise'
    world.plot(
        col,                  # Year to plot
        scheme='UserDefined', # Use our own bins
        classification_kwds={ # Use global bins
            'bins': pooled.global_classifier.bins
        }, 
        ax=axs[i],             # Plot on the corresponding axis
        **all_kwargs
    )
    # hatch non-significant
    world[world[f"significant_{topicsel}"]==False].plot(
        ax=axs[i], **hatch_kwargs)
    # Remove axis
    axs[i].set_axis_off()
    # Name the subplot with the name of the column
    if all_kwargs.get("legend"):
        axs[i].set_title(title_ref.get(col))
fig.subplots_adjust(hspace=-0.7)
# Tight layout to better use space
plt.tight_layout()
# Display figure
plt.show()
if not all_kwargs.get("legend"):
    fig.savefig(
        OUTPUT / "figures" / "country_chi.png", dpi=300, format='PNG',
        bbox_inches='tight', pad_inches=1, facecolor="white")
    # also save as svg
    fig.savefig(
        OUTPUT / "svg" / "country_chi.svg", format='svg',
        bbox_inches='tight', pad_inches=1, facecolor="white")
```

# Close DB connection & Create notebook HTML¶

In [119]:

```
DB_CONN.close ()
```

In [120]:

```
!jupyter nbconvert --to html_toc \
    --output-dir=../out/html ./05_countries.ipynb \
    --template=../nbconvert.tpl \
    --ExtractOutputPreprocessor.enabled=False >&- 2>&- # create single output file
```

Copy single HTML file to resource folder

In [121]:

```
!cp ../out/html/05_countries.html ../resources/html/
```

In [ ]:

```

```
