# Supplementary material for: From sunrise to sunset: Exploring landscape preference through global reactions to ephemeral events captured in georeferenced social media
Source: PLoS One. 2023 Feb 22;18(2):e0280423. doi: 10.1371/journal.pone.0280423 (PMC9946259; doi:10.1371/journal.pone.0280423)
Supplement: S6 File — (HTML) [file pone.0280423.s006.html]

06\_semantics


# Term frequency-inverse document frequency (TFIDF) and Cosine Similarity¶

*Alexander Dunkel, TU Dresden, Institute of Cartography; Maximilian Hartmann and Ross Purves Universität Zürich (UZH), Geocomputation;*

---

•••

Out[1]:

Last updated: Jan-17-2023, Carto-Lab Docker Version 0.9.0

Visualization of TFIDF and Cosine Similarity Values

The values loaded here have been generated outside Jupyter, in a separate process. This notebook only visualizes data.

# Preparations¶

## Load dependencies¶

We continue from notebook `05_countries.ipynb`, importing all previously defined methods and top level variables.

In [2]:

```
import sys
from pathlib import Path
module_path = str(Path.cwd().parents[0] / "py")
if module_path not in sys.path:
    sys.path.append(module_path)
# import all previous chained notebooks
from _05_countries import *
```

```
Chromedriver loaded. Svg output enabled.
```

Activate autoreload of changed python files:

In [3]:

```
%load_ext autoreload
%autoreload 2
```

## Load aggregate topic data¶

Data is stored as aggregate HLL data (postcount) for each term.

In [4]:

```
root = Path.cwd().parents[1] / "00_topic_data"
TERMS_FLICKR_TFIDF = root / "20210202_FLICKR_SUNSET_random_country_tf_idf.csv"
TERMS_FLICKR_COSINE = root / "20211029_FLICKR_SUNSET_random_country_cosine_similarity_binary.csv"
```

Some statistics for these files:

In [5]:

```
%%time
data_files = {
    "TERMS_FLICKR_TFIDF":TERMS_FLICKR_TFIDF,
    "TERMS_FLICKR_COSINE":TERMS_FLICKR_COSINE,
    }
tools.display_file_stats(data_files)
```

| name | TERMS\_FLICKR\_TFIDF | TERMS\_FLICKR\_COSINE |
| --- | --- | --- |
| size | 57.85 KB | 974.43 KB |
| records | 226 | 226 |

```
CPU times: user 33.9 ms, sys: 244 µs, total: 34.1 ms
Wall time: 104 ms
```

#### Load Cosine Similarity¶

Get as pandas dataframe

In [6]:

```
def load_cosine_df(csv: Path = TERMS_FLICKR_COSINE) -> pd.DataFrame:
    """Load CSV with cosine similarity values per country"""
    df = pd.read_csv(csv, encoding='utf-8', skiprows=0, index_col=0)
    # Since this is a matrix of similarity values, 
    # set index = column names and skip first row (header)
    df.columns = df.index
    return df
```

In [7]:

```
df_cos = load_cosine_df()
```

In [8]:

```
df_cos.head()
```

Out[8]:

|  | BFR | INX | CHE | IDN | USB | ITX | ZAX | MEX | CAN | ENG | ... | CPV | AZE | HND | MDA | ALD | INA | BLM | LIE | ITP | GUF |
| --- | --- | --- | --- | --- | --- | --- | --- | --- | --- | --- | --- | --- | --- | --- | --- | --- | --- | --- | --- | --- | --- |
| BFR | 1.000000 | 0.176804 | 0.194586 | 0.192786 | 0.088246 | 0.138522 | 0.201482 | 0.175484 | 0.164773 | 0.118088 | ... | 0.101952 | 0.097359 | 0.125799 | 0.095063 | 0.082795 | 0.105904 | 0.074893 | 0.087826 | 0.051295 | 0.105382 |
| INX | 0.176804 | 1.000000 | 0.166689 | 0.185919 | 0.109039 | 0.142907 | 0.187739 | 0.162928 | 0.166713 | 0.138435 | ... | 0.074961 | 0.070127 | 0.095374 | 0.066170 | 0.060736 | 0.087176 | 0.050504 | 0.058790 | 0.036975 | 0.069739 |
| CHE | 0.194586 | 0.166689 | 1.000000 | 0.171150 | 0.096301 | 0.157435 | 0.178351 | 0.159385 | 0.164147 | 0.122183 | ... | 0.080351 | 0.072332 | 0.099305 | 0.070520 | 0.063698 | 0.077771 | 0.054618 | 0.074115 | 0.043080 | 0.083936 |
| IDN | 0.192786 | 0.185919 | 0.171150 | 1.000000 | 0.085915 | 0.127157 | 0.193810 | 0.171063 | 0.156392 | 0.112681 | ... | 0.096579 | 0.087764 | 0.118133 | 0.083325 | 0.078152 | 0.099819 | 0.063213 | 0.075742 | 0.049091 | 0.093572 |
| USB | 0.088246 | 0.109039 | 0.096301 | 0.085915 | 1.000000 | 0.110259 | 0.091638 | 0.091926 | 0.130047 | 0.143619 | ... | 0.026635 | 0.023089 | 0.037211 | 0.023858 | 0.019907 | 0.025114 | 0.018317 | 0.020239 | 0.014332 | 0.026182 |

5 rows × 225 columns

#### Load TFIDF¶

In [9]:

```
def load_tfidf_df(csv: Path = TERMS_FLICKR_TFIDF) -> pd.DataFrame:
    """Load CSV with TFIDF ranking for country"""
    df = pd.read_csv(csv, encoding='utf-8', header=0, index_col=0)
    return df
```

In [10]:

```
df_tfidf = load_tfidf_df()
```

In [11]:

```
df_tfidf.head()
```

Out[11]:

|  | TERM\_1 | TF\_IDF\_1 | TERM\_2 | TF\_IDF\_2 | TERM\_3 | TF\_IDF\_3 | TERM\_4 | TF\_IDF\_4 | TERM\_5 | TF\_IDF\_5 | ... | TERM\_16 | TF\_IDF\_16 | TERM\_17 | TF\_IDF\_17 | TERM\_18 | TF\_IDF\_18 | TERM\_19 | TF\_IDF\_19 | TERM\_20 | TF\_IDF\_20 |
| --- | --- | --- | --- | --- | --- | --- | --- | --- | --- | --- | --- | --- | --- | --- | --- | --- | --- | --- | --- | --- | --- |
| COUNTRY\_CODE |  |  |  |  |  |  |  |  |  |  |  |  |  |  |  |  |  |  |  |  |  |
| ABW | sunset | 237.04 | beach | 81.75 | ocean | 75.27 | sun | 71.35 | palm | 69.74 | ... | sand | 40.54 | and | 36.71 | arubasunset | 36.53 | boats | 36.00 | island | 34.75 |
| ACA | sunset | 103.00 | heights | 31.31 | shirley | 31.29 | english | 27.28 | clouds | 25.68 | ... | bay | 16.95 | shirleyheights | 16.34 | englishharbour | 16.28 | from | 16.18 | this | 16.05 |
| AFG | sunset | 124.64 | sun | 27.91 | mountains | 24.86 | clouds | 21.84 | over | 20.47 | ... | war | 10.08 | army | 10.08 | evening | 9.93 | shadow | 9.85 | light | 9.85 |
| AGO | sunset | 89.23 | sun | 28.24 | sky | 20.02 | the | 18.21 | landscape | 16.94 | ... | nature | 10.54 | okavango | 10.30 | near | 10.30 | luanda | 9.90 | and | 9.76 |
| AIA | sunset | 28.30 | caribbean | 11.88 | ocean | 7.87 | the | 6.86 | sun | 6.81 | ... | beautiful | 4.84 | landscape | 4.84 | sea | 4.04 | anguilla | 3.98 | our | 3.94 |

5 rows × 40 columns

Combine top terms into single column, drop all other columns

In [12]:

```
cols = [f'TERM_{ix}'for ix in range(1,20)]
df_tfidf['tfidf'] = df_tfidf[cols].apply(lambda row: ' '.join(row.values.astype(str)), axis=1)
drop_cols_except(df_tfidf, ['tfidf'])
df_tfidf.head()
```

Out[12]:

|  | tfidf |
| --- | --- |
| COUNTRY\_CODE |  |
| ABW | sunset beach ocean sun palm sky sea clouds the... |
| ACA | sunset heights shirley english clouds sun the ... |
| AFG | sunset sun mountains clouds over sky dusk land... |
| AGO | sunset sun sky the landscape namibia africa ri... |
| AIA | sunset caribbean ocean the sun clouds sky trav... |

## Combine with country shapes¶

### Load country geometries¶

In [13]:

```
def load_country_geom(
    ne_path: Path = NE_PATH, ne_uri: str = NE_URI, ne_filename: str = NE_FILENAME,
    crs_proj: str = CRS_PROJ, country_col: str = COUNTRY_COL) -> gp.GeoDataFrame:
    """Load country geometry and set SU_A3 column as index"""
    world = gp.read_file(
        ne_path / ne_filename.replace(".zip", ".shp"))
    world = world.to_crs(crs_proj)
    columns_keep = ['geometry', country_col, 'ADMIN']
    drop_cols_except(world, columns_keep)
    world.set_index(country_col, inplace=True)
    return world
```

In [14]:

```
world = load_country_geom()
world.head()
```

Out[14]:

|  | ADMIN | geometry |
| --- | --- | --- |
| SU\_A3 |  |  |
| ZWE | Zimbabwe | POLYGON ((2987278.542 -2742733.921, 2979383.40... |
| ZMB | Zambia | POLYGON ((2976200.722 -1924957.705, 2961959.54... |
| YEM | Yemen | MULTIPOLYGON (((5181525.454 2047361.573, 51352... |
| YES | Yemen | POLYGON ((5307347.563 1557616.990, 5313584.814... |
| VNM | Vietnam | MULTIPOLYGON (((10323687.558 1282070.654, 1032... |

This GeoDataFrame can be visualized using interactive Holoviews:

In [15]:

```
gv.Polygons(world, crs=crs.Mollweide())
```

Out[15]:

### Combine data¶

Load world geometry and add cosine value for specific country ref

In [16]:

```
def load_combine(su_a3_ref: str, value_df: pd.DataFrame):
    """Add selected data for country ref"""
    world = load_country_geom()
    world.loc[value_df.index, "cosine"] = value_df[su_a3_ref]
    # Set selected country to NaN, which is always 1 
    # and can therefore be excluded from the classification process
    world.loc[su_a3_ref, "cosine"] = np.nan
    # add tfidf values
    world.loc[df_tfidf.index, "tfidf"] = df_tfidf['tfidf']
    world.tfidf = world.tfidf.fillna('')
    return world
```

### Test¶

Example: UGA (Uganda)

In [17]:

```
world = load_combine("UGA", df_cos)
```

In [18]:

```
world.head()
```

Out[18]:

|  | ADMIN | geometry | cosine | tfidf |
| --- | --- | --- | --- | --- |
| SU\_A3 |  |  |  |  |
| ZWE | Zimbabwe | POLYGON ((2987278.542 -2742733.921, 2979383.40... | 0.205237 | sunset sun lake zambezi river the kariba afric... |
| ZMB | Zambia | POLYGON ((2976200.722 -1924957.705, 2961959.54... | 0.204533 | sunset river zambezi sun cruise the africa ove... |
| YEM | Yemen | MULTIPOLYGON (((5181525.454 2047361.573, 51352... | 0.184590 | sunset sky cloud the nature clouds night lands... |
| YES | Yemen | POLYGON ((5307347.563 1557616.990, 5313584.814... | NaN |  |
| VNM | Vietnam | MULTIPOLYGON (((10323687.558 1282070.654, 1032... | 0.132540 | sunset sun sky the vietnam landscape river asi... |

In [19]:

```
fig, ax = plt.subplots(1, 1, figsize=(22,28))
world.plot(
    column='cosine',
    cmap='OrRd',
    ax=ax,
    linewidth=0.2,
    edgecolor='grey',
    legend=True,
    scheme='headtail_breaks')
```

Out[19]:

```
<AxesSubplot:>
```

## Visualize using Holoviews¶

Combine load, combine and plotting functions first.

Prepare methods. The first one is needed to plot country polygons in `hv` using geoviews `gv.Polygons`. The syntax is very similar to `convert_gdf_to_gvimage()`. There are further slight adjustments necessary to other methods, which are copied from previous notebooks.

In [20]:

```
def convert_gdf_to_gvpolygons(
        poly_gdf: gp.GeoDataFrame, metric: str, cat_count: Optional[int] = None, 
        cat_min: Optional[int] = None, cat_max: Optional[int] = None,
        hover_items: Dict[str, str] = None) -> gv.Polygons:
    """Convert GeoDataFrame to gv.polygons using categorized
    metric column as value dimension
    
    Args:
        poly_gdf: A geopandas geodataframe with  
            (projected coordinates) and aggregate metric column
        metric: target column for value dimension.
            "_cat" will be added to retrieve classified values.
        cat_count: number of classes for value dimension
        hover_items: a dictionary with optional names 
            and column references that are included in 
            gv.Image to provide additional information
            (e.g. on hover)
    """
    if cat_count:
        cat_min = 0
        cat_max = cat_count
    else:
        if any([cat_min, cat_max]) is None:
            raise ValueError(
                "Either provide cat_count or cat_min and cat_max.")
    if hover_items is None:
        hover_items_list = []
    else:
        hover_items_list = [
            v for v in hover_items.values()]
    # convert GeoDataFrame to gv.Polygons Layer
    # the first vdim is the value being used 
    # to visualize classes on the map
    # include additional_items (postcount and usercount)
    # to show exact information through tooltip
    gv_layer = gv.Polygons(
        poly_gdf,
        vdims=[
            hv.Dimension(
                f'{metric}_cat', range=(cat_min, cat_max))]
            + hover_items_list,
        crs=crs.Mollweide())
    return gv_layer
```

In [21]:

```
from _02_visualization import assign_special_categories # use original definition
def get_classify_poly(poly_gdf: gp.GeoDataFrame,
    metric: str = "cosine", responsive: bool = None,
    hover_items: Dict[str, str] = None,
    mask_nonsignificant: bool = False,
    scheme: str = "HeadTailBreaks",
    cmap_name: str = "OrRd",
    cosine_country: str = None):
    """Get and classify gv layer from geodataframe (polygon)

    Args:
        poly_gdf: A geopandas geodataframe with  
            (projected coordinates) and aggregate metric column
        metric: target column for aggregate. Default: cosine.
        responsive: Should be True for interactive HTML output.
        hover_items: additional items to show on hover
        mask_nonsignificant: transparent bins if significant column == False
        scheme: The classification scheme to use. Default "HeadTailBreaks".
        cmap: The colormap to use. Default "OrRd".
    """
    
    # get value series, excluding special categories
    kwargs = {
        "mask_nonsignificant":mask_nonsignificant
    }
    series_nan = mask_series(
        grid=poly_gdf, metric=metric, **kwargs)
    # classify values
    bounds, scheme_breaks = classify_data(
        values_series=series_nan, scheme=scheme)
    # assign categories column
    poly_gdf.loc[series_nan.index, f'{metric}_cat'] = scheme_breaks.find_bin(
        series_nan)
    # set for hover info, after classification
    poly_gdf.loc[cosine_country, "cosine"] = 1.0
    # assign special categories (nodata, not significant, not representative)
    assign_special_categories(
        grid=poly_gdf, values_series=series_nan,
        metric=metric, add_nodata_label=None, **kwargs)
    cat_count = scheme_breaks.k
    cmap_list = get_cmap_list(cmap_name, length_n=cat_count)
    # spare cats are added to legend,
    # but have no representation on the map
    # (e.g. White "No Data" Label)
    # create cmap and labels
    label_dict = create_labels(
        cmap_list, bounds, **kwargs)
    # cosine mod: 
    # make sure that largest label tick is always 1
    max_key = max(label_dict.keys())
    label_dict[max_key] = '1'
    cmap = colors.ListedColormap(cmap_list)
    # create gv.Polygons layer from gdf
    gv_poly = convert_gdf_to_gvpolygons(
            poly_gdf=poly_gdf,
            metric=metric, cat_count=cat_count,
            hover_items=hover_items)
    return gv_poly, cmap, label_dict
```

In [22]:

```
def compile_poly_layer(poly_gdf: gp.GeoDataFrame,
    metric: str = "postcount_est", responsive: bool = None,
    hover_items: Dict[str, str] = None,
    mask_nonsignificant: bool = False,
    scheme: str = "HeadTailBreaks",
    cmap_name: str = "OrRd",
    cosine_country: str = None):
    """Compile geoviews image layer from grid

    Args:
        grid: A geopandas geodataframe with indexes x and y 
            (projected coordinates) and aggregate metric column
        metric: target column for aggregate. Default: postcount.
        responsive: Should be True for interactive HTML output.
        hover_items: additional items to show on hover
        dim_nonsignificant: transparent bins if significant column == False
        scheme: The classification scheme to use. Default "HeadTailBreaks".
        cmap: The colormap to use. Default "OrRd".
    """
    # work on a shallow copy,
    # to not modify original dataframe
    poly_gdf_plot = poly_gdf.copy()
    kwargs = {
        "poly_gdf":poly_gdf_plot,
        "metric":metric,
        "hover_items":hover_items,
        "mask_nonsignificant":mask_nonsignificant,
        "scheme":scheme, "cmap_name":cmap_name,
        "cosine_country":cosine_country
    }
    # get gv.Image layer, cmap, and label dict (legend)
    gv_poly, cmap, label_dict = get_classify_poly(**kwargs)
    # apply display opts to gv.Image layer
    gv_poly = apply_layer_opts_poly(
        gv_poly=gv_poly, cmap=cmap, label_dict=label_dict,
        responsive=responsive, hover_items=hover_items)
    return gv_poly
```

Override custom hover tooltip, to render list of tfidf as custom html.

In [23]:

```
def get_custom_tooltips(items: Dict[str, str]) -> str:
    """Compile HoverTool tooltip formatting with items to show on hover
    including showing a thumbail image from a url"""
    tdelim_format = [
        'cosine']
    # format html
    tooltips = "".join(
        f'<div><span style="font-size: 12px;">'
        f'<span style="color: #82C3EA;">{k}:</span> '
        f'@{v}'
        f'</span></div>' for k, v in items.items() if v not in ["tfidf"])
    if 'tfidf' in items.values():
        tooltips += f'''
            <span style="color: #82C3EA;">Top 20 terms (TFIDF):</span> 
            <div style="width:100px">@tfidf</div>'''
    return tooltips
```

In [24]:

```
def apply_layer_opts_poly(
    gv_poly: gv.Polygons, cmap: colors.ListedColormap,
    label_dict: Dict[str, str], responsive: bool = None,
    hover_items: Dict[str, str] = None) -> gv.Image:
    """Apply geoviews image layer opts

    Args:
        img_grid: A classified gv.Image layer
        responsive: Should be True for interactive HTML output.
        hover_items: additional items to show on hover
        cmap: A matplotlib colormap to colorize values and show as legend.
    """
    color_levels = len(cmap.colors)
    # define additional plotting parameters
    # width of static jupyter map,
    # 360° == 1200px
    width = 1200 
    # height of static jupyter map,
    # 360°/2 == 180° == 600px
    height = int(width/2) 
    aspect = None
    # if stored as html,
    # override values
    if responsive:
        width = None
        height = None
    # define width and height as optional parameters
    # only used when plotting inside jupyter
    optional_kwargs = dict(width=width, height=height)
    # compile only values that are not None into kwargs-dict
    # by using dict-comprehension
    optional_kwargs_unpack = {
        k: v for k, v in optional_kwargs.items() if v is not None}
    # prepare custom HoverTool
    tooltips = get_custom_tooltips(
        hover_items)
    hover = HoverTool(tooltips=tooltips)
    # get tick positions from label dict keys
    ticks = [key for key in sorted(label_dict)]
    # create image layer
    gv_poly = gv_poly.opts(
            color_levels=color_levels,
            cmap=cmap,
            colorbar=True,
            line_color='grey',
            line_width=0.3,
            clipping_colors={'NaN': 'transparent'},
            colorbar_opts={
                # 'formatter': formatter,
                'major_label_text_align':'left',
                'major_label_overrides': label_dict,
                'ticker': FixedTicker(
                    ticks=ticks),
                },
            tools=[hover],
            # optional unpack of width and height
            **optional_kwargs_unpack
        )
    return gv_poly
```

In [25]:

```
def plot_interactive_cosine(
    cosine_country: str, title: str,
    cosine_source: Path = TERMS_FLICKR_COSINE,
    metric: str = "chi_value",
    mask_nonsignificant: bool = False,
    scheme: str = "HeadTailBreaks",
    cmap: str = "OrRd",
    store_html: str = None,
    plot: Optional[bool] = True,
    output: Optional[str] = OUTPUT,) -> gv.Overlay:
    """Plot interactive map with holoviews/geoviews renderer

    Args:
        poly_gdf: A geopandas geodataframe with polygons 
            (projected coordinates) and aggregate metric column
        metric: target column for aggregate. Default: postcount.
        store_html: Provide a name to store figure as interactive HTML.
        title: Title of the map
        hover_items: additional items to show on hover
        mask_nonsignificant: transparent bins if significant column == False
        scheme: The classification scheme to use. Default "HeadTailBreaks".
        cmap: The colormap to use. Default "OrRd".
        plot: Prepare gv-layers to be plotted in notebook.
    """
    hover_items = {
        'Country':'ADMIN', 
        'Country Code (su_a3)':'su_a3', 
        'Cosine Similarity':'cosine',
        'Top 20 terms (TFIDF)':'tfidf', }
    df_cos = load_cosine_df()
    world = load_combine(cosine_country, df_cos)
    # store su_a3 codes as normal column, too
    # so the code can be shown on hover
    world['su_a3'] = world.index
    # check if all additional items are available
    for key, item in list(hover_items.items()):
        if item not in world.columns:
            hover_items.pop(key)
    # poly layer opts
    # global plotting options for values layer
    layer_opts = {
        "metric":metric,
        "responsive":False,
        "mask_nonsignificant":mask_nonsignificant,
        "scheme":scheme,
        "hover_items":hover_items,
        "cmap_name":cmap,
        "cosine_country":cosine_country
    }
    # global plotting options for all layers (gv.Overlay)
    gv_opts = {
        "bgcolor":None,
        # "global_extent":True,
        "projection":crs.Mollweide(),
        "responsive":False,
        "data_aspect":1, # maintain fixed aspect ratio during responsive resize
        "hooks":[set_active_tool],
        "title":title
    }
    # Create gv layers
    sel_poly_layer = gv.Polygons(
        world.loc[cosine_country].geometry,
        crs=crs.Mollweide()).opts(
            line_color='white',
            line_width=1,
            fill_color='#420603')
    # selected country centroid
    centroid = world.loc[cosine_country].geometry.centroid
    centroid_proj = PROJ_TRANSFORMER_BACK.transform(
        centroid.x, centroid.y)
    if plot:
        # get classified polygon gv layer
        poly_layer = compile_poly_layer(
            poly_gdf=world, **layer_opts)
        gv_layers = gv.Overlay(
            [poly_layer, sel_poly_layer])
    if store_html:
        # get as responsive
        layer_opts["responsive"] = True
        poly_layer = compile_poly_layer(
            poly_gdf=world, **layer_opts)
        sel_poly_layer.opts(responsive=True)
        responsive_gv_layers = gv.Overlay(
            [poly_layer, sel_poly_layer])
        gv_opts["responsive"] = True
        export_layers = responsive_gv_layers.opts(**gv_opts)
        hv.save(
            export_layers,
            output / f"html" / f'{store_html}.html', backend='bokeh')
        if WEB_DRIVER:
            # store also as svg
            p =  hv.render(export_layers, backend='bokeh')
            p.output_backend = "svg"
            export_svgs(
                p, filename=output / f"svg{km_size_str}" / f'{store_html}.svg',
                webdriver=WEB_DRIVER)
    if not plot:
        return
    gv_opts["responsive"] = False
    return gv_layers.opts(**gv_opts)
```

The methods defined in `01_grid_agg.ipynb`,  
for rounding label float numbers, are not suitable  
for the small cosine similarity values.

Below, new methods are defined (with minimum of 2  
decimals rounding precision). These override the  
previously defined methods.

In [26]:

```
import _01_grid_agg
def _rnd_f_cosine(f: float, dec: int = None) -> str:
    if dec is None:
        dec = 2
    return f'{f:,.{dec}f}'

def min_decimals_cosine(num1: float, num2: float) -> int:
    """Return number of minimum required decimals"""
    if _rnd_f_cosine(num1) != _rnd_f_cosine(num2):
        return 2
    for i in range(3, 5):
        if _rnd_f_cosine(num1, i) != _rnd_f_cosine(num2, i):
            return i
    return 5

_01_grid_agg = sys.modules["_01_grid_agg"]
_01_grid_agg.min_decimals = min_decimals_cosine
```

Define country to show cosine similarities for and the output filename:

In [27]:

```
cosine_country = "ZMB"
filename = f"sunset_cosine_flickr_{cosine_country}"
cosine_source = TERMS_FLICKR_COSINE
```

In [28]:

```
gv_plot = plot_interactive_cosine(
    cosine_source=cosine_source, cosine_country=cosine_country,
    title=f'Cosine similarity: Flickr "Sunset" context terms similarity for country {cosine_country}',
    metric="cosine", scheme="HeadTailBreaks", cmap="OrRd", store_html=filename)
gv_plot
```

Out[28]:

For comparison, have a look at the similarity score for Indonesia (IDN)

In [29]:

```
cosine_country = "IDN"
filename = f"sunset_cosine_flickr_{cosine_country}"
cosine_source = TERMS_FLICKR_COSINE
```

In [30]:

```
gv_plot = plot_interactive_cosine(
    cosine_source=cosine_source, cosine_country=cosine_country,
    title=f'Cosine similarity: Flickr "Sunset" context terms similarity for country {cosine_country}',
    metric="cosine", scheme="HeadTailBreaks", cmap="OrRd", store_html=filename)
gv_plot
```

Out[30]:

**ToDo:**

For now, the map must be re-generated for visualizing cosine-similarities for each country. A possible future extension could use a Panel Dashboard to allow interactive selection.

# Create notebook HTML¶

In [34]:

```
!jupyter nbconvert --to html_toc \
    --output-dir=../out/html ./06_semantics.ipynb \
    --template=../nbconvert.tpl \
    --ExtractOutputPreprocessor.enabled=False >&- 2>&- # create single output file
```

Copy single HTML file to resource folder

In [35]:

```
!cp ../out/html/06_semantics.html ../resources/html/
```

In [ ]:

```

```
