# Supplementary material for: From sunrise to sunset: Exploring landscape preference through global reactions to ephemeral events captured in georeferenced social media
Source: PLoS One. 2023 Feb 22;18(2):e0280423. doi: 10.1371/journal.pone.0280423 (PMC9946259; doi:10.1371/journal.pone.0280423)
Supplement: S7 File — (HTML) [file pone.0280423.s007.html]

07\_time


# Temporal overview sunset/sunrise¶

*Alexander Dunkel, TU Dresden, Institute of Cartography; Maximilian Hartmann, Universität Zürich (UZH), Geocomputation*

---

•••

Out[1]:

Last updated: Jan-17-2023, Carto-Lab Docker Version 0.9.0

A brief look at the temporal distribution of the collected sunset/sunrise data.

# Preparations¶

## Load dependencies¶

This time, we use the python\_hll package to calculate hll set cardinalities.  
`python_hll` is significantly slower than the native Postgres HLL implementation.  
But there are only a few temporal HLL sets to calculate (year and months aggregates).

In [2]:

```
import sys
import pandas as pd
from pathlib import Path
from python_hll.hll import HLL
from python_hll.util import NumberUtil
module_path = str(Path.cwd().parents[0] / "py")
if module_path not in sys.path:
    sys.path.append(module_path)
from modules import tools
from _03_chimaps import OUTPUT
```

```
Chromedriver loaded. Svg output enabled.
```

## Load HLL aggregate data¶

Data is stored as aggregate HLL data (postcount) for each month.

In [3]:

```
root = Path.cwd().parents[1] / "00_hll_data"
SUNRISE_INSTAGRAM = root / "instagram-sunrise-months.csv"
SUNSET_INSTAGRAM = root / "instagram-sunset-months.csv"
SUNRISE_FLICKR = root / "flickr-sunrise-months.csv"
SUNSET_FLICKR = root / "flickr-sunset-months.csv"
```

Some statistics for these files:

In [4]:

```
%%time
data_files = {
    "SUNRISE_INSTAGRAM":SUNRISE_INSTAGRAM,
    "SUNSET_INSTAGRAM":SUNSET_INSTAGRAM,
    "SUNRISE_FLICKR":SUNRISE_FLICKR,
    "SUNSET_FLICKR":SUNSET_FLICKR,
    }
tools.display_file_stats(data_files)
```

| name | SUNRISE\_INSTAGRAM | SUNSET\_INSTAGRAM | SUNRISE\_FLICKR | SUNSET\_FLICKR |
| --- | --- | --- | --- | --- |
| size | 20.14 KB | 19.12 KB | 359.84 KB | 359.87 KB |
| records | 36 | 27 | 145 | 145 |

```
CPU times: user 31.5 ms, sys: 0 ns, total: 31.5 ms
Wall time: 88.7 ms
```

Preview CSV:

In [5]:

```
df = pd.read_csv(SUNSET_FLICKR, nrows=10)
```

In [6]:

```
display(df)
```

|  | year | month | post\_hll |
| --- | --- | --- | --- |
| 0 | 2007 | 1 | \x148b7f10c4218c43188e42144110c8810425298a228c... |
| 1 | 2007 | 2 | \x148b7f1946118822390412886118484294a530442188... |
| 2 | 2007 | 3 | \x148b7f188621904221084110a111c261908121025208... |
| 3 | 2007 | 4 | \x148b7f108a41046629ce520882308a309042194e23a5... |
| 4 | 2007 | 5 | \x148b7f11443108441106329845188424186220842298... |
| 5 | 2007 | 6 | \x148b7f294831908428ca2214853088518cc13050721c... |
| 6 | 2007 | 7 | \x148b7f10c83290e4210a510c842110518ca31144320c... |
| 7 | 2007 | 8 | \x148b7f2086610ca431043208831084620847298a318c... |
| 8 | 2007 | 9 | \x148b7f310a420cc218cc418c8629ca528ce73146519c... |
| 9 | 2007 | 10 | \x148b7f210c6109022148310c662042310864390a4114... |

# Calculate HLL Cardinality per month¶

**Prepare functions**

These functions were first used in the YFCC HLL Workshop.

In [7]:

```
def hll_from_byte(hll_set: str):
    """Return HLL set from binary representation"""
    hex_string = hll_set[2:]
    return HLL.from_bytes(
        NumberUtil.from_hex(
            hex_string, 0, len(hex_string)))

def cardinality_from_hll(hll_set):
    """Turn binary hll into HLL set and return cardinality"""
    hll = hll_from_byte(hll_set)
    return hll.cardinality() - 1
```

Define additional functions for reading and formatting CSV as `pd.DataFrame`

In [8]:

```
def read_csv_datetime(csv: Path) -> pd.DataFrame:
    """Read CSV with parsing datetime index (months)
    
        First CSV column: Year
        Second CSV column: Month
    """
    date_cols = ["year", "month"]
    df = pd.read_csv(
        csv, index_col='datetime', 
        parse_dates={'datetime':date_cols},
        date_parser=lambda x: pd.datetime.strptime(x, '%Y %m'),
        keep_date_col='False')
    df.drop(columns=date_cols, inplace=True)
    return df
    
def append_cardinality_df(df: pd.DataFrame, hll_col: str = "post_hll"):
    """Calculate cardinality from HLL and append to extra column in df"""
    df['postcount_est'] = df.apply(
        lambda x: cardinality_from_hll(
           x[hll_col]),
        axis=1)
    df.drop(columns=["post_hll"], inplace=True)
    return df

def filter_fill_time(
        df: pd.DataFrame, min_year: int, 
        max_year: int, val_col: str = "postcount_est"):
    """Filter time values between min - max year and fill missing values"""
    min_date = pd.Timestamp(f'{min_year}-01-01')
    max_date = pd.Timestamp(f'{max_year}-01-01')
    # clip by start and end date
    if not min_date in df.index:
        df.loc[min_date, val_col] = 0
    if not max_date in df.index:
        df.loc[min_date, val_col] = 0
    df.sort_index(inplace=True)
    # mask min and max time
    time_mask = ((df.index >= min_date) & (df.index <= max_date))
    # fill missing months with 0
    # this will also set the day to max of month
    series = df.loc[time_mask][val_col].resample('M').sum().fillna(0)
    return series.to_frame()
```

Apply functions to all data sets.

- Read from CSV
- calculate cardinality
- merge year and month to single column
- filter 2007 - 2018 range, fill missing values

In [9]:

```
%%time
import warnings; warnings.simplefilter('ignore')
dfs = {}
for key, data in data_files.items():
    df = read_csv_datetime(data)
    df = append_cardinality_df(df)
    df = filter_fill_time(df, 2007, 2018)
    dfs[key] = df
```

```
CPU times: user 19.6 s, sys: 12.8 ms, total: 19.6 s
Wall time: 19.7 s
```

**RuntimeWarning?** 

- python-hll library is in a very early stage of development
- it is not fully compatible with the citus hll implementation in postgres
- The shown RuntimeWarning (Overflow) is one of the issues that need to be resolved in the future
- If you run this notebook locally, it is recommended to use pg-hll-empty for
  any hll calculations, as is shown (e.g.) in the original YFCC100M notebooks.
- There is no significant negative impact on accuracy for this application case.

In [10]:

```
dfs["SUNRISE_FLICKR"].head(5)
```

Out[10]:

|  | postcount\_est |
| --- | --- |
| datetime |  |
| 2007-01-31 | 2787 |
| 2007-02-28 | 2300 |
| 2007-03-31 | 3386 |
| 2007-04-30 | 2630 |
| 2007-05-31 | 2407 |

# Visualize Cardinality¶

Define plot function.

In [11]:

```
import matplotlib.pyplot as plt
import matplotlib.ticker as mticker
import seaborn as sns
from matplotlib.axes import Axes 
from matplotlib import cm
from typing import Tuple

def bar_plot_time(
        df: pd.DataFrame, ax: Axes, color: str, label: str,
        val_col: str = "postcount_est") -> Axes:
    """Matplotlib Barplot with time axis formatting"""
    ax = df.set_index(
        df.index.map(lambda s: s.strftime('%Y'))).plot.bar(
            ax=ax, y="postcount_est", color=color, width=1.0,
            label=label, edgecolor="white", linewidth=0.5, alpha=0.8)
    return ax

def plot_time(dfs: Tuple[pd.DataFrame, pd.DataFrame], title, filename = None, output = OUTPUT):
    """Create dataframe(s) time plot"""
    fig, ax = plt.subplots()
    fig.set_size_inches(15.7, 4.27)
    # get color
    colors = sns.color_palette("vlag", as_cmap=True, n_colors=2)
    # sunset
    ax = bar_plot_time(
        df=dfs[0], ax=ax, color=colors([1.0]), label="Sunset")
    # sunrise
    ax = bar_plot_time(
        df=dfs[1], ax=ax, color=colors([0.0]), label="Sunrise")
    # x axis ticker formatting
    tick_loc = mticker.MultipleLocator(12)
    ax.xaxis.set_major_locator(tick_loc)
    ax.tick_params(axis='x', rotation=45)
    ax.ticklabel_format(axis='y', style='plain')
    ax.set(xlabel="Month", ylabel="Post Count (estimate)")
    ax.spines["left"].set_linewidth(0.25)
    ax.spines["bottom"].set_linewidth(0.25)
    ax.spines["top"].set_linewidth(0)
    ax.spines["right"].set_linewidth(0)
    ax.yaxis.set_tick_params(width=0.5)
    # add legend
    h, l = ax.get_legend_handles_labels()
    ax.legend(h, l, frameon=False, loc='center left', bbox_to_anchor=(1, 0.5))
    ax.set_title(title)
    # store figure to file
    if filename:
        fig.savefig(
            output / "figures" / f"{filename}.png", dpi=300, format='PNG',
            bbox_inches='tight', pad_inches=1, facecolor="white")
        # also save as svg
        fig.savefig(
            output / "svg" / f"{filename}.svg", format='svg',
            bbox_inches='tight', pad_inches=1, facecolor="white")
```

Plot sunset and sunrise for both Instagram and Flickr collected data.

In [12]:

```
plot_time(
    dfs = (dfs["SUNSET_INSTAGRAM"], dfs["SUNRISE_INSTAGRAM"]),
    title='Instagram sunset and sunrise reactions', 
    filename="temporal_analysis_instagram")
```

In [13]:

```
plot_time(
    dfs = (dfs["SUNSET_FLICKR"], dfs["SUNRISE_FLICKR"]),
    title='Flickr sunset and sunrise reactions', 
    filename="temporal_analysis_flickr")
```

# Create notebook HTML¶

In [19]:

```
!jupyter nbconvert --to html_toc \
    --output-dir=../out/html ./07_time.ipynb \
    --template=../nbconvert.tpl \
    --ExtractOutputPreprocessor.enabled=False >&- 2>&- # create single output file
```

Copy single HTML file to resource folder

In [20]:

```
!cp ../out/html/07_time.html ../resources/html/
```

In [ ]:

```

```
