# Supplementary material for: From sunrise to sunset: Exploring landscape preference through global reactions to ephemeral events captured in georeferenced social media
Source: PLoS One. 2023 Feb 22;18(2):e0280423. doi: 10.1371/journal.pone.0280423 (PMC9946259; doi:10.1371/journal.pone.0280423)
Supplement: S8 File — (HTML) [file pone.0280423.s008.html]

08\_relationships


# Relationships ¶

*Alexander Dunkel, TU Dresden, Institute of Cartography; Maximilian Hartmann, Universität Zürich (UZH), Geocomputation; Ross Purves, Universität Zürich (UZH), Geocomputation*

---

•••

Out[1]:

Last updated: Jan-17-2023, Carto-Lab Docker Version 0.9.0

# Introduction¶

In this notebook, we study different relationships between different sets of information:

- bias for sunset and sunrise per grid bin
- bias for instagram and flickr
- bias for different metrics (userdays, usercount, postcount)

See the introduction to Correlation With Python.

**TODO:** Notebook cleanup

# Preparations¶

## Load dependencies¶

Import code from other jupyter notebooks, synced to \*.py with jupytext:

In [2]:

```
import sys
from pathlib import Path
module_path = str(Path.cwd().parents[0] / "py")
if module_path not in sys.path:
    sys.path.append(module_path)
# import all previous chained notebooks
from _04_combine import *
```

```
Chromedriver loaded. Svg output enabled.
```

Activate autoreload of changed python files:

In [3]:

```
%load_ext autoreload
%autoreload 2
```

**Load additional dependencies**

In [4]:

```
import seaborn as sns
import matplotlib.pyplot as plt
from scipy import stats
```

## Parameters¶

- Define which metric to use for relationship study.
- define if relationships are only studied looking at significant chi-square value grid bins

In [5]:

```
METRIC = 'usercount'
ONLY_SIGNIFICANT = False
```

The column for this metric ends with 'est', due to HLL estimates:

In [6]:

```
METRIC_COL = f"{METRIC}_est"
```

# Load data¶

Load grid data using the `chimaps_fromcsv()` method. This data includes the absolute measurements.

In [7]:

```
grid = chimaps_fromcsv(
    plot=False, chi_column=METRIC_COL, normalize=False)
```

Drop cols not needed (chi data, summary data for merged chi values sunset and sunrise):

In [8]:

```
drop_cols = [
    'chi_value_sunset', 'chi_value_sunrise', 'chi_value', 'significant', 
    'underrepresented', 'significant', 'significant_sunset', 'significant_sunrise',
    'usercount_est_expected', 'userdays_est_expected', 'postcount_est_expected']
grid.drop(columns=drop_cols, inplace=True, errors='ignore')
```

In [9]:

```
grid.columns
```

Out[9]:

```
Index(['geometry', 'postcount_est_sunset', 'usercount_est_sunset',
       'userdays_est_sunset', 'usercount_est_sunrise', 'postcount_est_sunrise',
       'userdays_est_sunrise'],
      dtype='object')
```

# Prepare Data¶

## Metric Comparison: Usercount, Userdays, Postcount¶

Single very active users may have significant influence on userdays and postcount metrics.

One question here is, how large is the influence of very active, single users on aggregate metrics per bin?

In order to highlight this influence in graphics, calculate the ratio between usercount and postcount/userdays and classify top10 ratios.

1. Divide the number of posts per user (`postcount / usercount`) per grid bin
2. Classify ratios (high/low)
3. Colorize relationship plots with classes

In [10]:

```
grid[grid['postcount_est_sunset'] >= 1000].drop(columns=['geometry']).head()
```

Out[10]:

|  |  | postcount\_est\_sunset | usercount\_est\_sunset | userdays\_est\_sunset | usercount\_est\_sunrise | postcount\_est\_sunrise | userdays\_est\_sunrise |
| --- | --- | --- | --- | --- | --- | --- | --- |
| xbin | ybin |  |  |  |  |  |  |
| -15340096 | 2779952 | 2946 | 657 | 1036 | 134 | 363 | 367 |
| -15240096 | 2679952 | 11300 | 2115 | 4259 | 369 | 1242 | 1175 |
| -15140096 | 2679952 | 2946 | 728 | 1184 | 369 | 1758 | 1782 |
|  | 2579952 | 8594 | 1811 | 3696 | 254 | 1034 | 996 |
|  | 2479952 | 2291 | 672 | 1194 | 40 | 97 | 95 |

In [11]:

```
SUR_NAME_LIST = [
    '_est_sunset',
    '_est_sunrise']
```

# Create Plots¶

## Plot relationship: Chi square sunset (x) and sunrise (y) per grid bin¶

Relationship between chi square values for sunset and sunrise for different grid bins.

In [12]:

```
sns.set_theme(style="whitegrid")
```

Prepare annotation:

- Add plot annotation for r² and p, covariance.
- Adapted from source and r²

In [13]:

```
from matplotlib.lines import Line2D

def annotate(
    data, x_col, y_col, ranked=False, **kws):
    """Add r², p and covariance to plot, format legend"""
    x = data[x_col]
    y = data[y_col]
    nas = np.logical_or(x.isna(), y.isna())
    cov = None
    if not ranked:
        r, p = stats.pearsonr(
            x[~nas],
            y[~nas])
        # covariance
        cov = np.cov(data[x_col], data[y_col])[0][1]
    else:
        r, p = stats.spearmanr(
            x[~nas],
            y[~nas])     
    # r²
    correlation_matrix = np.corrcoef(data[x_col], data[y_col])
    correlation_xy = correlation_matrix[0, 1]
    r_squared = correlation_xy**2
    # update legend
    ax = plt.gca()
    handles, labels = ax.get_legend_handles_labels()
    label = ""
    if r:
        label = f"{label} \nStatistics:\nr={r:.2f},"
    if p:
        label = f"{label} \np={p:.2g},"
    if cov:
        label = f"{label} \ncov={cov:.2f}"
    if r_squared:
        label = f"{label} \nr²={r_squared:.2f}"
    patch = Line2D(
        [0], [0],
        color=None,
        linestyle="None",
        label=label)
    handles.append(patch) 
    plt.legend(
        handles=handles, loc='upper left',
        bbox_to_anchor=(1.04,1), frameon=False)
```

Plot

In [14]:

```
def relationship_plot(
    data: gp.GeoDataFrame,
    title: str,
    x_col: str = f'{METRIC_COL}_sunset_cbrt',
    y_col: str = f'{METRIC_COL}_sunrise_cbrt',
    x_label: str = f'{METRIC} Sunset (cube root)',
    y_label: str = f'{METRIC} Sunrise (cube root)',
    figsize: Tuple[int, int] = (7, 7),
    plot_context: str = "100 km grid bin"):
    """Create relationship plot"""
    fig, ax = plt.subplots(figsize=figsize)
    fig.suptitle(
        title,
        fontsize=12, y=0)
    scatterplot_kwarg = {
        "ax":ax,
        "edgecolors":"white",
        "linewidth":1,
        "x":x_col,
        "y":y_col,
    }

    g = sns.scatterplot(
        data=data,
        color='grey', **scatterplot_kwarg, label=plot_context)
    # get topic for y and x axis (e.g. sunset, surnise)
    y_topic = y_label.split('(')[0].split()[-1]
    x_topic = x_label.split('(')[0].split()[-1]
    # add numbers to plot
    ranked = False
    if x_col.endswith('_rank'):
        ranked=True
    annotate(
        data=data,
        x_col=x_col,
        y_col=y_col,
        ranked=ranked)
    ax.set_xlabel(x_label)
    ax.set_ylabel(y_label)
```

## Relationship metrics: Covariance, Correlation Coefficients¶

### Covariance¶

Covariance can be calculated with weights or without. Since we are using absolute userday frequencies, we do not use weights.

link
docs

> cov(a,a) cov(a,b)
>
> cov(a,b) cov(b,b)

**Without weights:**

In [15]:

```
covariance = np.cov(
    grid[f'{METRIC_COL}_sunset'], grid[f'{METRIC_COL}_sunrise'])
print(covariance)
```

```
[[10180.45109038  2750.78933315]
 [ 2750.78933315   888.52663159]]
```

Output as a single number

In [16]:

```
print(covariance[0][1])
```

```
2750.789333146508
```

As expected, sunset and sunrise reactions have a positive relationship: In other words, typically, where people react to sunset, reactions to sunrise are also found, and vice versa.

> A problem with covariance as a statistical tool alone is that it is challenging to interpret. This leads us to the Pearson’s correlation coefficient next.

### Correlation Coefficients¶

- Unlike covariance, Correlation Coefficients do not offer the ability to include z-values (weights).
- only focus: correlation between absolute values

(Pearson correlation coefficient)

**Userdays:**

In [17]:

```
x = grid[f'{METRIC_COL}_sunset']
y = grid[f'{METRIC_COL}_sunrise']
r = np.corrcoef(x, y)
r
```

Out[17]:

```
array([[1.        , 0.91461558],
       [0.91461558, 1.        ]])
```

Same as:

In [18]:

```
from scipy.stats import pearsonr
corr, _ = pearsonr(x, y)
print('Pearsons correlation: %.3f' % corr)
```

```
Pearsons correlation: 0.915
```

> The coefficient returns a value between -1 and 1 that represents the limits of correlation from a full negative correlation to a full positive correlation. A value of 0 means no correlation. The value must be interpreted, where often a value below -0.5 or above 0.5 indicates a notable correlation, and values below those values suggests a less notable correlation.

Interpretation for sunset/sunrise: There's a notable relationship between the two events in that sunrise reactions tend to increase when sunset reactions increase. In other words, locations that people prefer to view sunsets also tend to feature a suitability for sunrise, as already observed with the covariance test above.

**User Count:**

Compare to user counts

In [19]:

```
x = grid[f"usercount_est_sunset"]
y = grid[f"usercount_est_sunrise"]
r = np.corrcoef(x, y)
display(pd.DataFrame(r))
```

|  | 0 | 1 |
| --- | --- | --- |
| 0 | 1.000000 | 0.914616 |
| 1 | 0.914616 | 1.000000 |

The correlation is quite a bit stronger for usercounts, instead of userdays, indicating a higher variability of the userday measurement.

**Post Count:**

Compare to user counts

In [20]:

```
x = grid[f"postcount_est_sunset"]
y = grid[f"postcount_est_sunrise"]
r = np.corrcoef(x, y)
display(pd.DataFrame(r))
```

|  | 0 | 1 |
| --- | --- | --- |
| 0 | 1.000000 | 0.794686 |
| 1 | 0.794686 | 1.000000 |

Surprisingly, post count correlation is higher than userday correlation, which would mean that the userday measurement has the highest variability of all measurements.

**Spearman rank-order correlation:**

Since we use ranked data, Spearman rank-order correlation test is more approiate here.

In [21]:

```
x = grid[f'{METRIC_COL}_sunset']
y = grid[f'{METRIC_COL}_sunrise']
corr, pval = stats.spearmanr(x, y)
print(f'Spearman correlation: {corr:.3f} with p={pval:.3f}')
```

```
Spearman correlation: 0.783 with p=0.000
```

**Ranking Correlation**

In order to focus on the relationship, not relative distribution of values, it is possible to compare ranks for countries.

In [22]:

```
def rank_series(series: pd.Series) -> pd.Series:
    """Create ranking for series (1, 2..., x)
    and return as series of numbers (int).
    """
    return series[series > 0].rank()

def rank_cols(grid: pd.DataFrame, topic1="sunset", topic2="sunrise", metric_col = METRIC_COL):
    """Create ranks for two columns in grid, store as new cols"""
    grid[f'{metric_col}_{topic1}_rank'] =  rank_series(grid[f'{metric_col}_{topic1}'])
    grid[f'{metric_col}_{topic2}_rank'] =  rank_series(grid[f'{metric_col}_{topic2}'])
```

## Relationship between values from Sunset and Sunrise (100km grid)¶

We'll use ranked comparison for the relationship plots below.

In [23]:

```
grid_sunrise = grid_agg_fromcsv(OUTPUT / "csv" / "flickr_sunrise_est.csv")
grid_sunset = grid_agg_fromcsv(OUTPUT / "csv" / "flickr_sunset_est.csv")
```

In [24]:

```
grid_sunrise.head()
```

Out[24]:

|  |  | geometry | postcount\_est | usercount\_est | userdays\_est |
| --- | --- | --- | --- | --- | --- |
| xbin | ybin |  |  |  |  |
| -18040096 | 8979952 | POLYGON ((-18040096.000 8979952.000, -17940096... | 0 | 0 | 0 |
|  | 8879952 | POLYGON ((-18040096.000 8879952.000, -17940096... | 0 | 0 | 0 |
|  | 8779952 | POLYGON ((-18040096.000 8779952.000, -17940096... | 0 | 0 | 0 |
|  | 8679952 | POLYGON ((-18040096.000 8679952.000, -17940096... | 0 | 0 | 0 |
|  | 8579952 | POLYGON ((-18040096.000 8579952.000, -17940096... | 0 | 0 | 0 |

Calculate ranks from absolute numbers.

In [25]:

```
def rank_cols_dfs(df1, df2, topic1="sunrise", topic2="sunset", metric=METRIC):
    """Rank columns of df_sunset, df_sunrise"""
    metric_col = metric
    if metric != "chi_value":
        metric_col = f"{metric}_est"
    df1[f'{metric}_{topic1}_rank'] = rank_series(
       df1[metric_col])
    df2[f'{metric}_{topic2}_rank'] = rank_series(
       df2[metric_col])
```

In [26]:

```
rank_cols_dfs(grid_sunrise, grid_sunset, metric=METRIC)
```

Merge

In [27]:

```
def merge_df_topics(df1, df2, topic1="sunrise", topic2="sunset", metric=METRIC, ranked: bool = True) -> pd.DataFrame:
    """Merge sunset and sunrise/ flickr and instagram values"""
    _rank = ""
    if ranked:
        _rank = "_rank"
    df = df1[[f'{metric}_{topic1}{_rank}']].merge(
        df2[[f'{metric}_{topic2}{_rank}']],
        left_index=True, right_index=True)
    return df
```

In [28]:

```
grid = merge_df_topics(grid_sunrise, grid_sunset, metric=METRIC)
```

In [29]:

```
title = (
    f'Relationship between {METRIC} per grid cell (ranked) \n'
    'for Sunset and Sunrise from Flickr. ')
x_col = f'{METRIC}_sunset_rank'
y_col = f'{METRIC}_sunrise_rank'
relationship_plot(
    data=grid, title=title, x_col=x_col, y_col=y_col,
    x_label=f'{METRIC.title()} Sunset (ranked)', y_label=f'{METRIC.title()} Sunrise (ranked)')
```

## Relationship between values from Flickr and Instagram¶

### Load Data: Combine Instagram and Fickr data¶

In [30]:

```
grid_flickr = chimaps_fromcsv(
    plot=False, chi_column=METRIC_COL, normalize=False)
```

In [31]:

```
instagram_args = {
    "csv_observed_plus":"instagram_sunset_est.csv",
    "csv_observed_minus":"instagram_sunrise_est.csv",
    "csv_expected":"instagram_random_est.csv"}
grid_instagram = chimaps_fromcsv(
    plot=False, chi_column=METRIC_COL, normalize=False, **instagram_args)
```

Remove cols not needed for the relationship plots.

In [32]:

```
grid_flickr.drop(columns=drop_cols, inplace=True, errors='ignore')
grid_instagram.drop(columns=drop_cols, inplace=True, errors='ignore')
```

In [33]:

```
COLMAP_FLICKR = {
    f'{METRIC_COL}_sunrise':f'{METRIC_COL}_sunrise_flickr',
    f'{METRIC_COL}_sunset':f'{METRIC_COL}_sunset_flickr'}
COLMAP_INSTAGRAM = {
    f'{METRIC_COL}_sunrise':f'{METRIC_COL}_sunrise_instagram',
    f'{METRIC_COL}_sunset':f'{METRIC_COL}_sunset_instagram'}
```

In [34]:

```
grid_rename_cols(grid_flickr, COLMAP_FLICKR)
grid_rename_cols(grid_instagram, COLMAP_INSTAGRAM)
```

In [35]:

```
grid_flickr.drop(columns=['geometry']).head()
```

Out[35]:

|  |  | postcount\_est\_sunset | usercount\_est\_sunset\_flickr | userdays\_est\_sunset | usercount\_est\_sunrise\_flickr | postcount\_est\_sunrise | userdays\_est\_sunrise |
| --- | --- | --- | --- | --- | --- | --- | --- |
| xbin | ybin |  |  |  |  |  |  |
| -18040096 | 8979952 | 0 | 0 | 0 | 0 | 0 | 0 |
|  | 8879952 | 0 | 0 | 0 | 0 | 0 | 0 |
|  | 8779952 | 0 | 0 | 0 | 0 | 0 | 0 |
|  | 8679952 | 0 | 0 | 0 | 0 | 0 | 0 |
|  | 8579952 | 0 | 0 | 0 | 0 | 0 | 0 |

Merge both grids and rename metric columns:

In [36]:

```
merge_cols = [
    f'{METRIC_COL}_sunrise_instagram',
    f'{METRIC_COL}_sunset_instagram']
grid = merge_df(grid_flickr, grid_instagram, merge_cols)
```

In [37]:

```
preview_mask = grid[f'{METRIC_COL}_sunrise_instagram']>1000
grid[preview_mask].drop(columns=['geometry']).head()
```

Out[37]:

|  |  | postcount\_est\_sunset | usercount\_est\_sunset\_flickr | userdays\_est\_sunset | usercount\_est\_sunrise\_flickr | postcount\_est\_sunrise | userdays\_est\_sunrise | usercount\_est\_sunrise\_instagram | usercount\_est\_sunset\_instagram |
| --- | --- | --- | --- | --- | --- | --- | --- | --- | --- |
| xbin | ybin |  |  |  |  |  |  |  |  |
| -15240096 | 2679952 | 11300 | 2115 | 4259 | 369 | 1242 | 1175 | 2663 | 16941 |
| -15140096 | 2679952 | 2946 | 728 | 1184 | 369 | 1758 | 1782 | 3345 | 9862 |
|  | 2579952 | 8594 | 1811 | 3696 | 254 | 1034 | 996 | 1039 | 8642 |
| -15040096 | 2579952 | 1637 | 474 | 747 | 642 | 2933 | 2855 | 1721 | 2531 |
| -10640096 | 4579952 | 45923 | 7592 | 20481 | 2030 | 9931 | 9037 | 7613 | 33931 |

Calculate rank series for userdays:

In [38]:

```
grid[f'{METRIC_COL}_sunset_flickr_rank'] = rank_series(grid[f'{METRIC_COL}_sunset_flickr'])
grid[f'{METRIC_COL}_sunset_instagram_rank'] = rank_series(grid[f'{METRIC_COL}_sunset_instagram'])
grid[f'{METRIC_COL}_sunrise_flickr_rank'] = rank_series(grid[f'{METRIC_COL}_sunrise_flickr'])
grid[f'{METRIC_COL}_sunrise_instagram_rank'] = rank_series(grid[f'{METRIC_COL}_sunrise_instagram'])
```

### Visualize relationship Flickr/Instagram¶

In [39]:

```
grid[preview_mask].drop(columns=['geometry']).head()
```

Out[39]:

|  |  | postcount\_est\_sunset | usercount\_est\_sunset\_flickr | userdays\_est\_sunset | usercount\_est\_sunrise\_flickr | postcount\_est\_sunrise | userdays\_est\_sunrise | usercount\_est\_sunrise\_instagram | usercount\_est\_sunset\_instagram | usercount\_est\_sunset\_flickr\_rank | usercount\_est\_sunset\_instagram\_rank | usercount\_est\_sunrise\_flickr\_rank | usercount\_est\_sunrise\_instagram\_rank |
| --- | --- | --- | --- | --- | --- | --- | --- | --- | --- | --- | --- | --- | --- |
| xbin | ybin |  |  |  |  |  |  |  |  |  |  |  |  |
| -15240096 | 2679952 | 11300 | 2115 | 4259 | 369 | 1242 | 1175 | 2663 | 16941 | 9065.0 | 10653.0 | 6487.5 | 8620.5 |
| -15140096 | 2679952 | 2946 | 728 | 1184 | 369 | 1758 | 1782 | 3345 | 9862 | 8943.0 | 10597.0 | 6487.5 | 8646.0 |
|  | 2579952 | 8594 | 1811 | 3696 | 254 | 1034 | 996 | 1039 | 8642 | 9056.0 | 10575.0 | 6428.0 | 8446.0 |
| -15040096 | 2579952 | 1637 | 474 | 747 | 642 | 2933 | 2855 | 1721 | 2531 | 8813.5 | 10280.0 | 6539.0 | 8551.0 |
| -10640096 | 4579952 | 45923 | 7592 | 20481 | 2030 | 9931 | 9037 | 7613 | 33931 | 9092.0 | 10678.0 | 6566.0 | 8678.0 |

**Sunset**

In [40]:

```
title = (
    f'Relationship between {METRIC} per grid cell (ranked) \n'
    'from Flickr and Instagram for sunset. ')
x_col = f'{METRIC_COL}_sunset_flickr_rank'
y_col = f'{METRIC_COL}_sunset_instagram_rank'
relationship_plot(
    data=grid, title=title, x_col=x_col, y_col=y_col,
    x_label=f'{METRIC.title()} Flickr (ranked)', y_label=f'{METRIC.title()} Instagram (ranked)')
```

Covariance including non-significant:

In [41]:

```
Covariance = np.cov(
    grid[f'{METRIC_COL}_sunset_flickr'],
    grid[f'{METRIC_COL}_sunset_instagram'])[0][1]
print(Covariance)
```

```
73131.39742788515
```

**Sunrise**

In [42]:

```
title = (
    f'Relationship between {METRIC} per grid cell (ranked) \n'
    'from Flickr and Instagram for sunrise. ')
x_col = f'{METRIC_COL}_sunrise_flickr_rank'
y_col = f'{METRIC_COL}_sunrise_instagram_rank'
relationship_plot(
    data=grid, title=title, x_col=x_col, y_col=y_col,
    x_label=f'{METRIC.title()} Flickr (ranked)', y_label=f'{METRIC.title()} Instagram (ranked)')
```

In [43]:

```
Covariance = np.cov(
    grid[f'{METRIC_COL}_sunrise_flickr'],
    grid[f'{METRIC_COL}_sunrise_instagram'])[0][1]
print(Covariance)
```

```
4628.057034252609
```

## Relationships on Country aggregate¶

Instead of using 100 km bins, relationships can also be studied for country level aggregate data (chi, total, expected etc.).

### Load Flickr country data for sunset/sunrise¶

In [44]:

```
def load_country_csv(
        topic: str = "sunrise", source: str = "flickr",
        metric: str = METRIC, output: Path = OUTPUT) -> pd.DataFrame:
    """Load country hll cardinalities for metric"""
    df = pd.read_csv(
        output / "csv" / f"countries_{metric}_chi_{source}_{topic}.csv",
        index_col=["SU_A3"])
    return df
```

In [45]:

```
df_sunrise = load_country_csv(topic="sunrise", source="flickr", metric="usercount")
```

In [46]:

```
df_sunrise.head()
```

Out[46]:

|  | usercount\_est\_expected | usercount\_est | chi\_value | significant |
| --- | --- | --- | --- | --- |
| SU\_A3 |  |  |  |  |
| ZWE | 1345.0 | 33.0 | -7.157880 | True |
| ZMB | 2511.0 | 62.0 | -9.523682 | True |
| YEM | 740.0 | 12.0 | -12.732712 | True |
| YES | 93.0 | 4.0 | 3.962289 | True |
| VNM | 31437.0 | 597.0 | -66.855308 | True |

In [47]:

```
df_sunset = load_country_csv(topic="sunset", source="flickr", metric="usercount")
```

In [48]:

```
df_sunset.head()
```

Out[48]:

|  | usercount\_est\_expected | usercount\_est | chi\_value | significant |
| --- | --- | --- | --- | --- |
| SU\_A3 |  |  |  |  |
| ZWE | 1345.0 | 158.0 | 10.816126 | True |
| ZMB | 2511.0 | 260.0 | 7.085320 | True |
| YEM | 740.0 | 38.0 | -11.804480 | True |
| YES | 93.0 | 11.0 | 2.929983 | False |
| VNM | 31437.0 | 1442.0 | -87.653996 | True |

In [49]:

```
rank_cols_dfs(df_sunrise, df_sunset, metric=METRIC)
```

In [50]:

```
df = merge_df_topics(df_sunrise, df_sunset, metric=METRIC)
```

Replace NaN values with 0:

In [51]:

```
df.fillna(0, inplace=True)
```

In [52]:

```
df.head()
```

Out[52]:

|  | usercount\_sunrise\_rank | usercount\_sunset\_rank |
| --- | --- | --- |
| SU\_A3 |  |  |
| ZWE | 118.0 | 139.5 |
| ZMB | 154.0 | 163.5 |
| YEM | 72.0 | 73.5 |
| YES | 34.5 | 38.5 |
| VNM | 233.0 | 233.0 |

### Visualize¶

In [53]:

```
f, ax = plt.subplots(figsize=(7, 7))

x_col = f'{METRIC}_sunrise_rank'
y_col = f'{METRIC}_sunset_rank'

f.suptitle(
    f'Relationship between sunset and sunrise (usercount, ranked) for Flickr',
    fontsize=12, y=0)

scatterplot_kwarg = {
    "ax":ax,
    "edgecolors":"white",
    "linewidth":1,
    "x":x_col,
    "y":y_col,
}
    
g = sns.scatterplot(
    data=df, **scatterplot_kwarg, color='grey',
    label="Country (su_a3)")

annotate(
    data=df,
    x_col=x_col,
    y_col=y_col,
    ranked=True)

ax.set_xlabel(f'Usercount Sunrise (ranked)')
ax.set_ylabel(f'Usercount Sunset (ranked)')
```

Out[53]:

```
Text(0, 0.5, 'Usercount Sunset (ranked)')
```

See if there is any conglomeration for European Countries and US/Canada.

Get list of European and North America Countries

In [54]:

```
world = gp.read_file(
    gp.datasets.get_path('naturalearth_lowres'),
    crs=CRS_WGS)
world = world.to_crs(CRS_PROJ)
```

In [55]:

```
cont_sel = world[(world["continent"].isin(
    ["Europe"])) | (world["iso_a3"] == "USA") | (world["iso_a3"] == "CAN")]
```

In [56]:

```
cont_sel.plot()
```

Out[56]:

```
<AxesSubplot:>
```

In [57]:

```
cont_sel.head()
```

Out[57]:

|  | pop\_est | continent | name | iso\_a3 | gdp\_md\_est | geometry |
| --- | --- | --- | --- | --- | --- | --- |
| 3 | 37589262.0 | North America | Canada | CAN | 1736425 | MULTIPOLYGON (((-9464830.276 5768352.350, -947... |
| 4 | 328239523.0 | North America | United States of America | USA | 21433226 | MULTIPOLYGON (((-9464830.276 5768352.350, -924... |
| 18 | 144373535.0 | Europe | Russia | RUS | 1699876 | MULTIPOLYGON (((8769033.872 7865244.997, 87094... |
| 21 | 5347896.0 | Europe | Norway | NOR | 403336 | MULTIPOLYGON (((505060.605 8505913.475, 506529... |
| 43 | 67059887.0 | Europe | France | FRA | 2715518 | MULTIPOLYGON (((-5168883.083 513724.093, -5231... |

In [58]:

```
ne_path = Path.cwd().parents[0] / "resources" / "naturalearth"
ne_filename = "ne_50m_admin_0_map_subunits.zip"
world_su = gp.read_file(
    ne_path / ne_filename.replace(".zip", ".shp"))
world_su = world_su.to_crs(CRS_PROJ)
```

In [59]:

```
def drop_cols_except(df: pd.DataFrame, columns_keep: List[str]):
    """Drop all columns from DataFrame except those specified in cols_except"""
    df.drop(
        df.columns.difference(columns_keep), axis=1, inplace=True)
```

In [60]:

```
columns_keep = ['geometry','ADMIN', 'SU_A3']
drop_cols_except(world_su, columns_keep)
```

Classify dataframe chi countries based on country list:

In [61]:

```
from geopandas.tools import sjoin
cont_sel = sjoin(
    cont_sel, world_su, 
    how='left')
```

For some reason, there is one outlier (French Guayana) that is manually excluded.

In [62]:

```
def spatial_join_area(df, cont_sel, area_context="Europe/North America"):
    "Classify dataframe chi countries based on country list"
    df[area_context] = np.where(
        ((df.index.isin(cont_sel["SU_A3"])) & (df.index != "BRA")), True, False)
```

In [63]:

```
spatial_join_area(df, cont_sel)
```

Standard annotate:

In [64]:

```
def annotate_countries(
    df: pd.DataFrame, x_col: str, y_col: str):
    """Annotate map based on a list of countries"""
    for idx, row in df.iterrows():
        plt.annotate(
            text=f'{idx}',
            xy=(row[x_col], row[y_col]),
            xytext=(-15, -15), textcoords='offset points',
            horizontalalignment='left',
            color="darkgrey")
```

There is a package callec adjust\_text that tries to reduce overlapping annotations in mpl. This will take more time, however.

In [65]:

```
def annotate_countries_adjust(
    df: pd.DataFrame, x_col: str, y_col: str, ax):
    """Annotate map based on a list of countries"""
    texts = []
    for idx, row in df.iterrows():
        texts.append(
             plt.text(
                 s=f'{idx}',
                 x=row[x_col],
                 y=row[y_col],
                 horizontalalignment='center',
                 color="darkgrey"))
    adjust_text(
        texts, autoalign='y', ax=ax,
        arrowprops=dict(arrowstyle="simple, head_width=0.25, tail_width=0.05",
                        color='r', lw=0.5, alpha=0.5))
```

In [66]:

```
def country_rel_plot(
    df: pd.DataFrame, topic1="flickr", topic2="instagram",
    plot_context="Flickr",
    filename: str = None,
    output: Path = OUTPUT,
    metric = METRIC,
    annotate_countries: bool = None,
    mask_zero: bool = True,
    add_labels: bool = False,
    ranked: bool = True):
    """Country chi square relationship plot"""
    fig, ax = plt.subplots(figsize=(7, 7))
    
    _rank = ""
    if ranked:
        _rank = "_rank"
    x_col = f'{metric}_{topic1}{_rank}'
    y_col = f'{metric}_{topic2}{_rank}'
    
    fig.suptitle(
        f'Relationship between {metric} (ranked) for {topic1} and '
        f'{topic2} per country for {plot_context}.',
        fontsize=12, y=0)

    scatterplot_kwarg = {
        "ax":ax,
        "edgecolors":"white",
        "linewidth":1,
        "x":x_col,
        "y":y_col,
    }
    
    if annotate_countries:
        df_anot = df
        if mask_zero:
            _mask_zero = ((df[x_col] == 0) & (df[y_col] == 0))
            df_anot = df[~_mask_zero]
        g = sns.scatterplot(
            data=df_anot[df_anot["Europe/North America"] == False],
            color="grey", label="Country (su_a3)",
            **scatterplot_kwarg)
        g = sns.scatterplot(
            data=df_anot[df_anot["Europe/North America"] == True],
            color="red", label="European Countries \n+ US/Canada",
            **scatterplot_kwarg)
    else:
        g = sns.scatterplot(
            data=df,
            color="grey", label="Country (su_a3)",
            **scatterplot_kwarg)        
    kws = {
    "ax":ax, "x":x_col, "y":y_col, "s": 100, 
    "facecolors": "none", "linewidth": 0.5,
    "color":"none"}

    ax.set_xlabel(f'{metric.capitalize()} {topic1.capitalize()} {"(ranked)" if _rank else ""}')
    ax.set_ylabel(f'{metric.capitalize()} {topic2.capitalize()} {"(ranked)" if _rank else ""}')
    
    if annotate_countries and add_labels:
        annotate_countries_adjust(
            df_anot[(df_anot["Europe/North America"] == True)],
            x_col=x_col,
            y_col=y_col,
            ax=ax)    
    annotate(
        data=df,
        x_col=x_col,
        y_col=y_col,
        ranked=True)
    if filename:
        print("Storing figure as png and svg..")
        fig.savefig(
            output / f"figures" / f"{filename}.png", dpi=300, format='PNG',
            bbox_inches='tight', pad_inches=1, facecolor="white")
        # also save as svg
        fig.savefig(
            output / "svg" / f"{filename}.svg", format='svg',
            bbox_inches='tight', pad_inches=1, facecolor="white")
```

In [67]:

```
def annotate_locations(
    df: pd.DataFrame):
    """Annotate map based on a list of locations"""
    for idx, row in df.iterrows():
        plt.annotate(
            text=f'{idx + 1}', # row['name']
            xy=row['coords'],
            xytext=np.subtract(row['coords'], 750000),
            horizontalalignment='left')
```

In [68]:

```
import matplotlib.patheffects as pe
from matplotlib.lines import Line2D
from adjustText import adjust_text
def annotate_records_adjust(
    df: pd.DataFrame, ax):
    """Annotate map based on a list of records"""
    texts = []
    for idx, row in df.iterrows():
        fcolor = 'black'
        if row.metric_outlier == True:
            fcolor = 'red'
        texts.append(
            plt.text(
                s=row['name'].replace(" ", "\n"), 
                x=row['coords'][0],
                y=row['coords'][1],
                horizontalalignment='center',
                color=fcolor,
                alpha=0.8,
                fontsize=10,
                path_effects=[pe.withStroke(linewidth=4, foreground="white")]))
    adjust_text(
        texts, autoalign='y', ax=ax,
        arrowprops=dict(arrowstyle="simple, head_width=0.25, tail_width=0.05",
                        color='r', lw=0.5, alpha=0.5))
```

In [69]:

```
df[df["Europe/North America"]].head()
```

Out[69]:

|  | usercount\_sunrise\_rank | usercount\_sunset\_rank | Europe/North America |
| --- | --- | --- | --- |
| SU\_A3 |  |  |  |
| VAT | 0.0 | 0.0 | True |
| USK | 218.0 | 221.0 | True |
| USH | 264.0 | 273.0 | True |
| USB | 276.0 | 285.0 | True |
| WLS | 255.0 | 263.0 | True |

Plot map

In [70]:

```
country_rel_plot(
    df, plot_context="Flickr", topic1="sunrise", topic2="sunset", annotate_countries=True,
    filename="sunrise_sunset_relationship_countries_flickr", metric=METRIC)
```

```
Storing figure as png and svg..
```

### Repeat for Instagram¶

sunset/sunrise

In [71]:

```
METRIC = "usercount"
load_kwds = {"topic":"sunrise", "source":"instagram"}
df_sunrise = load_country_csv(metric=METRIC, **load_kwds)
```

In [72]:

```
load_kwds["topic"] = "sunset"
df_sunset = load_country_csv(metric=METRIC, **load_kwds)
```

In [73]:

```
def rename_cols(df1, df2, topic1="sunset", topic2="sunrise", metric=METRIC):
    """Rename columns of for two topic comparison
    E.g.: sunset, sunrise; flickr, instagram
    """
    df1.rename(columns={
        f'{metric}':f'{metric}_{topic1}'}, inplace=True)
    df2.rename(columns={
        f'{metric}':f'{metric}_{topic2}'}, inplace=True)
```

In [74]:

```
def join_dfs_apply(df1, df2, topic1="flickr", topic2="instagram", metric=METRIC, ranked: bool = True) -> pd.DataFrame:
    """Join sunset and sunrise df chi"""
    if ranked:
        rank_cols_dfs(df1, df2, topic1, topic2, metric=metric)
    else:
        rename_cols(df1, df2, topic1, topic2, metric=metric)
    df = merge_df_topics(df1, df2, topic1, topic2, metric=metric, ranked=ranked)    
    df.fillna(0, inplace=True)
    spatial_join_area(df, cont_sel)
    return df
```

In [75]:

```
df = join_dfs_apply(df_sunrise, df_sunset, topic1="sunrise", topic2="sunset", metric=METRIC)
```

In [76]:

```
country_rel_plot(
    df, plot_context=f"{load_kwds.get('source').title()}", topic1="sunrise", topic2="sunset", annotate_countries=True,
    filename=f"sunrise_sunset_relationship_countries_instagram", metric=METRIC)
```

```
Storing figure as png and svg..
```

### Repeat for Instagram and Flickr¶

Here, we compare reliability for results with usercount for Instagram and Flickr

In [77]:

```
METRIC = "usercount"
load_kwds = {"topic":"sunrise", "source":"flickr"}
df_flickr = load_country_csv(metric=METRIC, **load_kwds)
```

In [78]:

```
load_kwds["source"] = "instagram"
df_instagram = load_country_csv(metric=METRIC, **load_kwds)
```

Repeat the process equal to Flickr, afterwards plot:

In [79]:

```
df = join_dfs_apply(df_flickr, df_instagram, topic1="flickr", topic2="instagram", metric=METRIC)
```

In [80]:

```
country_rel_plot(
    df, plot_context=f"Sunrise reactions", annotate_countries=True, topic1="flickr", topic2="instagram",
    filename=f"instagram_flickr_relationship_countries_sunrise", metric=METRIC)
```

```
Storing figure as png and svg..
```

### Repeat for Instagram/Flickr bias¶

In [81]:

```
METRIC = 'usercount'
METRIC_COL = 'usercount_est'
load_kwds = {"topic":"sunset", "source":"flickr"}
df_flickr = load_country_csv(metric="usercount", **load_kwds)
```

In [82]:

```
load_kwds["source"] = "instagram"
df_instagram = load_country_csv(metric="usercount", **load_kwds)
```

In [83]:

```
df = join_dfs_apply(
    df_flickr, df_instagram, topic1="flickr", topic2="instagram", metric=METRIC)
```

In [84]:

```
df.head()
```

Out[84]:

|  | usercount\_flickr\_rank | usercount\_instagram\_rank | Europe/North America |
| --- | --- | --- | --- |
| SU\_A3 |  |  |  |
| ZWE | 139.5 | 112.0 | False |
| ZMB | 163.5 | 131.0 | False |
| YEM | 73.5 | 50.0 | False |
| YES | 38.5 | 18.5 | False |
| VNM | 233.0 | 239.0 | False |

In [85]:

```
country_rel_plot(
    df, topic1="flickr", topic2="instagram",
    plot_context="Sunset reactions",
    filename="instagram_flickr_relationship_countries_sunset", annotate_countries=True)
```

```
Storing figure as png and svg..
```

## Relationships for Chi¶

Besides absolute values, also compare chi values for countries (sunset/sunrise and flickr/instagram)

In [86]:

```
METRIC = 'usercount'
METRIC_COL = 'usercount_est'
load_kwds = {"topic":"sunset", "source":"flickr"}
df_sunset = load_country_csv(metric='usercount', **load_kwds)
```

In [87]:

```
load_kwds["topic"] = "sunrise"
df_sunrise = load_country_csv(metric="usercount", **load_kwds)
```

In [88]:

```
df = join_dfs_apply(
    df_sunrise, df_sunset, topic1="sunrise", topic2="sunset", metric="chi_value", ranked=False)
```

In [89]:

```
df.head()
```

Out[89]:

|  | chi\_value\_sunrise | chi\_value\_sunset | Europe/North America |
| --- | --- | --- | --- |
| SU\_A3 |  |  |  |
| ZWE | -7.157880 | 10.816126 | False |
| ZMB | -9.523682 | 7.085320 | False |
| YEM | -12.732712 | -11.804480 | False |
| YES | 3.962289 | 2.929983 | False |
| VNM | -66.855308 | -87.653996 | False |

In [90]:

```
country_rel_plot(
    df, topic1="sunrise", topic2="sunset", metric='chi_value', ranked=False,
    plot_context="Chi value Flickr",
    filename="sunrise_sunset_relationship_countries_flickr_chi", annotate_countries=True)
```

```
Storing figure as png and svg..
```

In [91]:

```
METRIC = 'usercount'
METRIC_COL = 'usercount_est'
load_kwds = {"topic":"sunset", "source":"flickr"}
df_sunset = load_country_csv(metric='usercount', **load_kwds)
```

In [92]:

```
load_kwds["source"] = "instagram"
df_sunrise = load_country_csv(metric="usercount", **load_kwds)
```

In [93]:

```
df = join_dfs_apply(
    df_sunrise, df_sunset, topic1="sunrise", topic2="sunset", metric="chi_value", ranked=False)
```

In [94]:

```
country_rel_plot(
    df, topic1="sunrise", topic2="sunset", metric='chi_value', ranked=False,
    plot_context="Chi value Instagram",
    filename="sunrise_sunset_relationship_countries_instagram_chi", annotate_countries=True)
```

```
Storing figure as png and svg..
```

In [95]:

```
METRIC = 'usercount'
METRIC_COL = 'usercount_est'
load_kwds = {"topic":"sunrise", "source":"flickr"}
df_flickr = load_country_csv(metric='usercount', **load_kwds)
```

In [96]:

```
load_kwds["source"] = "instagram"
df_instagram = load_country_csv(metric="usercount", **load_kwds)
```

In [97]:

```
df = join_dfs_apply(
    df_flickr, df_instagram, topic1="flickr", topic2="instagram", metric="chi_value", ranked=False)
```

In [98]:

```
country_rel_plot(
    df, topic1="flickr", topic2="instagram", metric='chi_value', ranked=False,
    plot_context="Chi value Sunrise",
    filename="instagram_flickr_relationship_countries_sunrise_chi", annotate_countries=True)
```

```
Storing figure as png and svg..
```

In [99]:

```
METRIC = 'usercount'
METRIC_COL = 'usercount_est'
load_kwds = {"topic":"sunset", "source":"flickr"}
df_flickr = load_country_csv(metric='usercount', **load_kwds)
```

In [100]:

```
load_kwds["source"] = "instagram"
df_instagram = load_country_csv(metric="usercount", **load_kwds)
```

In [101]:

```
df = join_dfs_apply(
    df_flickr, df_instagram, topic1="flickr", topic2="instagram", metric="chi_value", ranked=False)
```

In [102]:

```
country_rel_plot(
    df, topic1="flickr", topic2="instagram", metric='chi_value', ranked=False,
    plot_context="Chi value Sunset",
    filename="instagram_flickr_relationship_countries_sunrise_chi", annotate_countries=True)
```

```
Storing figure as png and svg..
```

## Store generated graphics as tabbed HTML¶

In [103]:

```
import ipywidgets as widgets
# dictionary with filename and title
pathrefs = {
    0: ('sunrise_sunset_relationship_countries_flickr.png', 'F Sunrise + Sunset'),
    1: ('sunrise_sunset_relationship_countries_instagram.png', 'I Sunrise + Sunset'),
    2: ('instagram_flickr_relationship_countries_sunrise.png', 'I Sunrise + F Sunrise'),
    3: ('instagram_flickr_relationship_countries_sunset.png', 'I Sunset + F Sunset'),}

widgets_images = [
    widgets.Image(
        value=open(Path('OUT') / OUTPUT / f"figures" / pathref[0], "rb").read(),
        format='png',
        width=700
     )
    for pathref in pathrefs.values()]
```

In [104]:

```
from ipywidgets.embed import embed_minimal_html
children = widgets_images
tab = widgets.Tab()
tab.children = children
for i in range(len(children)):
    tab.set_title(i, pathrefs[i][1])
embed_minimal_html(
    Path('OUT') / OUTPUT / f'html{km_size_str}' / 'compare_relationships.html',
    views=[tab], title=f'Relationship plots for sunset, sunrise, flickr, and instagram absolute {METRIC} for the country level.')
```

# Create notebook HTML¶

In [108]:

```
!jupyter nbconvert --to html_toc \
    --output-dir=../out/html ./08_relationships.ipynb \
    --template=../nbconvert.tpl \
    --ExtractOutputPreprocessor.enabled=False >&- 2>&- # create single output file
```

Copy single HTML file to resource folder

In [109]:

```
!cp ../out/html/08_relationships.html ../resources/html/
```

In [ ]:

```

```
