# Supplementary material for: From sunrise to sunset: Exploring landscape preference through global reactions to ephemeral events captured in georeferenced social media
Source: PLoS One. 2023 Feb 22;18(2):e0280423. doi: 10.1371/journal.pone.0280423 (PMC9946259; doi:10.1371/journal.pone.0280423)
Supplement: S9 File — (HTML) [file pone.0280423.s009.html]

09\_statistics


# Statistics overview sunset/sunrise¶

*Alexander Dunkel, TU Dresden, Institute of Cartography; Maximilian Hartmann, Universität Zürich (UZH), Geocomputation*

---

•••

Out[1]:

Last updated: Jan-17-2023, Carto-Lab Docker Version 0.9.0

Several additional quantities/numbers are collected here and referenced in the article.

# Preparations¶

## Load dependencies¶

This time, we use the python\_hll package to calculate hll set cardinalities.  
`python_hll` is significantly slower than the native Postgres HLL implementation.  
But there are only a few temporal HLL sets to calculate (year and months aggregates).

In [2]:

```
import sys
import pandas as pd
from pathlib import Path
from python_hll.hll import HLL
from python_hll.util import NumberUtil
module_path = str(Path.cwd().parents[0] / "py")
if module_path not in sys.path:
    sys.path.append(module_path)
from modules import tools, preparations
from _03_chimaps import OUTPUT
```

```
Chromedriver loaded. Svg output enabled.
```

## Load HLL aggregate data¶

Data is stored as aggregate HLL data (postcount) for each term.

There is an additional CSV that contains the HLL set with all Flickr posts (2007-2018).

In [3]:

```
root = Path.cwd().parents[1] / "00_hll_data"
TERMS_INSTAGRAM = root / "instagram-terms.csv"
TERMS_FLICKR = root / "flickr-terms.csv"
ALL_FLICKR = root / "flickr-all.csv"
```

Some statistics for these files:

In [4]:

```
%%time
data_files = {
    "TERMS_INSTAGRAM":TERMS_INSTAGRAM,
    "TERMS_FLICKR":TERMS_FLICKR,
    "ALL_FLICKR":ALL_FLICKR,
    }
tools.display_file_stats(data_files)
```

| name | TERMS\_INSTAGRAM | TERMS\_FLICKR | ALL\_FLICKR |
| --- | --- | --- | --- |
| size | 49.65 KB | 46.87 KB | 2.55 KB |
| records | 24 | 24 | 2 |

```
CPU times: user 21.3 ms, sys: 7.59 ms, total: 28.9 ms
Wall time: 28.2 ms
```

Preview CSVs:

In [5]:

```
display(pd.read_csv(ALL_FLICKR))
```

|  | name | post\_hll |
| --- | --- | --- |
| 0 | Flickr all posts 2007-2017 | \x148b409c25094232946329c6349d2d19c610b4272942... |

In [6]:

```
df = pd.read_csv(TERMS_INSTAGRAM)
```

In [7]:

```
display(df)
```

|  | term | topic | post\_hll |
| --- | --- | --- | --- |
| 0 | sunrise | sunrise | \x148b405b16d6318a5c5ee7358c7bd6c6b60f5c18a6ad... |
| 1 | sunrises | sunrise | \x148b40324e731d0839ce9294e631927498a7318a5398... |
| 2 | sonnenaufgang | sunrise | \x148b4041ce9498c75bde6324c741ce7398e7398c94a0... |
| 3 | leverdusoleil | sunrise | \x148b4010c05084c520823180831882418c4108c01198... |
| 4 | leverdesoleil | sunrise | \x148b40294210988430c453148210423110c228c6318c... |
| 5 | leversoleil | sunrise | \x138b40004300c100e102410282032103a203e1048104... |
| 6 | lever\_du\_soleil | sunrise | \x138b400a010f620f8110e3118116c31fc22041226123... |
| 7 | lever\_de\_soleil | sunrise | \x138b400102036103c205c107210be20ca210a1128318... |
| 8 | lever\_soleil | sunrise | \x138b4003e304a10ce30e850fa2112317c22cc239223c... |
| 9 | zonsopkomst | sunrise | \x148b4008c020942408c831082310d45188621a062098... |
| 10 | zonsopgang | sunrise | \x148b4008c621040220881104c2284410044508c60088... |
| 11 | lever soleil | sunrise | \x148b40088620082110c202880308401180c210c81084... |
| 12 | sunset | sunset | \x148b7f731ce7b9ae6cd6e7bd8e7b16e8b5cf6c18c6c9... |
| 13 | coucher\_du\_soleil | sunset | \x148b7f00841300a21284300801008400808200880100... |
| 14 | sunsets | sunset | \x148b7f4ad09421885b9095258a52d4b5294c5258a429... |
| 15 | sonnenuntergang | sunset | \x148b7f435086310753d885bd49521074a14a525484a5... |
| 16 | couchersoleil | sunset | \x148b7f00400184400842010420190000084008020004... |
| 17 | coucherdusoleil | sunset | \x148b7f210a3314831886229cc320c8218c6730883294... |
| 18 | coucher\_soleil | sunset | \x128b7f8592bc43e608063786896ecadc008eab89ad82... |
| 19 | zonsondergang | sunset | \x148b7f3188521082318651a525194e64106419463418... |
| 20 | coucher soleil | sunset | \x148b7f0000000c001800208000000010088000000000... |
| 21 | coucher\_de\_soleil | sunset | \x148b7f0a8621946208c64288873206220c813084530c... |
| 22 | coucherdesoleil | sunset | \x148b7f518a4610c741949421284210a2992c4190931d... |

# Calculate Statistics¶

## HLL Cardinality per term¶

**Prepare functions**

These functions were first used in the YFCC HLL Workshop.

In [8]:

```
def hll_from_byte(hll_set: str):
    """Return HLL set from binary representation"""
    hex_string = hll_set[2:]
    # hex_string = hll_set
    return HLL.from_bytes(
        NumberUtil.from_hex(
            hex_string, 0, len(hex_string)))

def cardinality_from_hll(hll_set):
    """Turn binary hll into HLL set and return cardinality"""
    try:
        hll = hll_from_byte(hll_set)
    except:
        print(hll_set)
    return hll.cardinality()
```

Define additional functions for reading and formatting CSV as `pd.DataFrame`

In [9]:

```
def append_cardinality_df(
        df: pd.DataFrame, hll_col: str = "post_hll", drop_hll_col: bool = False):
    """Calculate cardinality from HLL and append to extra column in df"""
    df['postcount_est'] = df.apply(
        lambda x: cardinality_from_hll(
           x[hll_col]),
        axis=1)
    if drop_hll_col:
        df.drop(columns=["post_hll"], inplace=True)
    return

def read_hll_csv(csv: Path, key_col: str) -> pd.DataFrame:
    """Read CSV with parsing datetime index (months)
    
        First CSV column: Year
        Second CSV column: Month
    """
    df = pd.read_csv(
        csv, index_col=key_col)
    append_cardinality_df(df)
    return df
```

In [10]:

```
%%time
import warnings; warnings.simplefilter('ignore')
df = read_hll_csv(TERMS_INSTAGRAM, key_col="term")
```

```
CPU times: user 1.27 s, sys: 23 µs, total: 1.27 s
Wall time: 1.27 s
```

**RuntimeWarning?** 

- python-hll library is in a very early stage of development
- it is not fully compatible with the citus hll implementation in postgres
- The shown RuntimeWarning (Overflow) is one of the issues that need to be resolved in the future
- If you run this notebook locally, it is recommended to use pg-hll-empty for
  any hll calculations, as is shown (e.g.) in the original YFCC100M notebooks.
- There is no significant negative impact on accuracy for this application case.

In [11]:

```
display(df[df["topic"]=="sunset"].sort_values('postcount_est', ascending=False))
display(df[df["topic"]=="sunrise"].sort_values('postcount_est', ascending=False))
```

|  | topic | post\_hll | postcount\_est |
| --- | --- | --- | --- |
| term |  |  |  |
| sunset | sunset | \x148b7f731ce7b9ae6cd6e7bd8e7b16e8b5cf6c18c6c9... | 16992174 |
| sunsets | sunset | \x148b7f4ad09421885b9095258a52d4b5294c5258a429... | 1443750 |
| sonnenuntergang | sunset | \x148b7f435086310753d885bd49521074a14a525484a5... | 351389 |
| coucherdesoleil | sunset | \x148b7f518a4610c741949421284210a2992c4190931d... | 87690 |
| zonsondergang | sunset | \x148b7f3188521082318651a525194e64106419463418... | 27318 |
| coucherdusoleil | sunset | \x148b7f210a3314831886229cc320c8218c6730883294... | 12977 |
| coucher\_de\_soleil | sunset | \x148b7f0a8621946208c64288873206220c813084530c... | 12621 |
| coucher\_du\_soleil | sunset | \x148b7f00841300a21284300801008400808200880100... | 2556 |
| couchersoleil | sunset | \x148b7f00400184400842010420190000084008020004... | 2556 |
| coucher soleil | sunset | \x148b7f0000000c001800208000000010088000000000... | 802 |
| coucher\_soleil | sunset | \x128b7f8592bc43e608063786896ecadc008eab89ad82... | 90 |

|  | topic | post\_hll | postcount\_est |
| --- | --- | --- | --- |
| term |  |  |  |
| sunrise | sunrise | \x148b405b16d6318a5c5ee7358c7bd6c6b60f5c18a6ad... | 4662612 |
| sunrises | sunrise | \x148b40324e731d0839ce9294e631927498a7318a5398... | 126115 |
| sonnenaufgang | sunrise | \x148b4041ce9498c75bde6324c741ce7398e7398c94a0... | 103460 |
| leverdesoleil | sunrise | \x148b40294210988430c453148210423110c228c6318c... | 11495 |
| zonsopkomst | sunrise | \x148b4008c020942408c831082310d45188621a062098... | 6770 |
| leverdusoleil | sunrise | \x148b4010c05084c520823180831882418c4108c01198... | 6081 |
| zonsopgang | sunrise | \x148b4008c621040220881104c2284410044508c60088... | 3423 |
| lever soleil | sunrise | \x148b40088620082110c202880308401180c210c81084... | 3387 |
| leversoleil | sunrise | \x138b40004300c100e102410282032103a203e1048104... | 525 |
| lever\_du\_soleil | sunrise | \x138b400a010f620f8110e3118116c31fc22041226123... | 73 |
| lever\_de\_soleil | sunrise | \x138b400102036103c205c107210be20ca210a1128318... | 68 |
| lever\_soleil | sunrise | \x138b4003e304a10ce30e850fa2112317c22cc239223c... | 53 |

In [12]:

```
df_instagram = df
```

In [13]:

```
%%time
import warnings; warnings.simplefilter('ignore')
df = read_hll_csv(TERMS_FLICKR, key_col="term")
```

```
CPU times: user 1.32 s, sys: 0 ns, total: 1.32 s
Wall time: 1.32 s
```

In [14]:

```
display(df[df["topic"]=="sunset"].sort_values('postcount_est', ascending=False))
display(df[df["topic"]=="sunrise"].sort_values('postcount_est', ascending=False))
df_flickr = df
```

|  | topic | post\_hll | postcount\_est |
| --- | --- | --- | --- |
| term |  |  |  |
| sunset | sunset | \x148b7f5a9ac5354a5b18c7318b5498e83dcf63a2b639... | 2431495 |
| sonnenuntergang | sunset | \x148b7f398a7398cc31ce7314c631d2e39d063a505395... | 112093 |
| sunsets | sunset | \x148b7f418cc2a4c4414c5614e632183418e6318e4299... | 70104 |
| coucherdesoleil | sunset | \x148b7f294853a065214e33946630cc43908629044194... | 30755 |
| zonsondergang | sunset | \x148b7f394a33988a210c719866210e43986618ca6314... | 25306 |
| coucher soleil | sunset | \x148b7f214433986828c8722466194832210320c44218... | 19604 |
| coucherdusoleil | sunset | \x148b7f38867190241082220c4330443208a228c20114... | 7035 |
| coucher\_de\_soleil | sunset | \x148b7f18002084400080008402080642100200002100... | 2144 |
| couchersoleil | sunset | \x148b7f10400000010080000020080020000100020000... | 1111 |
| coucher\_du\_soleil | sunset | \x148b7f00001180000000200c00084000000009400000... | 1078 |
| coucher\_soleil | sunset | \x128b7f81333b36e434391489d6ff166ddc75638cbb9f... | 31 |

|  | topic | post\_hll | postcount\_est |
| --- | --- | --- | --- |
| term |  |  |  |
| sunrise | sunrise | \x148b7f4a9884a94762128521696a52a5252c629696a8... | 851468 |
| sonnenaufgang | sunrise | \x148b7f3192639ce44a0a54908329924294c420d25290... | 41514 |
| leverdesoleil | sunrise | \x148b7f0a0430984518cc409d2210824314a040867188... | 10283 |
| sunrises | sunrise | \x148b7f09046108231908209442288231106109062298... | 9627 |
| lever soleil | sunrise | \x148b7f1a0420a0e41800421464088431182120cc3200... | 6750 |
| zonsopkomst | sunrise | \x148b7f18485098410882209844204421086109404210... | 5163 |
| zonsopgang | sunrise | \x148b7f1940200c420882100481080421040208465200... | 3527 |
| leverdusoleil | sunrise | \x148b7f3886040401284202880310c23000a028020000... | 2050 |
| leversoleil | sunrise | \x138b7f03c1070107610781092109e10a270a610ac30c... | 187 |
| lever\_du\_soleil | sunrise | \x118b7f | 0 |
| lever\_de\_soleil | sunrise | \x118b7f | 0 |
| lever\_soleil | sunrise | \x118b7f | 0 |

## Total counts¶

The HLL union operation is lossless. Therefore, all hll sets (post\_hll) can be unioned, to calculate the total cardinality for Instagram and Flickr data.

The function below first appeared in Dunkel et al. (2020)

In [15]:

```
def union_hll(hll: HLL, hll2):
    """Union of two HLL sets. The first HLL set will be modified in-place."""
    hll.union(hll2)
    
def union_all_hll(
    hll_series: pd.Series, cardinality: bool = True) -> pd.Series:
    """HLL Union and (optional) cardinality estimation from series of hll sets

        Args:
        hll_series: Indexed series (bins) of hll sets. 
        cardinality: If True, returns cardinality (counts). Otherwise,
            the unioned hll set will be returned.
    """
    hll_set = None
    for hll_set_str in hll_series.values.tolist():
        if hll_set is None:
            # set first hll set
            hll_set = hll_from_byte(hll_set_str)
            continue
        hll_set2 = hll_from_byte(hll_set_str)
        union_hll(hll_set, hll_set2)
    return hll_set.cardinality()
```

Union and calculate cardinality

In [16]:

```
instagram_total = union_all_hll(df_instagram["post_hll"].dropna())
instagram_sunrise = union_all_hll(df_instagram[df_instagram["topic"]=="sunrise"]["post_hll"].dropna())
instagram_sunset = union_all_hll(df_instagram[df_instagram["topic"]=="sunset"]["post_hll"].dropna())
print(f"Instagram sunset-sunrise: {instagram_total:,.0f} estimated total posts")
print(f"Instagram sunset: {instagram_sunset:,.0f} estimated total posts")
print(f"Instagram sunrise: {instagram_sunrise:,.0f} estimated total posts")
```

```
Instagram sunset-sunrise: 21,192,990 estimated total posts
Instagram sunset: 17,660,472 estimated total posts
Instagram sunrise: 4,741,053 estimated total posts
```

Repeat for Flickr

In [17]:

```
flickr_total = union_all_hll(df_flickr["post_hll"].dropna())
flickr_sunrise = union_all_hll(df_flickr[df_flickr["topic"]=="sunrise"]["post_hll"].dropna())
flickr_sunset = union_all_hll(df_flickr[df_flickr["topic"]=="sunset"]["post_hll"].dropna())
print(f"Flickr sunset-sunrise: {flickr_total:,.0f} estimated total posts")
print(f"Flickr sunset: {flickr_sunset:,.0f} estimated total posts")
print(f"Flickr sunrise: {flickr_sunrise:,.0f} estimated total posts")
```

```
Flickr sunset-sunrise: 3,310,397 estimated total posts
Flickr sunset: 2,545,460 estimated total posts
Flickr sunrise: 881,324 estimated total posts
```

**Question:** Percentage of all posts captured by just using the top-scoring two terms "sunset" and "sunrise"?

In [18]:

```
sum_sunset_sunrise = union_all_hll(
    pd.Series([df_instagram["post_hll"]["sunset"], df_instagram["post_hll"]["sunrise"]]))
print(
    f"{sum_sunset_sunrise:,.0f} of Instagram posts "
    f"contain either the term 'sunset' or 'sunrise', "
    f"which is {sum_sunset_sunrise/(instagram_total/100):,.1f}% "
    "of all sunset-sunrise posts in the dataset.")
```

```
20,488,000 of Instagram posts contain either the term 'sunset' or 'sunrise', which is 96.7% of all sunset-sunrise posts in the dataset.
```

In [19]:

```
sum_sunset_sunrise = union_all_hll(
    pd.Series([df_flickr["post_hll"]["sunset"], df_flickr["post_hll"]["sunrise"]]))
print(
    f"{sum_sunset_sunrise:,.0f} of Flickr posts "
    f"contain either the term 'sunset' or 'sunrise', "
    f"which is {sum_sunset_sunrise/(flickr_total/100):,.1f}% "
    "of all sunset-sunrise posts in the dataset.")
```

```
3,207,577 of Flickr posts contain either the term 'sunset' or 'sunrise', which is 96.9% of all sunset-sunrise posts in the dataset.
```

## Instagram geotagged/non-geotagged¶

For Instagram, the total counts also contain non-geotagged.

Calculate the number of total geotagged Instagram posts in the dataset  
from the pickle generated in the first notebook (100km aggregate data):

In [20]:

```
%%time
import warnings; warnings.simplefilter('ignore')
pickle_path = Path.cwd().parents[0] / "out" / "pickles"
grid = pd.read_pickle(
    pickle_path / "instagram_postcount_sunsetsunrise_est_hll.pkl")
instagram_geotagged_total = union_all_hll(grid["postcount_hll"].dropna())
print(
    f"Instagram geotagged sunset-sunrise: "
    f"{instagram_geotagged_total:,.0f} estimated total posts")
```

```
Instagram geotagged sunset-sunrise: 9,462,266 estimated total posts
CPU times: user 2min 3s, sys: 54.8 ms, total: 2min 3s
Wall time: 2min 3s
```

## Flickr Creative Commons Sample datasets¶

The raw data containing only creative commons Flickr posts can  
be summarized by counting lines in the CSV files:

In [21]:

```
def get_line_count(csv: Path) -> int:
    """Get line count of CSV file (minus header)"""
    with open(csv) as f:
        return sum(1 for line in f) - 1
```

In [22]:

```
%%time

FLICKR_CC_SUNRISE = root / "2020-04-07_Flickr_Sunrise_World_CCBy.csv"
FLICKR_CC_SUNSET = root / "2020-04-07_Flickr_Sunset_World_CCBy.csv"

print(f'{get_line_count(FLICKR_CC_SUNRISE)} Flickr sunrise CC-BY images')
print(f'{get_line_count(FLICKR_CC_SUNSET)} Flickr sunset CC-BY images')
```

```
82852 Flickr sunrise CC-BY images
284990 Flickr sunset CC-BY images
CPU times: user 42.2 ms, sys: 0 ns, total: 42.2 ms
Wall time: 41.5 ms
```

# Create notebook HTML¶

In [23]:

```
!jupyter nbconvert --to html_toc \
    --output-dir=../out/html ./09_statistics.ipynb \
    --template=../nbconvert.tpl \
    --ExtractOutputPreprocessor.enabled=False >&- 2>&- # create single output file
```

Copy single HTML file to resource folder

In [24]:

```
!cp ../out/html/09_statistics.html ../resources/html/
```

# Create Release File¶

First convert all svg to pdf, for archive purposes and paper submission.

In [25]:

```
WEB_DRIVER = preparations.load_chromedriver()
```

```
Chromedriver loaded. Svg output enabled.
```

In [26]:

```
%%time
tools.convert_svg_pdf(in_dir=OUTPUT / "svg", out_dir=OUTPUT / "pdf")
```

```
Processed 44 of 44 files..
CPU times: user 1.37 s, sys: 7.3 s, total: 8.67 s
Wall time: 1min
```

**Create release file with all results**

Create a release file that contains ipynb notebooks, HTML, figures, svg and python converted files.

Make sure that 7z is available (`apt-get install p7zip-full`)

In [10]:

```
!cd .. && RELEASE_VERSION=$(git describe --tags --abbrev=0) \
    && 7z a -tzip -mx=9 out/release_$RELEASE_VERSION.zip \
    md/* py/* out/html/* out/pdf/* out/svg/* out/figures/* notebooks/*.ipynb \
    README.md jupytext.toml nbconvert.tpl \
    -x!py/__pycache__ -x!py/modules/__pycache__ -x!py/modules/.ipynb_checkpoints \
    -y > /dev/null
```

In [ ]:

```

```
