# Supplementary figures and images for: From sunrise to sunset: Exploring landscape preference through global reactions to ephemeral events captured in georeferenced social media
Source: PLoS One. 2023 Feb 22;18(2):e0280423. doi: 10.1371/journal.pone.0280423 (PMC9946259; doi:10.1371/journal.pone.0280423)

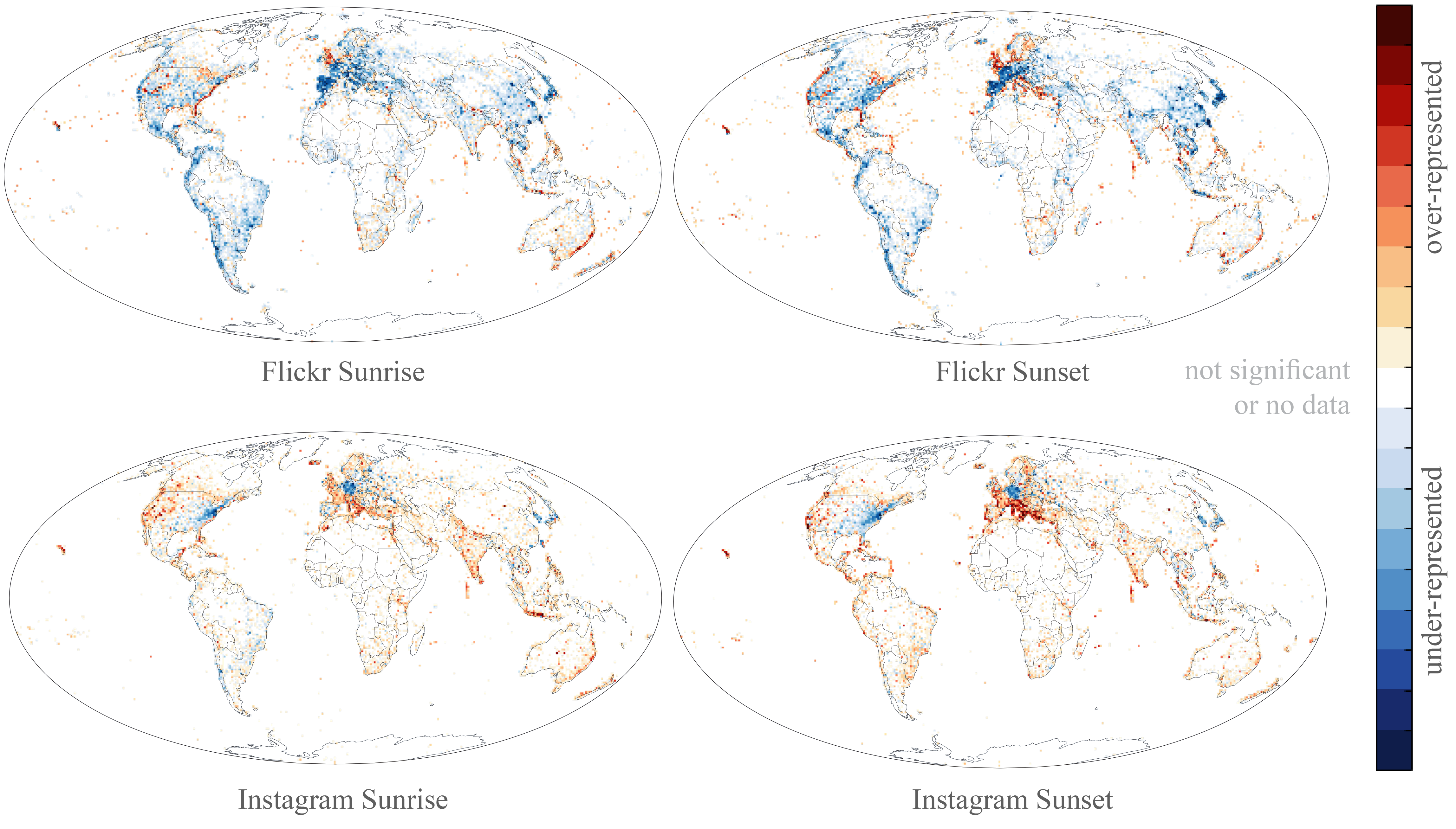

Supplement: S1 Fig — Based on user count, over- and underrepresentation, Natural Breaks classification. (TIF) [file pone.0280423.s010.tif]
